# APCI‐Multistage Mass Spectrometry Following Liquid Chromatography for Selected 4‐Desmethyl‐Sterols and Their Deuterium‐Labelled Analogues Unveils Characteristic Fragmentation Routes for Cholesterol and Phytosterols Identification

**DOI:** 10.1002/rcm.10039

**Published:** 2025-04-05

**Authors:** V. Cinquepalmi, I. Losito, A. Castellaneta, C. D. Calvano, T. R. I. Cataldi

**Affiliations:** ^1^ Dipartimento di Chimica Università degli Studi di Bari "Aldo Moro" Bari Italy; ^2^ Centro Interdipartimentale SMART Università degli Studi di Bari "Aldo Moro" Bari Italy

**Keywords:** atmospheric pressure chemical ionization, high‐resolution tandem mass spectrometry, isotopically labelled standards, multistage mass spectrometry, phytosterols

## Abstract

**Rationale:**

Several phytosterols (PSs), well known for their role in plant physiology and their health benefits, represent a subset of the family of 4‐desmethyl‐sterols. They exhibit remarkable structural variability due to differences in the number and position of C=C bonds in their tetracyclic backbone and side chain composition. When analysed using tandem mass spectrometry (MS/MS), PSs often produce complex and potentially informative spectra, as in the case of electron ionization. However, these spectra have been only partially interpreted so far. Here, a systematic interpretation of the fragmentation of PSs, specifically free sterols, was pursued through a synergic use of high‐ and low‐resolution multistage mass spectrometry (MS^n^, *n* = 2–4).

**Methods:**

The study focused on protonated and dehydrated forms of standard 4‐desmethyl‐sterols ([M + H‐H_2_O]^+^), generated via atmospheric pressure chemical ionization (APCI) following reversed‐phase liquid chromatography (RPLC). Deuterium‐labelled versions of cholesterol and stigmasterol, appropriately labelled on their side chains, were examined alongside their natural counterparts and other key PS standards, including β‐sitosterol, campesterol, brassicasterol, Δ^5^‐avenasterol (isofucosterol) and its isomer Δ^7^‐avenasterol.

**Results:**

The use of isotopically labelled standards allowed the identification of diagnostic, low *m/z*, product ions associated with the side chain, demonstrating that the positive charge can localize not only at the C3 position (associated with the hydroxyl group) but also on the side chain itself (C24/C25). Furthermore, all remaining peak signals in the tandem MS spectra of PSs were successfully elucidated with the help of MS^3^/MS^4^ measurements, unveiling complex fragmentation pathways involving both the steroidal backbone and the side chain and indicating C17 as an additional potential site for positive charge localization.

**Conclusions:**

The findings described in the paper offer a strong basis for identifying critical structural features of PSs, thus opening interesting perspectives for the identification of minor PSs, often isomeric with more common ones, that can be detected in vegetal matrices.

## 1 Introduction

**FIGURE 1:**
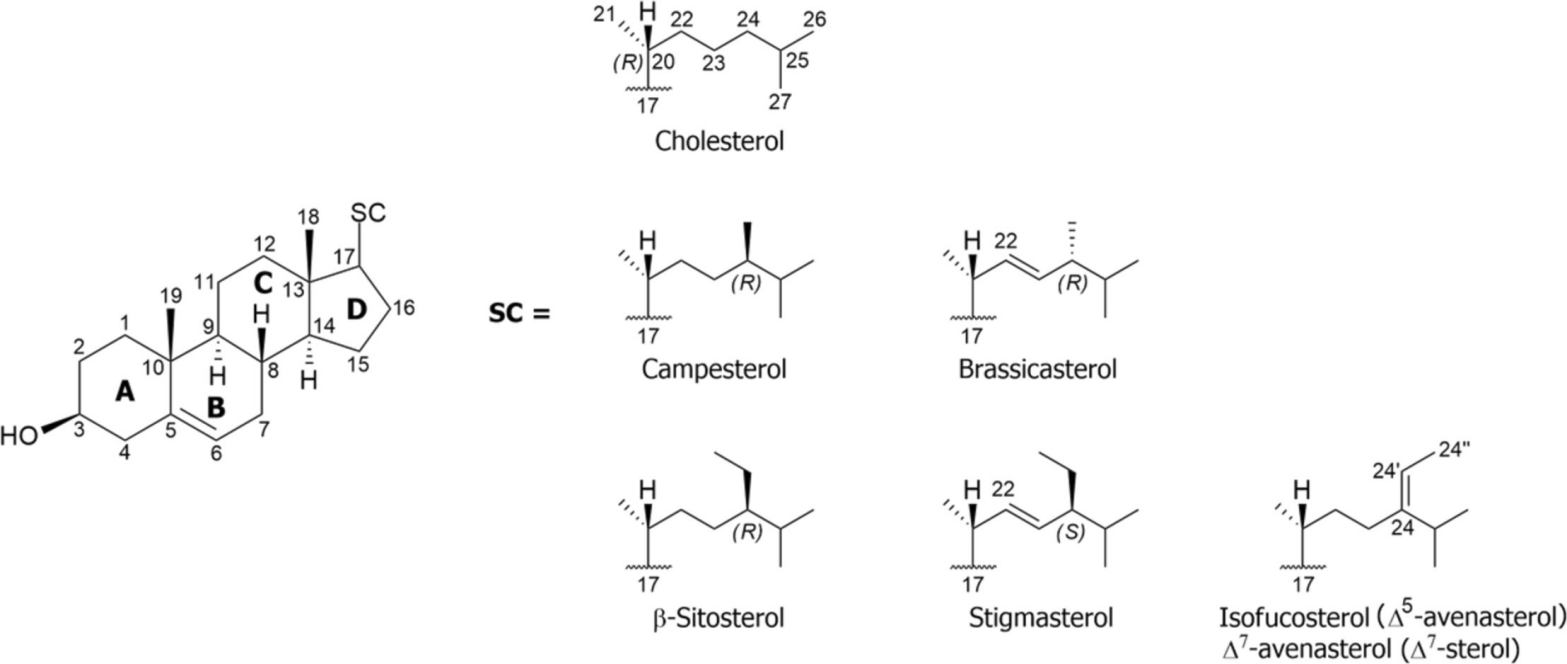
(Left side) General chemical structure of Δ^5^‐4‐desmethyl‐sterols. Carbon atoms are numbered according to the rules established by IUPAC in 1989. (Right side) Side chain (SC) structures and common names for sterols considered in the present study. Δ^7^‐avenasterol shares the side chain with isofucosterol but has the C=C double bond on a different position of Ring B (between C7 and C8). The numbering of all atoms on the side chain is shown in the case of cholesterol. The C24 configuration is highlighted in the case of campesterol, brassicasterol, β‐sitosterol and stigmasterol.

Phytosterols (PSs) are sterols occurring in plant cell membranes, particularly in raft domains [1]. These ordered and nanoscale regions, where PSs exhibit association with sphingolipids and proteins, play a role in key cellular processes such as plant growth and development, signal transduction in response to environmental stresses and cell‐to‐cell communication through plasmodesmata [2, 3]. As shown in Figure 1, PSs share the tetracyclic structure of cyclopenta[α]phenanthrene, in which *trans* junctures connect the four rings (A, B, C and D). Two methyl groups are located at C18 and C19, and a side chain (SC) is linked to C17, according to the atom numbering recommended by the International Union of Pure and Applied Chemistry (IUPAC) [3, 4, 5]. Among PSs, free sterols (FSs) are characterized by a hydroxyl group at C3 having β stereochemistry [6], which can be either esterified by a free fatty acid, giving a steryl ester, or form a 1‐*O*‐β‐glycosidic bond with a hexose, generating a steryl glycoside. If an acyl chain is bound to the C6′ of the sugar, acylated steryl glycosides are obtained [7, 8]. FSs can be generally subdivided into 4‐desmethyl, 4α‐methyl and 4,4‐dimethyl sterols, depending on the number of substituents at C4 [9], although the latter two species, acting as precursors of 4‐desmethyl sterols, are scarcely present in plant tissues [10]. Among 4‐desmethyl‐sterols, Δ^5^‐, Δ^7^‐, Δ^5,7^‐ and Δ^8^‐sterols include one or two C=C bonds on the B ring (see Figure 1), distinguishing them from stanols, their saturated counterparts [8]. However, most of the over 250 PSs identified in nature so far [11] are Δ^5^‐4‐desmethyl‐sterols, which differ from each other for their SC, as shown in Figure 1 for some of the most important FSs.

Cholesterol (CHO), the simplest species among Δ^5^‐sterols, characterized by an unsaturated and unbranched SC and representing the most abundant sterol in animal tissues, is also present, in low amounts, in plant cells [1, 12]. As evidenced in Figure 1, the presence of a methyl or an ethyl group linked to C24 of CHO leads to the structures of campesterol and β‐sitosterol, which are among the most abundant PSs [11]. The methylation reaction at C24 may generate both α and β epimers, while the introduction of an ethyl substituent preferentially produces α epimers [4]. Other relevant PSs are brassicasterol and stigmasterol, corresponding to the unsaturated analogues of campesterol and β‐sitosterol, respectively, both having a double bond on C22–C23 [5, 6] (see Figure 1). Modification of C24 may also introduce an ethylidene group, leading to isofucosterol, also known as Δ^5^‐avenasterol, representing another non‐negligible PS, often accompanied by its positional isomer Δ^7^‐avenasterol, having a C=C bond between C7 and C8 on Ring B (see Figure 1).

The structural variability of PSs poses a challenge for their qualitative/quantitative analysis in plant extracts, aiming at the assessment of their nutraceutical potential, since PSs have been reported to lower CHO levels and exert antiobesity, anti‐inflammatory and anticancer activities [13, 14, 15].

Gas chromatography coupled with electron ionization mass spectrometry (GC‐EI‐MS) has long been the conventional method for sterol analysis, including PSs. In fact, major sterols can be separated effectively using low‐medium polarity GC columns [11, 16, 17, 18, 19, 20], provided they are preliminarily derivatized to trimethylsilyl (TMS) ethers or steryl acetates to enhance volatility and ionization efficiency [21, 22]. With the advent of soft atmospheric pressure ionization (API) sources, suitable for the coupling between liquid chromatography and mass spectrometry, reversed‐phase liquid chromatography (RPLC) based on C8 or C18 stationary phases has emerged as a powerful alternative [1, 23]. This approach eliminates the risks of incomplete derivatization and thermal degradation related to GC conditions. Among API processes, electrospray ionization (ESI) is usually performed on sterols after chemical derivatizations, such as the generation of picolinic esters, aimed at boosting the ionization efficiency [24, 25, 26]. Proton and sodium adducts are usually produced by sterols under positive ESI conditions [27, 28]. Atmospheric pressure chemical ionization (APCI) in positive mode has significantly increased sensitivity (up to two orders of magnitude), leading only to the protonation of the sterols OH group, followed by in‐source loss of water (H_2_O) to form [M + H‐H_2_O]^+^ ions [9, 29, 30]. As a result, RPLC‐APCI‐MS has become a popular method for identifying FSs in food products, including plant‐based ones, although their quantification is limited to species for which standards are available [27, 31, 32, 33, 34, 35, 36, 37, 38].

Actually, the occurrence of several FS isomers, some of which do not correspond to available standards, is common in complex matrices like plant extracts. Tandem mass spectrometry (MS/MS), successfully adopted so far for the analysis of other lipid compounds, like glycerophospholipids, glycerolipids and sulfolipids [39, 40, 41, 42, 43, 44, 45, 46, 47], represents a promising approach to recognize structural features of sterols, even for species not matching available standards. However, to the best of our knowledge, only two studies have delved so far into a detailed characterization of MS/MS spectra obtained for FSs, which can be complex due to the progressive fragmentation of their structures. Jiang et al. [30] used high‐resolution APCI‐MS/MS relying on the so‐called higher collisional energy dissociation (HCD) mode enabled by the Thermo Scientific Q‐Exactive quadrupole‐Orbitrap mass spectrometer (which actually corresponds to a typical collision‐induced dissociation (CID) in terms of the ion energies involved) and APCI‐MS^n^ (with *n* = 2, 3) based on a quadrupole‐linear ion trap mass spectrometer to reconstruct the fragmentation patterns of stigmasterol, brassicasterol, β‐sitosterol and campesterol. Münger et al. [48] compared CID‐MS/MS spectra obtained for ESI‐generated positive precursor ions of the aglycones of dietary steryl glycosides with those related to protonated and dehydrated FS ions, which are isomeric with the aglycone ions. These studies highlighted product ions providing insights into the number and position of double bonds on the B ring of the sterol structure. Product ions related to neutral losses occurring from the SC structure of sterols were also evidenced [48], suggesting that a careful interpretation of the sterol complex fragmentation patterns might yield valuable structural information.

Starting from this background, a systematic evaluation has been recently undertaken in our laboratory for fragmentation patterns of specific PS standards using APCI‐tandem/sequential mass spectrometry (APCI‐MS^n^, with *n* = 2–4). These experiments were performed under different mass resolution conditions. CHO‐d_6_ and stigmasterol‐d_5_, isotopically labelled on the respective SCs, were also included among the analysed species to clarify some of the fragmentation pathways. A detailed interpretation of the fragmentation patterns observed for FSs will be discussed in the present work, with a focus on the unexpected product ions arising from the sterol SC. The latter suggested different possible locations of the positive charge of [M + H‐H_2_O]^+^ ions of FSs. The differences observed for major Δ^5^‐sterols, as well as between them and Δ^7^‐avenasterol, will also be discussed to provide general guidelines that might assist in identifying structural features of uncommon sterols, particularly when standards are unavailable.

## 2 Materials and Methods

### 2.1 Chemicals

LC grade acetonitrile (ACN), water (H_2_O) and formic acid (FA), used for RPLC separations, HPLC grade chloroform (CHCl_3_) and ethanol (EtOH), employed as solvents for stock solutions, were purchased from Merck (Milan, Italy). Standards of CHO‐d_6_ (cholest‐5‐en‐26,26,26,27,27,27‐d_6_‐3β‐ol), CHO (cholest‐5‐en‐3β‐ol), campesterol (campest‐5‐en‐3β‐ol), brassicasterol (ergosta‐5,22E‐dien‐3β‐ol), β‐sitosterol (stigmast‐5‐en‐3β‐ol), stigmasterol (stigmasta‐5,22E‐dien‐3β‐ol), isofucosterol (24Z‐ethylidene‐cholest‐5‐en‐3β‐ol) and Δ7‐avenasterol (24Z‐ethylidene‐cholest‐7‐en‐3β‐ol) were acquired from Cayman Chemical (Ann Arbor, MI, USA). Standard stigmasterol‐d_5_ (stigmasta‐5,22E‐dien‐24′,24′,24″,24″,24″‐d_5_‐3β‐ol) was purchased from Merck (Milan, Italy). The solutions required for the calibration of mass spectrometers under positive or negative polarity conditions were purchased from Thermo Scientific (Waltham, MA, USA).

### 2.2 Standard Solutions Preparation

A stock solution of each standard (1 mg/mL) was prepared in an EtOH:CHCl_3_ mixture (2:1 *v/v*), and a diluted solution (30 μg/mL) was subsequently obtained in 100% ACN. Moreover, through appropriate dilutions from stock solutions, a comprehensive mix of standards (10 μg/mL for each one) was prepared in 100% ACN.

### 2.3 RPLC‐APCI‐MS Instrumentation and Operating Conditions

Mixtures of standard sterols were analysed using an Ultimate 3000 HPLC system coupled with a Q‐Exactive high‐resolution quadrupole‐Orbitrap Fourier‐transform mass spectrometer through an APCI interface (Thermo Fisher, West Palm Beach, CA, USA). A C18 Ascentis Express HPLC column (150 × 2.1 mm id, 2.7 μm particle size) (Supelco, Bellefonte, PA, USA) and the following multistep binary elution gradient, based on H_2_O as Phase A and ACN as Phase B, both containing 0.1% (*v/v*) FA, were adopted for the separation of FSs: 0–40 min, linear increase of B from 90% to 100%; 40–50 min, isocratic at 100% B; 50–52 min, linear decrease of B from 100% to 90%; 52–60 min, reconditioning at 90% B. Flow rate was set at 0.250 mL/min, and column temperature was kept at 30°C; 5 μL sample volumes were injected. The parameters of the APCI interface and of the ion optics of the Q‐Exactive spectrometer were set as follows: sheath gas flow rate, 40 a.u.; auxiliary gas flow rate, 10 a.u.; capillary temperature, 300°C; S‐lens RF level, 55 a.u.; vaporizer temperature, 250°C. Full scan MS acquisitions in positive ion mode were obtained in a 250–500 *m/z* interval, working with a 140 000 resolving power at *m/z* 200; the automatic gain control (AGC) level was set at 1 × 10^6^, and the maximum injection time was 100 ms. HCD‐FTMS/MS acquisitions were performed at a 35 000 resolving power in a 50–500 *m/z* interval, selecting a 1.0 *m/z* isolation window for precursor ions and using normalized collisional energy (NCE) at a value of 30 units; the AGC level was set at 2 × 10^5^, and maximum injection time was 100 ms. The spectrometer was calibrated every 2 days by infusing, at a 30 μL/min flow rate, calibration solutions for positive or negative polarity acquisitions provided by the instrument manufacturer, thus obtaining a mass accuracy always better than 5 ppm.

APCI‐direct infusion analyses (APCI‐DIAs) of individual standards solutions (30 μg/mL) were performed using both the Q‐Exactive spectrometer, under the same conditions described before, and a Velos Pro mass spectrometer, equipped with a two‐stage linear ion trap (Thermo Fisher, West Palm Beach, CA). Specifically, APCI‐DIA‐HCD‐FTMS/MS spectra were collected for specific sterols (*vide infra*) in the former case, whereas APCI‐DIA‐CID‐MS^n^ (*n* = 2, 4) experiments were performed with the linear ion trap instrument in order to elucidate major fragmentation patterns of all available FSs. The main operating parameters of the Velos Pro APCI interface and ion optics were set as follows: sheath gas flow rate, 40 a.u.; auxiliary gas flow rate, 10 a.u.; capillary temperature, 300°C; S‐lens RF level, 60 a.u.; vaporizer temperature, 250°C. A 1.0 *m/z* isolation window for the precursor ion and a NCE of 40 units for MS/MS and 25 units for MS^3^/MS^4^ were adopted; the AGC level was set at 2 × 10^3^, and the maximum injection time was 100 ms.

## 3 Results and Discussion

### 3.1 RPLC‐APCI(+)‐FTMS Analysis of Standard Sterols

**FIGURE 2:**
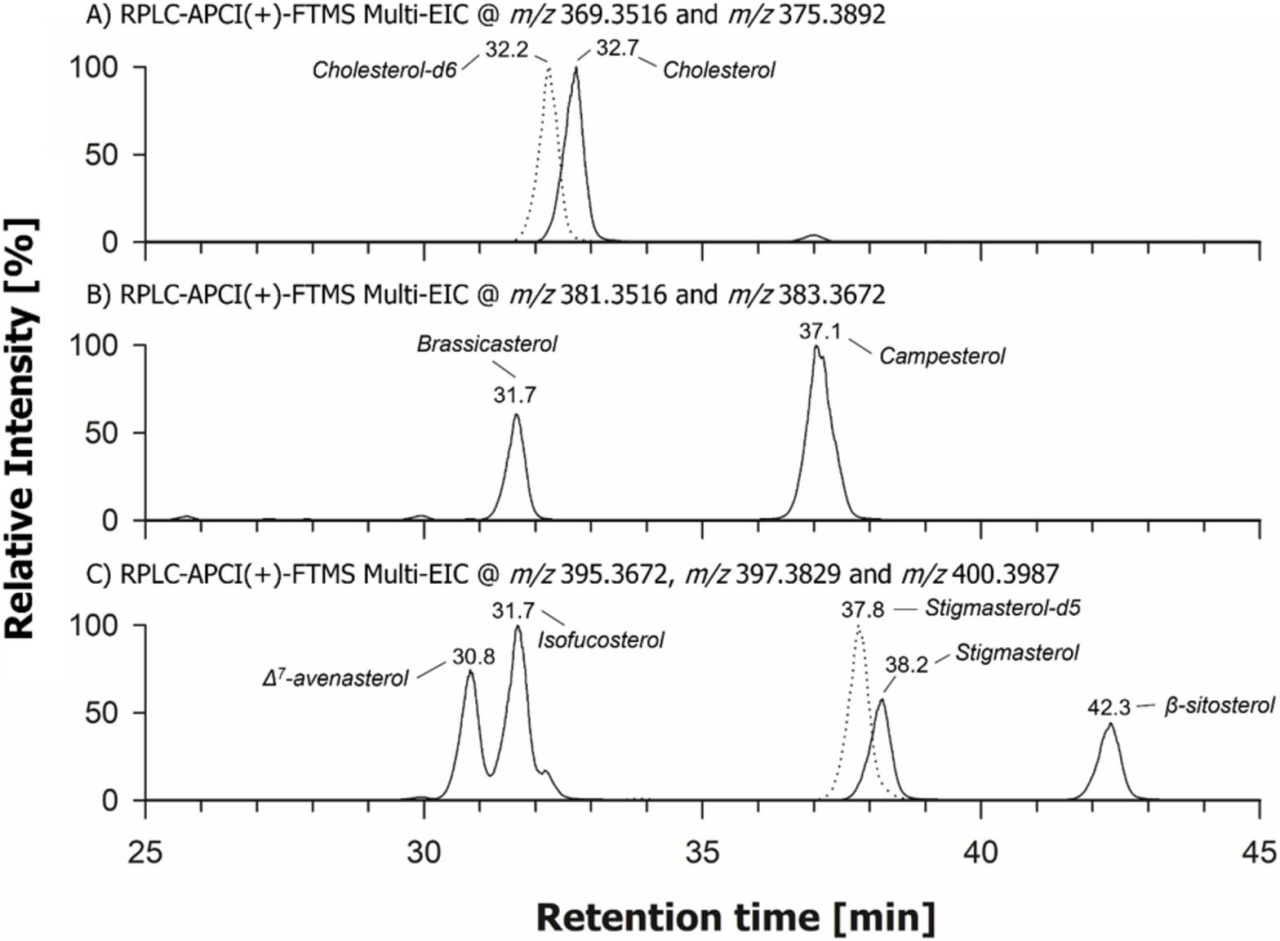
Detailed views of multiple extracted ion current (EIC) chromatograms referred to standard FSs considered in the present study, grouped according to their side chain (see Figure 1). (A) Cholesterol and its hexa‐deuterated analogue, that are two FSs not alkylated on C24: (B) 24‐methyl‐FSs and (C) 24‐ethyl‐ and 24‐ethylidene‐FSs. The following exact *m/z* values, referred to monoisotopic [M + H‐H_2_O]^+^ ions, were selected for ion current extraction: cholesterol, 369.3516; cholesterol‐d_6_, 375.3892; brassicasterol, 381.3516; campesterol, 383.3672; stigmasterol, Δ^7^‐avenasterol and isofucosterol (Δ^5^‐avenasterol), 395.3672; β‐sitosterol, 397.3829; and penta‐deuterated stigmasterol, 400.3987.

As a first step of the present study, RPLC‐APCI(+)‐FTMS was carried out on a mixture of standard sterols to explore the relationship between structure and retention time. Firstly, an isocratic separation based on 100% ACN containing 0.1% FA was attempted. However, a complete overlap was observed for peaks of positional isomers isofucosterol (Δ^5^‐avenasterol) and Δ^7^‐avenasterol, which share the same SC (see Figure 1). After several tests based on gradient elution, with H_2_O added to the mobile phase as the second solvent, suitable chromatographic conditions were identified, as detailed in the experimental section, allowing an effective separation of the two avenasterols. Figure 2 shows multiple extracted ion current (EIC) chromatograms finally obtained for monoisotopic *m/z* ratios referred to the [M + H‐H_2_O]^+^ ions generated by APCI for standard sterols, grouped according to the characteristics of their SC: (A) not alkylated on C24, (B) 24‐methyl and (C) 24‐ethylidene and 24‐ethyl.

The effect on the retention of deuteration of the two methyl groups linked to C25 in CHO‐d_6_ and of the ethyl group linked to C24 in stigmasterol‐d_5_ can be easily inferred from Figure 2, since a decrease (0.5/0.4 min) in retention time with respect to the corresponding nondeuterated analogues was observed (see Figure 2A,C, respectively). On the other hand, Figure 2 highlights the key role played by the sterol SC in the interaction with the octadecylic stationary phase.

Sterols with the same number and position of double bonds on the B ring but different SCs showed a clear pattern in retention. Based on their C24 substituent, those with a saturated SC were retained in the order: H < methyl < ethyl, corresponding to CHO < campesterol < β‐sitosterol (see Figure 2). A similar trend was observed for sterols including a double bond at C22–C23, with brassicasterol (methylated on C24) being less retained than stigmasterol (ethylated on C24). The occurrence of a C=C bond in the SC reduced the retention time compared to species with a saturated SC, as clearly inferred from the comparison between retention times of stigmasterol and β‐sitosterol in Figure 2C. This effect, consistent with that observed for fatty acids including one or more C=C bonds, when comparing their retention with the one of saturated fatty acids (see, e.g., Ref. [49]), varied depending on the C=C bond's location, being more relevant when it was closer to the end of the chain. Indeed, Δ^7^‐avenasterol and isofucosterol, having a C=C bond between C24 and C24′, exhibited a more relevant reduction in retention time (over 10 min) with respect to β‐sitosterol than stigmasterol (4 min), whose C=C bond is located between C22 and C23 (see Figure 2). The effect on retention exerted by the location of the C=C bond on Ring B was more subtle. Indeed, Δ^7^‐avenasterol was less retained than its positional isomer isofucosterol (Δ^5^‐avenasterol), suggesting that the closer proximity to the SC of the C=C bond between C7 and C8 reduced its interaction with the stationary phase. It is worth noting that an inversion in the respective retention times of the two isomeric avenasterols was observed by Jiang et al. using a C18 column and an isocratic elution based on ACN/methanol 99:1 (*v/v*), whereas the retention order of campesterol and β‐sitosterol was in accordance with the one observed during the present study [30].

The chromatographic behaviour described so far suggests that relative retention might be one of the tools for the recognition of new isomeric sterols in complex matrices, especially to infer the characteristics of their SC, by comparison with standards of known sterols. As shown in the following section, a careful interpretation of fragmentation pathways obtained by HCD‐FTMS/MS and CID‐MS^n^ for standard sterols, including those deuterated on appropriate positions of the molecular structure, provided valuable additional information, potentially useful for the identification of new PSs.

### 3.2 Fragmentation of Δ^5^‐Sterols With Saturated and Unbranched SC: CHO and Hexa‐Deuterated CHO

**FIGURE 3:**
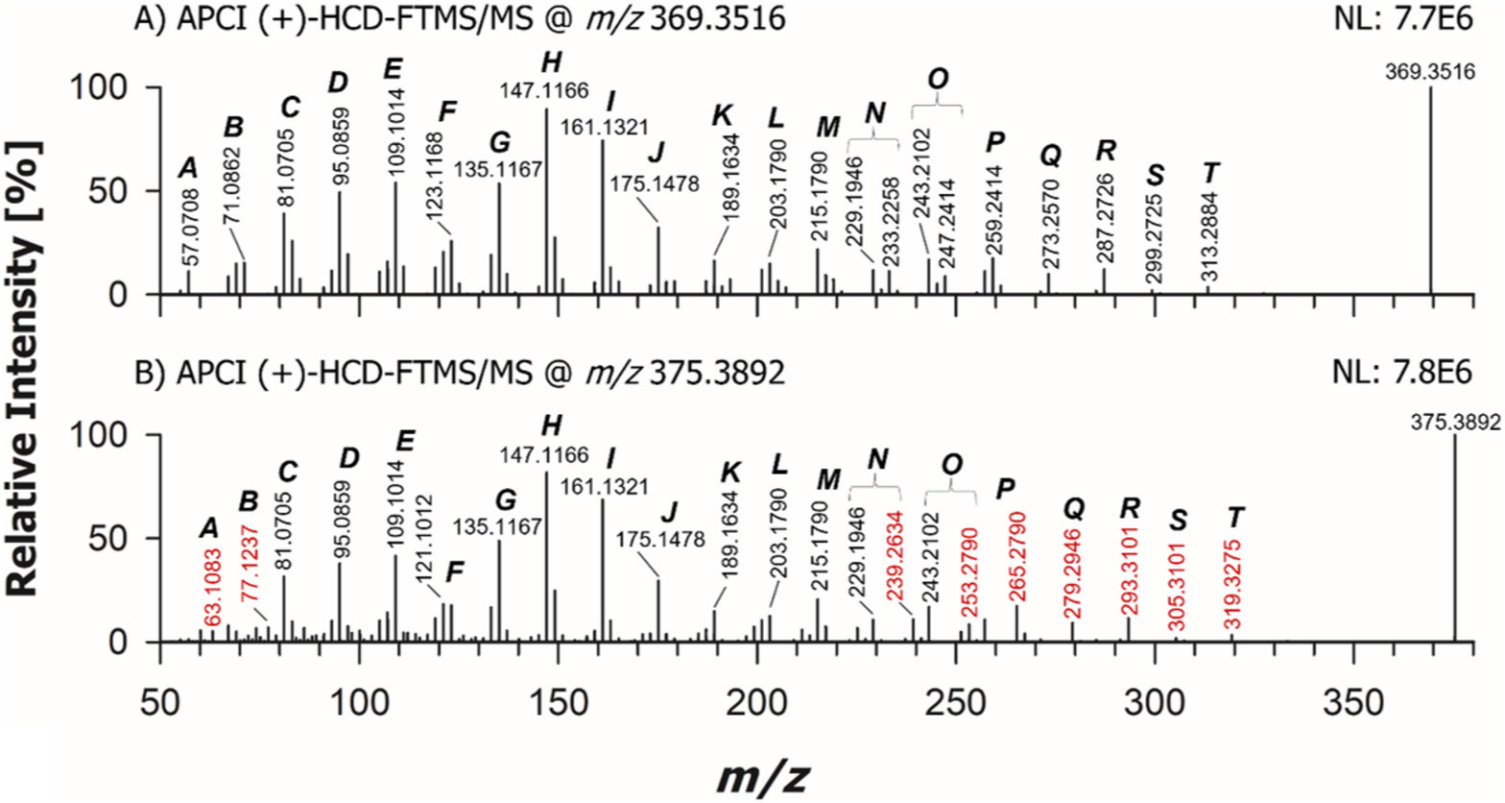
APCI(+)‐HCD‐FTMS/MS spectra referred to [M + H‐H_2_O]^+^ ions of cholesterol (Plot A) and cholesterol completely deuterated on methyl groups linked to C24 (cholesterol‐d_6_, Plot B), having exact *m/z* 369.3516 and 375.3892, respectively. Ions sharing the same number of C atoms but differing for the number of H and/or D atoms were grouped within a single cluster, indicated by a capital letter. The MS/MS spectrum of cholesterol‐d_6_ *m/z* values related to ions including six D atoms were reported in red. The NL numbers represent normalization levels (measured as counts/s) related to each spectrum. See text for details.

**FIGURE 4:**
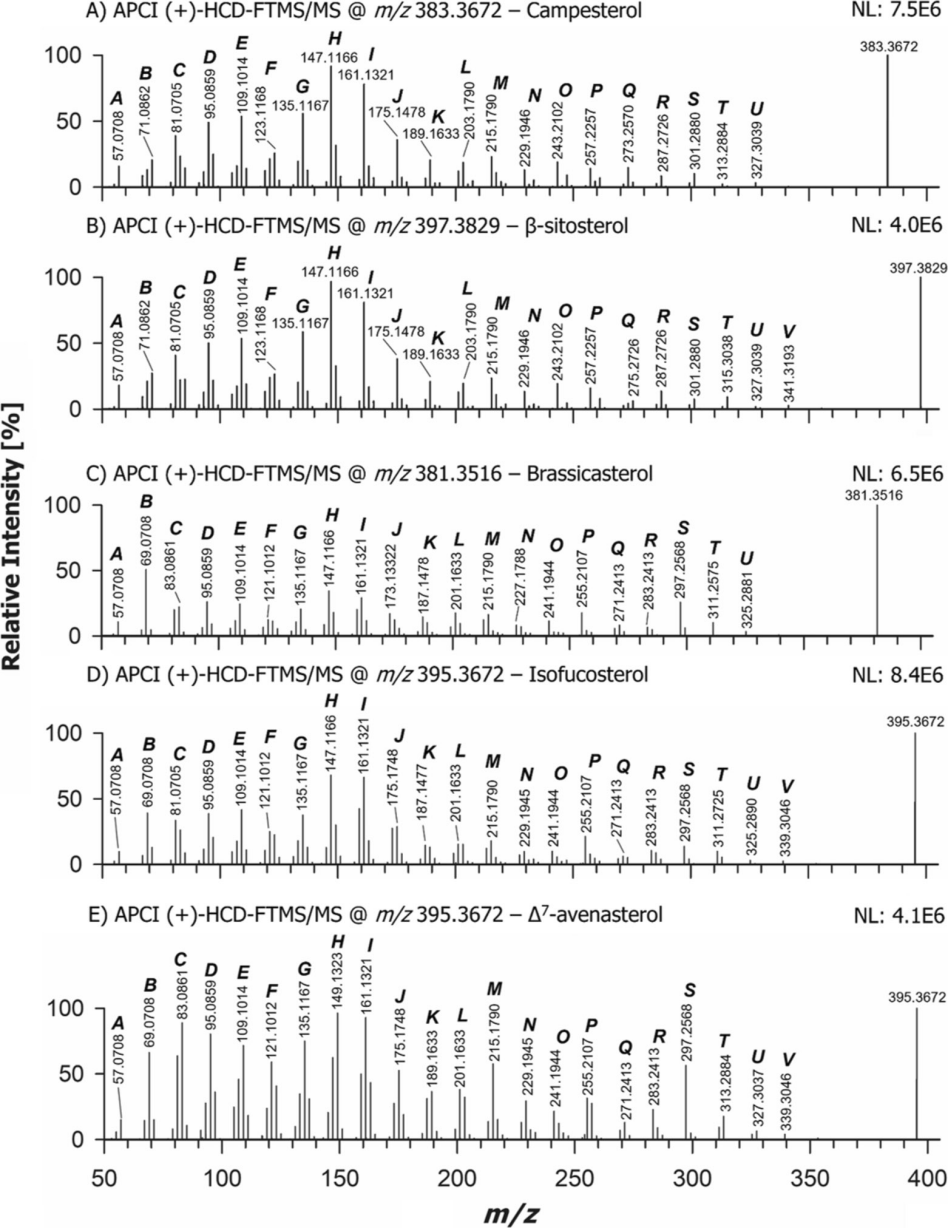
APCI(+)‐HCD‐FTMS/MS spectra acquired for the [M + H‐H_2_O]^+^ ions of the standard sterols considered during the present study, along with cholesterol and stigmasterol. Peak signals referred to product ions sharing the same number of carbon atoms and differing in the number of H atoms were grouped within a single cluster, identified by a capital letter. The experimental *m/z* ratio obtained for the most abundant signal in each cluster is reported. The NL numbers represent normalization levels (measured as counts/s) related to each spectrum.

To understand the general fragmentation pattern of Δ^5^‐sterols with a fully saturated SC, a comparison was made between CHO and its analogue deuterated on the two methyl groups linked to C25 (abbreviated as CHO‐d_6_ in the following). The APCI (+)‐HCD‐FTMS/MS spectra acquired for the respective [M + H‐H_2_O]^+^ ions (having exact *m/z* values: 369.3516 and 375.3892) are shown in Figure 3. For the sake of comparison, the APCI (+)‐HCD‐FTMS/MS spectra obtained for all the other sterols considered in the present study, excepting stigmasterol and its penta‐deuterated counterpart (*vide infra*), are grouped in Figure 4. The complex fragmentation pattern typically observed in tandem MS spectra of FSs reported in previous studies [30, 48, 50, 51, 52], leading to a characteristic distribution of product ion abundances, can be easily recognized in all spectra included in the two figures. Notably, a similar pattern has been systematically observed over more than four decades in sterol spectra obtained using electron ionization with high electron energy (70 eV) [53]. The comparison between spectra shown in Figures 3 and 4 reveals that sterol fragmentation produces apparently similar clusters of peaks, yet subtle variations in their relative abundances can be recognized by a deeper evaluation. To explore the correlation between structural features and specific abundance profiles, a systematic interpretation of those patterns based also on CID‐MS^n^ was undertaken in this study, including MS^3^ analyses (like in the study by Jiang et al. [30]) and, for the first time, also MS^4^ acquisitions.

To facilitate the description of spectral features, peak signals related to product ions having the same number of carbon atoms but differing in the number of H or D atoms were grouped within a single cluster, identified by a capital letter. Moreover, *m/z* values referred to ions including D atoms were highlighted in red in Figure 3B. Experimental *m/z* values rounded off to the fourth decimal place, molecular formulas inferred from them considering a maximum accuracy of 5 ppm and relative intensities for peak signals detected in each cluster for CHO and CHO‐d_6_ are reported in Table S1 of the Supporting Information. Minor product ions, not labelled in Figure 3 for the sake of clarity, are also included in the table.

**SCHEME 1:**
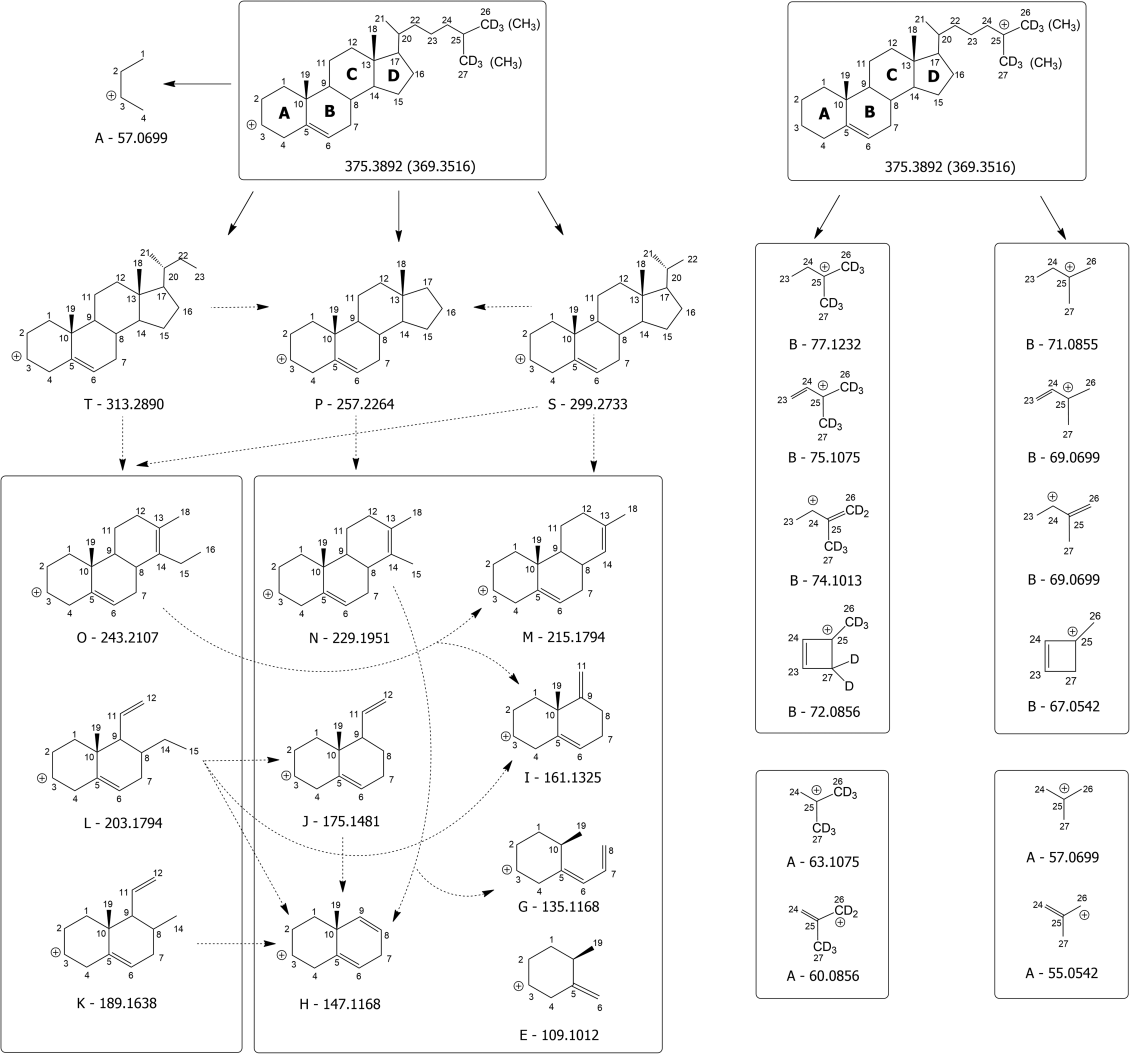
Fragmentation pathways hypothesized for cholesterol and cholesterol hexa‐deuterated on methyl groups linked to C25 considering their [M + H‐H_2_O]^+^ ions generated in the APCI source, with positive charge located (left side) on C3 (where the charge is originally located); (right side) on one of the terminal carbon atoms of the side chain. Plain arrows indicate fragmentations observed in MS/MS spectra (under both HCD and CID conditions); dashed arrows indicate fragmentations observed in CID‐MS^3^/MS^4^ spectra. Boxes include multiple product ions observed in a single MS^n^ (*n* = 2–4) spectrum. Exact *m/z* ratios (rounded off to the fourth decimal place) are reported for all product ions.

As emphasized by the red labels in Figure 3B, a 6‐unit shift in nominal *m/z* values was observed for several product ions when the precursor ion of CHO‐d_6_ was fragmented. This shift, found in Clusters A, B, N, O, P, Q, R, S and T, indicates the presence of the deuterated part of the SC in the corresponding structure. A systematic comparison across clusters provided further insights into this finding. Two extreme cases were identified: peak signals whose nominal *m/z* values were univocally displaced by 6 units (see bold *m/z* values in Table S1) and those which did not change at all their *m/z* values (apart from fluctuations on the fourth decimal digit, due to instrumental precision, occurring in *m/z* values underlined in Table S1) when CHO‐d_6_ was fragmented. These results indicated which product ions included the SC and which did not. For the remaining product ions, parallel mechanisms had to be considered. In these cases, both the *m/z* value already observed for nondeuterated CHO and one or more new ones, suggesting the presence of 5 or 6 D atoms in the ion structure, were recognized in the FTMS/MS spectrum of CHO‐d_6_. For example, the product ion detected at *m/z* 57.0708 in the MS/MS spectrum of CHO (Cluster A), consistent with a molecular formula C_4_H_9_^+^ (exact *m/z* 57.0699), was observed as a very weak signal also in the MS/MS spectrum of CHO‐d_6_ (see Table S1), but it was accompanied by a relatively stronger signal at *m/z* 63.1083, compatible with the formula C_4_H_3_D_6_^+^ (exact *m/z* 63.1075). As shown on the right side of Scheme 1, this formula can be related to the structure of a hexa‐deuterated *tert*‐butyl carbocation, arising from a dissociation occurring on the SC of CHO‐d_6_. The corresponding ion for CHO is a conventional *tert*‐butyl carbocation, having an exact *m/z* 57.0699, whose structure is also reported in Scheme 1.

The generation of these product ions provided a very important and, to the best of our knowledge, unprecedented evidence of the presence of multiple possible locations of positive charge on the structure of the [M + H‐H_2_O]^+^ precursor ions, alternative to C3, which is usually proposed as the unique location of positive charge for those ions (see, e.g., Refs. 30, 48). As illustrated in Scheme 1, the presence of positive charge on C25, leading to a tertiary carbocation, is required to explain the generation of the ion with exact *m/z* 63.1075 when CHO‐d_6_ is fragmented. By analogy, this feature also accounts for the generation of ions with exact *m/z* 71.0855 and 77.1232, when CHO and CHO‐d_6_ are fragmented, respectively. A detailed representation of the processes hypothesized to explain the generation of these ions and of all the other major product ions with *m/z* lower than 100 detected upon fragmentation of CHO and CHO‐d_6_ is provided in Figure S1 of the Supporting Information. In this case, as in most fragmentation process hypothesized to explain the generation of sterol product ions (*vide infra*), the proposed mechanism was a 1,3‐H migration between two carbon atoms, with a bond breakage occurring between the second and the third atom involved in the migration and the generation of a C=C bond between the first and the second atom, in accordance with a mechanism proposed in the literature for the charge remote fragmentation of long‐chain functionalized alkanes and alkenes [54].

Interestingly, as indicated in the upper‐left part of Scheme 1, a product ion with exact *m/z* 57.0699 might be generated also by CHO‐d_6_ if the positive charge was located on the typical C3 position, since it would result from the fragmentation of Ring A. However, as shown in the fragmentation reaction reported at the top of Figure S1, this process would likely imply the release of a very strained neutral fragment. This might explain the appearance of a very weak peak signal at a *m/z* ratio of 57.0708, consistent with the exact value of 57.0699, in the MS/MS spectrum of CHO‐d_6_ (see Table S1).

Notably, other product ions detected in Clusters A and B for CHO and CHO‐d_6_ could only be explained by considering additional possible locations of the positive charge near the end of the CHO SC, specifically at C24 and C26 atoms (see the right side of Scheme 1 and Figure S1), in addition to C25. The most plausible, though somewhat unusual, process leading to the displacement of the positive charge on positions like C24, C25 and C26 is a hydride transfer occurring from the carbon atom representing the new location of the positive charge to C3, where the charge was initially located after the in‐source loss of H_2_O from the protonated form of CHO. Notably, Bao et al. [55] previously proposed an intramolecular hydride transfer occurring upon CID in the case of cyclopentenone oxylipins. On the other hand, hydride abstraction was reported to occur from hydrocarbon chains during positive ion APCI, leading to carbocations [56]. If occurring on sterols, this process would lead to [M‐H]^+^ ions or, in the case of subsequent in‐source H_2_O loss, to [M‐H‐H_2_O]^+^ ions, yet none of them showed a significant intensity in APCI(+)‐FTMS spectra of sterols in the present case, thus strengthening the hypothesis that the protonation of the OH group, followed by H_2_O loss and generation of a carbocation on C3, was the prevailing ionization process occurring in the APCI(+) source.

A systematic interpretation of the spectral profiles displayed in Figure 3 reveals that at least one other possible location of positive charge on the structure of CHO and CHO‐d_6_ [M + H‐H_2_O]^+^ ions needs to be hypothesized to explain major product ions detected in MS/MS spectra. A key approach during this step of the investigation, expanding the use of MS^3^ already reported for some sterols by Jiang et al. [30], involved the acquisition of the MS^3^ spectrum for each major product ion detected in HCD‐FTMS/MS spectra shown in Figure 3 and of MS^4^ spectra for selected product ions detected in MS^3^ spectra. These MS^3^ and MS^4^ spectra were obtained under low energy CID conditions using a linear ion trap mass spectrometer, after verifying that each product ion detected in HCD‐MS/MS spectra was also present in low energy CID‐MS/MS ones. A set of genealogical trees, describing schematically the information inferred from MS^3^ and MS^4^ acquisitions performed during the present study on sterols, is included in Figure S2 of the Supporting Information.

As a result, solid arrows in Scheme 1 indicate transitions directly observed at the MS/MS stage, while dashed ones represent selected transitions observed at either the MS^3^ or the MS^4^ stage that led to product ions detected also in MS/MS spectra (the complete representation of all transitions experimentally observed is available in Figure S2). Additionally, boxes reported in the schemes include all major product ions detected directly in an MS/MS (if preceded by a solid arrow) or MS^3^ (if preceded by a dashed arrow) spectrum, with the precursor being the ion preceding a specific box. For example, the product ions with exact *m/z* values of 229.1951 and 215.1794 (belonging to Clusters N and M, respectively) shown in a box on the left side of Scheme 1 were detected in the 375 > 257 > MS^3^ spectrum of CHO‐d_6_ and also in the 369 > 257 > MS^3^ spectrum of CHO (see also the respective genealogical trees in Figure S2). In this case, the 375 > 313 > 243 > MS^4^ spectrum clarified that the *m/z* 215.1794 ion could be generated from the 243 one (as indicated by the curved dashed arrow connecting the two ions in Scheme 1), whereas the *m/z* 229.1951 ion was directly generated from the precursor ion with exact *m/z* 257.2264 (see the genealogical trees for CHO‐d_6_ in Figure S2). The remarkable amount of spectral information obtained through the adopted multistage MS approach required a complex and careful interpretation, yet it provided an unprecedented level of detail for CHO fragmentation, representing a crucial foundation for explaining the spectral differences observed for other sterols under study, considering their structural differences.

As shown on the left side of Scheme 1, the typical location hypothesized for the positive charge in the CHO [M + H‐H_2_O]^+^ ion, that is, C3 (where the original OH group is linked), was considered a starting point for the interpretation of fragmentation pathways. This approach successfully explained the formation of major product ions in Clusters T (exact *m/z* 313.2890) and S (exact *m/z* 299.2739) and a minor ion in Cluster P (exact *m/z* 257.2264) through dissociations involving the CHO SC, with the complete detachment of the deuterated portion, if present. A description of the specific processes hypothesized to explain the generation of those ions is provided in Figure S3 of the Supporting Information. However, these mechanisms could not account for the generation of product ions with *m/z* values systematically shifted by 6 units in the case of CHO‐d_6_ (see Figure 3B), thus suggesting that further pathways had to be considered (*vide infra*). Notably, Scheme 1 reports plausible product ions originating from product ions T, S and P, all with the positive charge located on C3 and detected in MS^3^/MS^4^ spectra. The corresponding fragmentation processes, described in detail in Figure S3, involve the sequential opening and fragmentation of Rings D, C and B of CHO, without the displacement of the original C5–C6 double bond. As emphasized in Figure S3, the proposed processes often involve the already mentioned 1,3‐H migration with a C‐C bond breakage and a C=C bond formation. Moreover, the subsequent generation of different structures of the same ion has often to be invoked to finally account for the formation of a specific product ion. Notably, the structure proposed for the product ion consistent with an exact *m/z* 147.1168 (Cluster H), which is the base peak in the HCD‐FTMS/MS spectra of CHO (see Figure 3), matches exactly with the ones proposed by Jiang et al. in the fragmentation scheme for stigmasterol [30] and by Münger et al. [48], who identified that ion as a general diagnostic product ion for Δ^5^ sterols. The structure shown for that ion in Scheme 1 and Figure S3 is also consistent with the detection of the most abundant product ion at nominal *m/z* 149 reported by Igarashi et al. [52] when fragmenting CHO including two carbon‐13 (^13^C) atoms at C3 and C4. An excellent agreement was also found with the results reported by Trösken et al. [50] and Mo et al. [57] for the structure proposed in Scheme 1 and Figure S3 for another relevant product ion, the I one corresponding to an exact *m/z* 161.1325. Indeed, this structure corresponds to that of the product ion detected at nominal *m/z* 167 by those authors when fragmenting CHO deuterated twice on C2 and C4 and once on C3 and C6 [50, 57]. Notably, Trösken *et al*. detected further relevant product ions at nominal *m/z* 81 and 95 for CHO deuterated on those carbon atoms [50]. However, no plausible mechanism could be found to produce ions with those *m/z* ratios assuming that the positive charge was on C3. This failure, along with the total lack of explanation for hexa‐deuterated forms of ions T and S, and for major deuterated and nondeuterated ions belonging to Clusters O, P, Q and R observed in the present study, strongly suggested that additional mechanisms, likely implying different charge locations with respect to those reported in Scheme 1, had to be hypothesized.

**SCHEME 2:**
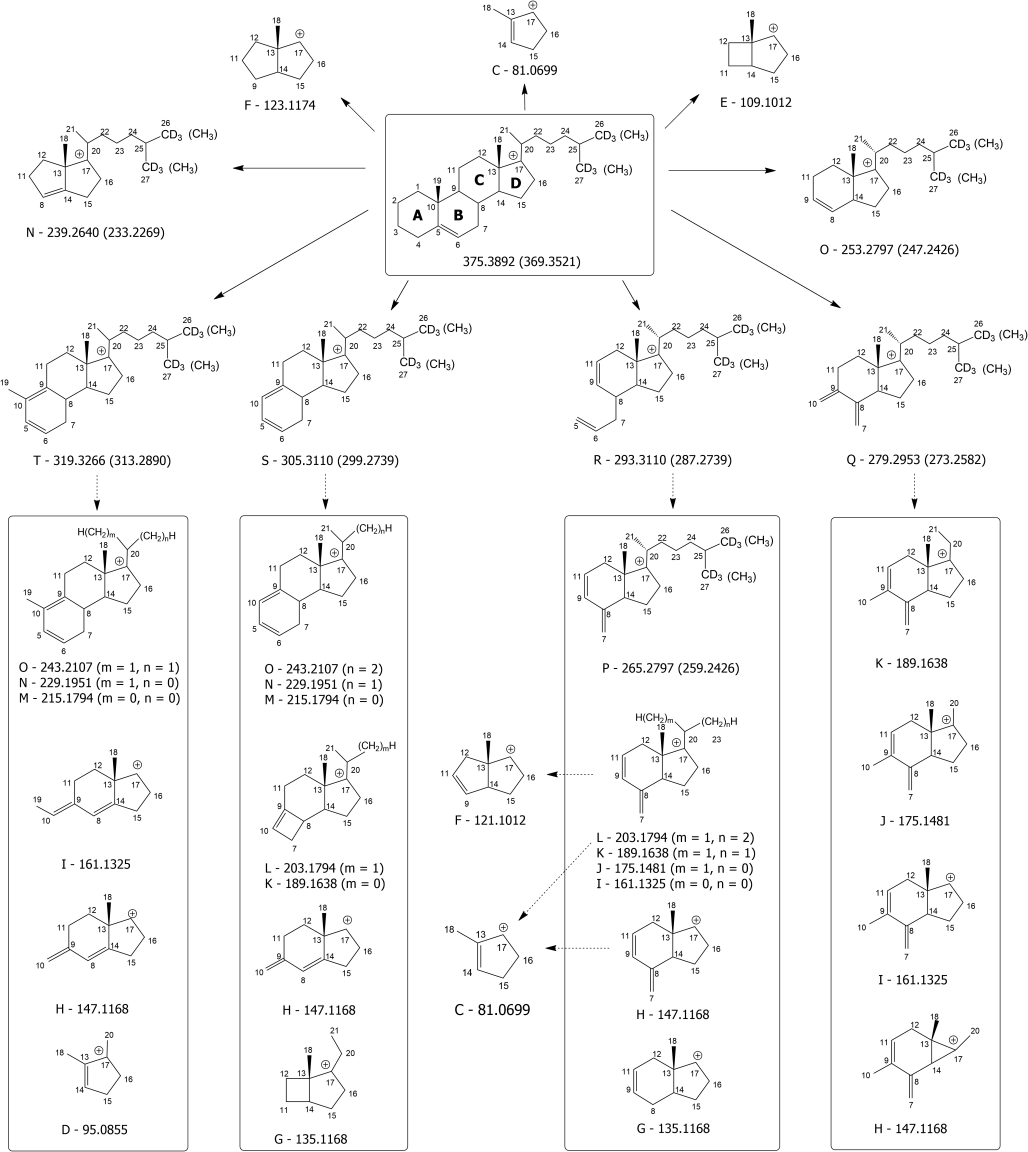
Fragmentation pathways hypothesized for cholesterol and cholesterol hexa‐deuterated on methyl groups linked to C25 considering their [M + H‐H_2_O]^+^ ions generated in the APCI source, with positive charge located on C17. Plain arrows indicate fragmentations observed in MS/MS spectra (under both HCD and CID conditions); dashed arrows indicate fragmentations observed in CID‐MS^3^/MS^4^ spectra. Boxes include multiple product ions observed in a single MS^n^ (*n* = 3, 4) spectrum. Exact *m/z* ratios (rounded off to the fourth decimal place) are reported for all product ions.

The key for the individuation of a new charge location was provided by the ion detected at *m/z* 81.0705 for both CHO and CHO‐d_6_ (see Figure 3), thus certainly not including the terminal part of the SC. A plausible mechanism leading to a product ion with an exact *m/z* ratio (81.0699) consistent with that value (within a 5 ppm accuracy) involves the generation of an allylic carbocation with the pentacyclic structure shown at the top and the bottom of Scheme 2. This process might involve a retrocycloaddition occurring at Ring C, previously proposed in the literature [51], followed by the complete elimination of the SC mediated by a 1,3‐H transfer from C22 to C17 (see Figure S4). The mechanism suggests the location of positive charge on C17, a favourable position, since it corresponds to a tertiary carbocation, likely originated (like carbocations on the SC commented before) through an intramolecular hydride transfer, in this case occurring from C17 to the original location of positive charge, C3. As depicted in Scheme 2, the positive charge on C17 enables a complete interpretation of the CHO and CHO‐d_6_ fragmentation patterns, explaining the entire set of product ions labelled in the HCD‐FTMS/MS spectra reported in Figure 3. A detailed representation of fragmentation steps leading to product ions reported in Scheme 2 is provided in Figures S5, S6 and S7 of the Supporting Information.

Structures for Ions T, S, R, Q, P and O, compatible with the 6 units shift in nominal *m/z* ratio observed for them when CHO‐d_6_ was fragmented, could be found by considering the progressive fragmentation of Rings A and B. As emphasized by one of the dashed arrows in Scheme 2 and in Figure S2, the *m/z* 265.2797 ion, belonging to Cluster P of deuterated CHO, was detected in the MS^3^ spectrum of the *m/z* 293.3110 ion of Cluster R, thus facilitating its structural interpretation. Similarly, a complete set of product ions, enclosed in rectangular boxes in Scheme 2, was observed in the respective MS^3^ spectra of Ions T, S, R and Q reported in the scheme. The MS^4^ spectra further highlighted the complex relationships existing between couples of product ions detected in those MS^3^ spectra. For the sake of clarity, dashed arrows were not included inside those boxes in the case of Scheme 2, but the respective transitions were carefully analysed to validate each structure shown in the scheme and are evidenced in the genealogical trees in Figure S2. Notably, a 28 Da neutral loss corresponding to the detachment of an ethylene molecule was very common, whereas a 14 Da neutral loss, putatively corresponding to a methylene loss, was rarely observed, although appearing consistent with the frequent detection of 14 *m/z* units‐spaced ions in MS^3^ spectra (see Figure S2). For instance, the *m/z* 215.1794 ion (Cluster M) was never found in the fragmentation spectrum of the *m/z* 229.1951 one (Cluster N) but exhibited a relevant signal in the spectrum of the precursor at *m/z* 243.2107 (Cluster O). On the other hand, methylene losses were inevitably hypothesized to occur from ions with exact *m/z* 161.1325 and 135.1168 since they were inferred from the corresponding MS^3^ spectra (see the second genealogical tree for the CHO‐d_6_ precursor ion in Figure S2 and the fragmentation shown at the top‐right side of Figure S6). The relationships found both upwards and downwards in the fragmentation cascades depicted in Schemes 1 and 2 and in Figure S2 were very useful to formulate consistent structural hypotheses for each product ion, usually considering ring openings and H intramolecular transfers as the possible mechanisms.

As shown in Scheme 2, the progressive fragmentation of the SC of Ions T, S, R and Q, with the immediate loss of the deuterated portion in the case of CHO‐d_6_, was critical to explain the 6‐unit shift in *m/z* ratios observed only for ions of Clusters T to P in the MS/MS spectra reported in Figure 3. Notably, among product ions generated in the case of CHO‐d_6_, those with exact *m/z* 239.2640 and 253.2797, belonging to Clusters N and O, respectively, were not detected in any of the MS^3^ or MS^4^ spectra discussed so far (see the genealogical trees for CHO‐d_6_ in Figure S2). This absence suggested that they were generated directly from the [M + H‐H_2_O]^+^ precursor ion of CHO‐d_6_ and still included the six D atoms. Consequently, their formation likely implied the fragmentation or the complete loss of Rings A and B, and possibly part of Ring C, but did not involve the SC, as depicted in the upper part of Scheme 2 and in the respective fragmentation processes in Figure S5. The corresponding nondeuterated ions, having exact *m/z* values of 233.2269 and 247.2426, respectively, were formed and detected in the HCD‐FTMS/MS spectrum when the [M + H‐H_2_O]^+^ ion of CHO was fragmented. As highlighted in Scheme 2, all peak signals in the other clusters displayed identical *m/z* ratios for CHO and CHO‐d_6_, excepting those in Clusters A and B, for the reasons previously explained (see Scheme 1).

The location of the positive charge on C17 was also important to explain the generation of product ions with a relatively low *m/z* (< 150) among them. Indeed, the ion with exact *m/z* 123.1174 (corresponding to the major peak signal of Cluster F, see Figure 3) was absent in all CHO‐related MS^3^ spectra concerning Ions T, S, R and Q, but it was detected in the MS^3^ spectrum of the *m/z* 257.2264 ion (a minor ion of Cluster P, not labelled in Figure 3). Therefore, it was assumed to be formed from the latter, as described in the top‐right side of Figure S5. As shown in Scheme 2 and in Figure S5, the corresponding structure was compatible with the retainment of Ring D and part of Ring C, and the positive charge on C17 was a determining feature for its generation. This was also the case for the structure shown in the upper part of Scheme 2 for the *m/z* 109.1012 ion (Cluster E), that can be considered complementary to the one proposed in Scheme 1. The positive charge on C17 was also important to provide an interpretation for product ions with *m/z* 121.1012 and 81.0699, detected in Clusters F and C, respectively. As shown in Scheme 2 and Figure S2, these ions were detected both in 375 > 203 > and in 375 > 147 > MS^3^ spectra and in 375 > 293 > 203 > and 375 > 293 > 147 > MS^4^ spectra; thus, their direct precursors were ions with *m/z* ratios of 203.1794 (Cluster L) and 147.1168 (Cluster H), and possible mechanisms for the respective generation from those precursors are reported in Figures S6 and S7. This finding supported the hypothesized chemical structures for the two ions and confirmed the structure previously hypothesized for the *m/z* 81.0699 ion. It is worth noting that the mechanism previously hypothesized for the *m/z* 81.0699 ion, reported in Figure S4, remains plausible, as the intermediate product ion of the process, that would have a *m/z* 199.2327 for CHO‐d_6_ and 193.1956 for CHO (see Figure S1), was detected in the respective HCD‐FTMS/MS spectra, although with very low intensity (they were not labelled with *m/z* ratios in Figure 3). This consideration can be extended to other product ions as well. As exemplified in Figure S8, the *m/z* 215.1794 ion of Cluster M might be explained also with a cycloelimination on Ring D occurring directly on the [M + H‐H_2_O]^+^ ions of both CHO and CHO‐d_6_, assuming the typical positive charge location on C3. Although the final structure would be the same reported in Scheme 1, this pathway would not explain the relationship with the *m/z* 243.2107 ion, inferred from MS^3^ and MS^4^ spectra (see Figure S2). The potential occurrence of multiple pathways leading to certain product ions can be thus considered a typical feature of the complex fragmentation processes involving CHO and other sterols.

As discussed in detail in the following sections, the careful interpretation of these complex fragmentation pathways allowed for the explanation of subtle differences in the fragmentation profiles observed for typical plant sterols with slightly different structures, paving the way for identifying general structure–fragmentation relationships.

### 3.3 Fragmentation of Δ^5^‐Sterols With a Methyl or Ethyl Group at C24: Campesterol and β‐Sitosterol

As evidenced in Figure 1, campesterol and β‐sitosterol, two of the most significant PSs, are closely related to CHO in terms of molecular structure, since they include the same steroidal backbone (with a C=C bond between C5 and C6) and a completely saturated SC. The only structural difference lies in the presence of a methyl or ethyl group attached to C24, respectively. Therefore, the general similarity observed for APCI(+)‐HCD‐FTMS/MS spectra of CHO, campesterol and β‐sitosterol (see Figures 3 and 4) is not surprising. Specifically, the profiles of Ion Clusters D through M were nearly identical for the three sterols, with the *m/z* 147.1166 ion (Cluster H, exact *m/z* 147.1168) consistently appearing as the base peak in the MS/MS spectrum. This similarity is reasonable only if all product ions related to those clusters do not include the SC, as proposed in Schemes 1 and 2.

**SCHEME 3:**
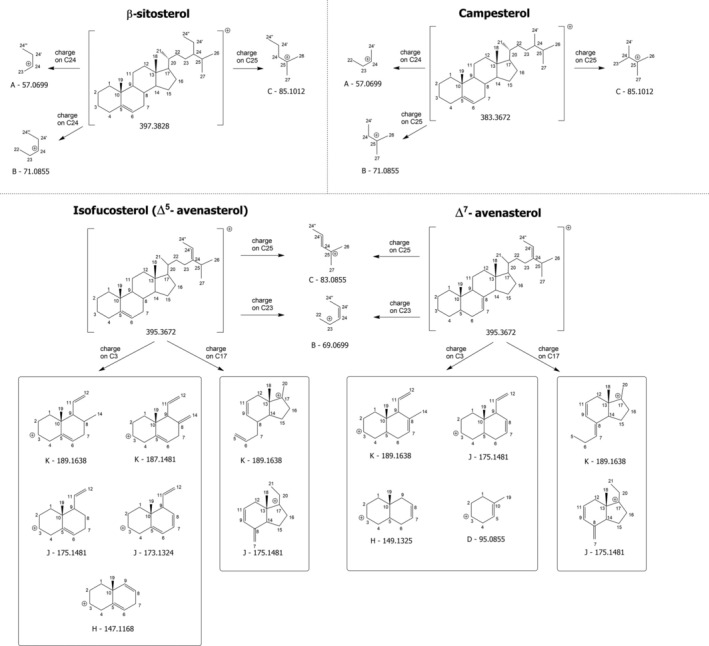
Structures hypothesized for product ions detected in the HCD‐FTMS/MS spectra of the [M + H‐H_2_O]^+^ ions of β‐sitosterol, campesterol, isofucosterol (Δ^5^‐avenasterol) and Δ^7^‐avenasterol and recognized as relevant in the assessment of specific structural features of the four phytosterols. The location of the positive charge required to explain the final structures is indicated for each (group of) product ion(s). See text for details.

However, subtle but significant differences were recognized in other clusters. For instance, an increase was observed for the ion of Cluster C with nominal *m/z* 85 in the case of β‐sitosterol (it was not labelled in Figure 4 for clarity). As depicted in Scheme 3 and in Figure S9, this finding aligns with the generation of a tertiary carbocation, similar to the one at *m/z* 57.0699 depicted on the right side of Scheme 1 but modified by the presence of the ethyl moiety linked to C24 (exact *m/z* 85.1012).

Scheme 3 further illustrates that when a methyl or ethyl group is attached to C24, the latter becomes another stable location for the positive charge on the SC, like C25, as it corresponds to a tertiary carbocation. Accordingly, pathways leading to product ions with exact *m/z* 57.0699, 71.0855 and 85.1012 can be easily hypothesized both for campesterol and β‐sitosterol by placing the positive charge on C24 or C25 (see Figure S9). As for product ions with a high *m/z* ratio, a significant difference in relative abundance was found for the ion of cluster N detected at *m/z* 233.2258 for CHO (see Figure 3), which was nearly undetectable for campesterol and β‐sitosterol (see Figure 4). This finding further supports the structure hypothesized for that ion in Scheme 2. Indeed, the methylation/ethylation of C24 would lead to a 14/28‐unit shift in the nominal *m/z* ratio of this product ion in the case of campesterol and β‐sitosterol, explaining why signals at nominal *m/z* 247 (O) and 261 (P) were observed in the corresponding MS/MS spectra (see Figure 4A,B). To maintain clarity, these two signals were not labelled with their *m/z* ratios in the figure. A similar reasoning applies to the *m/z* 247 ion (O) of CHO, which also includes the SC (see Scheme 2) and was thus shifted to nominal *m/z* values of 261 for campesterol (Cluster P, not labelled in Figure 4A) and 275 in the case of β‐sitosterol (Cluster Q, see Figure 4B). Despite exhibiting a low intensity, the latter signal can be considered a diagnostic product ion of β‐sitosterol among Δ^5^‐sterols with a saturated SC.

Another remarkable difference between the two sterols and CHO was found in Cluster P, where the *m/z* 257 ion prevailed over the *m/z* 259 one, thus reversing the abundance ratio observed for CHO (see Figures 3 and 4A,B). As described in Scheme 1 and Figure S3, the *m/z* 257 ion (exact value 257.2264) might result from the complete elimination of the SC from the CHO precursor ion having its charge on C3 through a 1,3 H migration already proposed by Münger *et al*. [48]. An alternative mechanism, illustrated in Figure S10, might explain why the resulting product ion was more abundant in the case of campesterol and β‐sitosterol. Specifically, a concerted process [51], involving a six‐membered transition state with a 1,5‐H transfer from C24 to C17 could be hypothesized. As shown in Figure S10, this process would lead to the detachment of a propene molecule and of an alkene with double substitution (by an isopropyl group and by a methyl or ethyl group) on one of the carbon atoms involved in the C=C bond (C24) rather than the single substitution observed on that atom in the case of CHO.

As for other differences observed between CHO and campesterol/β‐sitosterol, the counterparts of CHO product ions belonging to Clusters P, Q, R, S and T shown in Scheme 2 were detected in Clusters Q/R, R/S, S/T, T/U and U/V, respectively, for campesterol/β‐sitosterol (see Figure 4A,B), in accordance with the 14/28 Da shift occurring on the SC of these sterols. Notably, as shown by the genealogical trees reported for CHO, campesterol and β‐sitosterol in Figure S2, the MS^3^ spectra obtained for the corresponding ions detected in the respective MS/MS spectra displayed the same set of product ions, thus emphasizing the similarity between them, apart from the SC. These findings indirectly supported the structural hypotheses made in Scheme 2 for CHO Product Ions P, Q, R, S and T, which, as discussed before, are also consistent with the results obtained for CHO‐d_6_.

The comparison between MS/MS spectra of CHO, campesterol and β‐sitosterol highlights the critical role played by the positive charge location on C17 in the fragmentation pathways of the respective [M + H‐H_2_O]^+^ ions. It suggests that product ions including the entire SC, having *m/z* ratio values close to that of the precursor ion, may enable the distinction between the three sterols. Additionally, an increase in product ions detected at nominal *m/z* 85 (Cluster C) and 275 (Cluster Q) was observed for β‐sitosterol.

### 3.4 Fragmentation of Δ^5^‐Sterols With a Double Bond at C22‐C23: Stigmasterol/Penta‐Deuterated Stigmasterol and Brassicasterol

**FIGURE 5:**
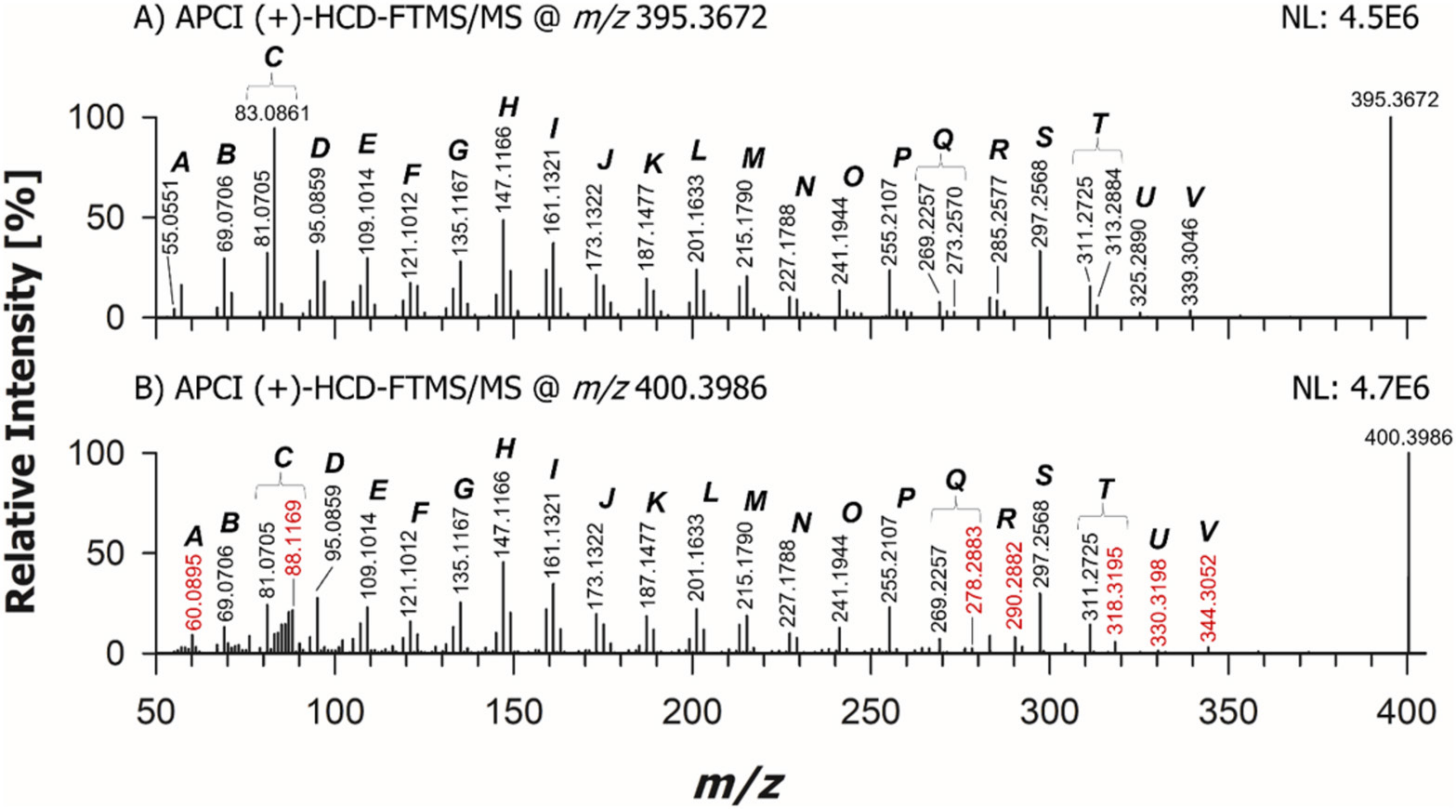
APCI(+)‐HCD‐FTMS/MS spectra referred to [M + H‐H_2_O]^+^ ions of stigmasterol (Plot A) and stigmasterol completely deuterated on the ethyl group linked to C24 (stigmasterol‐d_5_, Plot B), having exact *m/z* values 395.3672 and 400.3986, respectively. Ions sharing the same number of C atoms but differing in the number of H and/or D atoms were grouped within a single cluster, indicated by a capital letter. The MS/MS spectrum of stigmasterol‐d_5_ (Plot B) *m/z* values related to ions including D atoms were reported in red. See text for details. The NL numbers represent normalization levels (measured as counts/s) related to each spectrum.

Both stigmasterol and brassicasterol, which are two further significant plant sterols, possess a C=C bond between C22 and C23 along their SC (see Figure 1). This feature is expected to influence their fragmentation patterns, thus potentially leading to differences with respect to sterols discussed so far in this study. By analogy with CHO, isotopically labelled stigmasterol including five D atoms on the ethyl group linked to C24 was analysed during this study to recognize product ions including the terminal part of the SC. The APCI(+)‐FTMS/MS spectra of [M + H‐H_2_O]^+^ ions for stigmasterol and stigmasterol‐d_5_ (having exact *m/z* values 395.3672 and 400.3986) are compared in Figure 5. Product ion clusters are labelled as in previous spectra, with *m/z* ratios referred to deuterated product ions reported in red. A detailed list of experimental *m/z* values, molecular formulas inferred from them and relative abundances for all product ions detected in the two spectra is provided in Table S2 of the Supporting Information using the same code adopted in the case of Table S1 to mark *m/z* ratios.

Comparing Figures 3 and 5 clearly reveals that product ions in clusters from D to I had the same *m/z* ratios for both CHO and stigmasterol, as well as their deuterated derivatives. This finding supports the structures proposed in Schemes 1 and/or 2 for those ions, as none of them involve the SC, where CHO and stigmasterol differ from each other and each of them differs from its isotopically labelled counterpart. Conversely, differences between CHO and stigmasterol were observed for Clusters A, B and C, thus confirming that they are directly related to the terminal part of the SC (see Scheme 1). As illustrated in Scheme S1, which compares the structures proposed for relevant product ions of stigmasterol and stigmasterol‐d_5_, and in Figure S11, where pathways hypothesized for their formation are reported, most product ions in those clusters can be explained by considering the positive charge on C24. Indeed, this position corresponds to a carbocation that is both tertiary and allylic in the case of stigmasterol. Interestingly, the same structure could be hypothesized for the *m/z* 69.0699 ion in the case of CHO and stigmasterol (see Schemes 1 and S1). In fact, this ion results from the loss of the ethyl chain linked to C24 (see Figure S11), where D atoms are located, and which is absent in the case of CHO. On the other hand, the *m/z* 83.0855 ion observed for stigmasterol can be explained by three possible structures, all corresponding to stable allylic (in one case also tertiary) carbocations. This interpretation may account for its role as the base peak in the HCD‐FTMS/MS spectrum of stigmasterol (see Figure 5A) and highlights this ion as a distinctive marker for the presence of a C=C bond between C22 and C23 and of the ethyl moiety linked to C24. As evidenced in Scheme S1 and Figure S11, one of the structures proposed for the *m/z* 83.0855 ion was confirmed by the detection of its penta‐deuterated counterpart at *m/z* 88.1169, while the other two were supported by the detection of a tetra‐deuterated product ion at *m/z* 87.1106 and a tris‐deuterated product ion at *m/z* 86.1044 in the MS/MS spectrum of stigmasterol‐d_5_ (see Figure 5B, where the peak signals of these ions, in Cluster C, were not labelled with their *m/z* ratios for the sake of clarity).

It is worth noting that two previous studies reported the detection of a *m/z* 83 ion in EI‐MS [58] or MS/MS spectra [59] of stigmasterol, both suggesting structures lacking the ethyl moiety linked to C24.

However, the analysis of stigmasterol deuterated on that group suggests that a different pathway likely generates the ion, at least under conditions related to the so‐called HCD mode. Moreover, as inferred from Figure 5B and Table S2, a non‐negligible peak signal was detected for stigmasterol‐d_5_ at a *m/z* ratio consistent with the exact value 85.0950 (see Table S2), indicating the generation of a further product ion including four D atoms. As illustrated at the bottom of Figure S11, the structure proposed for this ion would imply the original transfer of a deuteride (instead of a hydride) from C24′ to the original location of positive charge (C3), resulting in a positive charge on C24′. In principle, a further structure for the *m/z* 83.0855 ion (not reported in Scheme S1) would be possible for nondeuterated stigmasterol, according to the same pathway.

As mentioned before, comparing CHO and stigmasterol MS/MS spectra (see Figures 3 and 5) revealed significant differences in detected *m/z* ratios beginning from Cluster J and extending to those with higher *m/z* ratios. These differences can mostly be explained considering product ions previously described for CHO in Scheme 2. Indeed, T, S, R and P ions and the *m/z* 247 ion of Cluster O described in Scheme 2, all including the entire SC, correspond, respectively, to the single ions detected in Clusters V and U and to the minor ions in Clusters T, R and Q (those having nominal *m/z* values 313, 285 and 273) in the case of stigmasterol. The shifts of +26 units in nominal *m/z* ratios are due to the presence of the ethyl group on C24 and the C=C bond between C22 and C23 (see Scheme S1). As expected, the penta‐deuterated counterparts of those five ions were detected in the MS/MS spectrum of stigmasterol‐d_5_ (see red labelled *m/z* ratios in Figure 4B and the related structures in Scheme S1). As emphasized in Scheme S1, the positive charge of these ions is likely located on C24, as this is both a tertiary and an allylic carbon in the case of stigmasterol. However, charge remote fragmentations proposed to explain all the five product ions in the case of CHO (with the positive charge located on C17), involving Rings A and/or B of the steroidal backbone, were confirmed (see Schemes 2 and S1). A detailed representation of fragmentation processes hypothesized to explain the structures of stigmasterol and stigmasterol‐d_5_ product ions reported in Scheme S1 and having *m/z* > 100 and the positive charge on C24 or C17 is provided, respectively, in Figures S12 and S13 of the Supporting Information.

Remarkably, none of the other relevant peak signals detected in clusters from J to T in the case of stigmasterol showed the 5‐unit shift related to the deuteration of the ethyl group in the case of stigmasterol‐d_5_, indicating the absence of that moiety in their structure. Among them, the *m/z* 311 ion of Cluster T and the *m/z* 241 ion of Cluster O represented the stigmasterol counterparts of the *m/z* 313 and 243 ions proposed for CHO in Schemes 1 and 2, respectively, considering the presence of a C=C bond between C22 and C23 (see Scheme S1). Conversely, structures identical to those proposed for ions with nominal *m/z* 95, 109, 135, 147, 161 and 215 in the case of CHO could be hypothesized for stigmasterol (see Schemes 1, 2 and S1), as the portion of SC differing between the two molecules was absent in their structure.

A more complex evaluation was needed for the remaining product ions detected in the stigmasterol MS/MS spectrum and not showing the expected +5‐unit shift when stigmasterol‐d_5_ was fragmented. Firstly, 395 > 297 > and 400 > 297 > CID‐MS^3^ spectra were acquired by fragmenting the *m/z* 297 ion (Cluster S) originated by both stigmasterol and stigmasterol‐d_5_ [M + H‐H_2_O]^+^ ions. As evidenced in the genealogical trees reported for the *m/z* 400.4 ion and for the *m/z* 395.4 ion of stigmasterol in Figure S2, both spectra revealed the same features, that is, a series of signals with 14‐units spaced nominal *m/z* values from 269 to 135. As proposed in Figure S14, the *m/z* 297 ion (exact value 297.2577) might be generated through a two‐step gas phase process. The first step would involve a cycloelimination, enabled by the C=C bond between C22 and C23 in the stigmasterol SC, with loss of propene; this would be followed by the loss of but‐1‐ene, eventually penta‐deuterated. Additionally, if the positive charge was initially located on C17, as shown in Figure S14, a very stable tertiary/allylic carbocation would finally be formed, thus explaining the significant abundance of the *m/z* 297 ion in the MS/MS spectrum of stigmasterol and of its deuterated counterpart (see Figure 5). Nonetheless, the same mechanism might occur considering the positive charge on C3, as shown in Figure S15, and the resulting ion, indicated as 297.2577 A in Scheme S1, would coincide with the *m/z* 297 ion proposed by Münger et al. [48] as a typical feature of fragmentation for sterols with a C=C bond between C22 and C23, although a different mechanism was hypothesized by those authors. In the present study, the 395 > 297 > and 400 > 297 > CID‐MS^3^ spectra clarified that the entire set of stigmasterol product ions mentioned earlier was preferentially generated by the *m/z* 297 ion, as emphasized in the right side of Scheme S1. The detailed fragmentation processes hypothesized for those ions, shown in Figure S15, suggested that even a further structure (297.2577 B in Scheme S1) had to be considered for the *m/z* 297 ion with the positive charge on C3 to enable an easier interpretation of all the detected product ions. Notably, the *m/z* 255 ion (Cluster P), indicated by Münger et al. [48] as a characteristic product ion arising directly from the [M + H‐H_2_O]^+^ ions of sterols having a C=C bond between C22 and C23 and another one on the steroidal backbone, was included among them.

In the case of stigmasterol, a general agreement between structures hypothesized for product ions could be made, at least for the nondeuterated sterol, between the present study and the one by Jiang et al. [30], in which the quadrupole‐Orbitrap mass spectrometer was also used. One of the relevant differences was the absence, in the fragmentation scheme proposed by those authors, of product ions still keeping the SC (i.e., ion of clusters from Q to V reported on the right side of Scheme S1), although peak signals potentially corresponding to some of them seemed to be present in the FTMS/MS spectrum reported in Ref. [30]. On the other hand, product ions with *m/z* < 100 were not considered by the authors since 100 was the lowest *m/z* value explored in their spectrum. As discussed so far in this paper, those low *m/z* product ions can be very important to clarify the structural characteristics of the SC of sterols; thus, they should be included in the acquired MS/MS spectra of sterols, if possible.

Following stigmasterol, brassicasterol, that is, another Δ^5^‐sterol including a C=C bond between C22 and C23 and differing from stigmasterol only for the presence of a methyl group, instead of an ethyl one, linked to C24, was examined. This structural similarity is evident in the APCI(+)‐HCD‐FTMS/MS spectra of the respective [M + H‐H_2_O]^+^ ions, reported in Figures 4C and 5A, at least for product ions that did not include the SC portion where the structural difference occurs, namely, all those corresponding to clusters from D to P and the *m/z* 297 one (Cluster S, see Scheme S1). Among product ions with higher *m/z* ratios, brassicasterol ions with nominal *m/z* values 325 (U), 311 (T) and 271 (Q) correspond, respectively, to Ions V, U and R detected for stigmasterol (see Scheme S1).

The most notable differences between brassicasterol and stigmasterol spectra were observed for lower *m/z* product ions, particularly those of Clusters B and C. Indeed, the prevailing product ion observed at nominal *m/z* 83 for stigmasterol (see Scheme S1) became the *m/z* 69 ion in the case of brassicasterol due to the replacement of the ethyl group with methyl at C24. Accordingly, the *m/z* 69 ion was the most abundant product ion detected for brassicasterol, making it a diagnostic feature to recognize the presence of a methyl group linked to C24 in conjunction with a double bond between C22 and C23.

### 3.5 Fragmentation of Δ^5^‐Sterols and Δ^7^‐Sterols With a Double Bond at C24–24′: Isofucosterol (Δ^5^‐Avenasterol) and Δ^7^‐Avenasterol

As illustrated in Figure 1, isofucosterol (Δ^5^‐avenasterol) and Δ^7^‐avenasterol are isomeric sterols sharing an ethylidene group linked to C24 and differing only for the position of the double bond on Ring B. These compounds were thus studied to evaluate if the different position of that C=C bond might influence sterol fragmentation pathways. The APCI(+)‐HCD‐FTMS/MS spectra for the respective [M + H‐H_2_O]^+^ ions (exact *m/z* 395.3672) are reported in Figure 4D,E. It is important to note that the presence of a C=C bond between carbon atoms C24 and C24′ may favour the localization of positive charge on C25, C23 and C24″, as these would all be allylic carbocations. As shown in Scheme 3 and Figures S16 and S17, the positive charge on C23 (corresponding to an allylic/secondary carbocation) and on C25 (an allylic/tertiary carbocation) enables an easy interpretation of product ions detected, respectively, at *m/z* 69.0708 and 83.0861 (consistent with exact values 69.0699 and 83.0855) for the two sterols (see Figure 4D,E), when considering SC dissociations. However, due to the significantly higher abundance of the *m/z* 83.0861 ion in the case of Δ^7^‐avenasterol, an alternative interpretation was searched for. As a result, the process shown in Figure S18 was proposed. Specifically, it involves the preliminary breakage of the C10–C9 bond, mediated by a 1,3 H transfer from C11 to C10 (and the formation of a C=C bond between C9 and C11), followed by the detachment from Ring A of a pentacyclic secondary carbocation with an exact *m/z* 83.0855 and the release of a neutral molecule with an extended C=C bond conjugation. As inferred from Figure S18, this process requires the absence of a C=C bond between C5 and C6, thus explaining why it would be favoured in the case of Δ^7^‐avenasterol. For the same reason, the process is expected to be hindered also in the case of stigmasterol, thus explaining why the *m/z* 83 peak signal was very low in the case of stigmasterol‐d_5_ (see Figure 5B). In this case, the specific structure of the *m/z* 83 ion proposed for stigmasterol was formed but including the deuterated portion of the molecule, thus leading to a set of product ions with nominal *m/z* values ranging from 85 to 88 (see the upper‐right side of Scheme S1).

Excepting a higher abundance of the *m/z* 95 ion in Cluster D for Δ^7^‐avenasterol, due to the possibility of direct generation from Ring A (see the structure shown in Scheme 3 and the fragmentation reported in Figure S17), a similar internal abundance distribution was observed for the product ions of clusters from D to G and Cluster I in the case of isofucosterol and Δ^7^‐avenasterol, with a good accordance also with the corresponding clusters observed for stigmasterol (see Figures 4 and 5A). This result is reasonable, as the three sterols share the same molecular formula (C_29_H_48_O), and aligns with the structures proposed for ions of those clusters in Schemes 1 and 2, referred to CHO but also valid for stigmasterol. The shift of the C=C bond from C5–C6 to C7–C8 would still make those structures consistent with the experimental *m/z* ratios. However, this is not the case of the structure proposed for the important product ion with exact *m/z* 147.1168 (Cluster H) discussed before. As evidenced in Scheme 3 and Figure S17, the presence of a C=C bond between C7 and C8 would favour the generation of a product ion with exact *m/z* 149.1325 in the case of Δ^7^‐avenasterol, through a different mechanism involving a bond breakage between Rings A and B and Ring C. This hypothesis was experimentally confirmed, as the prevailing peak signal for Cluster H was detected at *m/z* 149.1323 in the case of Δ^7^‐avenasterol (see Figure 4), making it a diagnostic product ion for the C=C bond location between C7 and C8.

Characteristic differences in relative abundances were observed for further signal clusters when comparing isofucosterol, Δ^7^‐avenasterol and stigmasterol, starting from Cluster J. In the case of isofucosterol and Δ^7^‐avenasterol, a signal with an *m/z* ratio compatible with the exact value 175.1481 was predominant, whereas a signal compatible with the exact *m/z* value 173.1324 showed the highest abundance for stigmasterol. As shown in Scheme 3, the structure proposed in Scheme 1 for the *m/z* 175 ion, with the positive charge located on C3, would be valid for both isofucosterol and Δ^7^‐avenasterol. In contrast, the structure having an exact *m/z* ratio of 173.1324 proposed for stigmasterol in Scheme S1 would necessarily require the presence of a C=C bond between C5 and C6. A higher abundance can thus be predicted for this product ion also in the case of isofucosterol, compared to Δ^7^‐avenasterol, although it does not become more abundant than the *m/z* 175.1481 ion. This hypothesis was confirmed by the MS/MS spectra of the two compounds (see Figure 4). It is worth noting that an alternative structure was proposed for the *m/z* 175 ion in the case of CHO, with the positive charge located on C17 and involving the breakage of the C20–C22 bond (see Scheme 2). This structure is essential to explain the characteristic abundance of this product ion observed for sterols lacking a C=C bond between C22 and C23, namely, CHO, campesterol, β‐sitosterol, isofucosterol and Δ^7^‐avenasterol (see Figures 3 and 4), and its low abundance for sterols including that C=C bond, namely, stigmasterol and brassicasterol (see Figures 4C and 5). The breakage of the C20–C22 bond leading to the *m/z* 175 ion with positive charge on C17 requires a 1,3 H transfer from C23 to C20. This process is significantly more difficult if C23 is involved in a C=C bond, as in stigmasterol and brassicasterol.

While comparing further product ions obtained for isofucosterol and Δ^7^‐avenasterol, an interesting inversion in abundance was observed for ions with nominal *m/z* values of 187 and 189 in Cluster K. As illustrated in Scheme 3, this finding can be explained by the easier generation of the *m/z* 187 ion in the case of isofucosterol since the C5–C6 double bond is already present and an additional C=C bond is formed between C8 and C14 during fragmentation (see Figure S16). Similar internal patterns were generally observed for clusters from L to V for the two species (see Figure 4), the only differences being an increased abundance of the *m/z* 215, 257 and 297 ions in the case of Δ^7^‐avenasterol. This finding aligns with the structures reported for ions in those clusters in Schemes 1, 2 and S1, as the position of the C=C bond on Ring B is not expected to affect their validity. On the other hand, the increased abundance of the three specific ions cited before suggests that the C=C bond between C7 and C8 can positively influence their generation due to the different molecular conformation induced by that position. More generally, this conformational effect might also explain the higher relative abundance of product ions with respect to the residual precursor ion observed for Δ^7^‐avenasterol compared to isofucosterol (see Figure 4).

### 3.6 Correlation Between Specific Product Ions and Structural Features of 4‐Desmethyl Sterols

The interpretation of fragmentation spectra for 4‐desmethyl sterols discussed in the previous sections revealed subtle differences despite their general similarity. These differences might help in identifying the occurrence of specific structural features. This idea is highlighted by the comparison of relative intensities typically observed for all product ions related to the sterols considered during the present study, reported in Table S3 of the Supporting Information.

Table 1 summarizes the most informative product ions, linking specific structural features to intensity relationships or percent intensities, based on the fragmentation spectra. This table might be employed to assess structural features like the position of the double bond on Ring B, the presence of a double bond on the SC (either between C22 and C23 or on the branch linked to C24) and the occurrence of an alkyl group linked to C24 by carefully considering the intensity ratios existing between peak signals in specific clusters or, in some cases, the relative intensity in the entire spectrum.

**TABLE 1:** Summary of correlations between specific structural features and intensity relationships for the most structurally informative product ions of 4‐desmethyl‐sterols, grouped according to clusters shown in Figures 3, 4 and 5.

Cluster	Exact *m/z* value(s)	Intensity relationships or percent intensities	Structural features
B	69.0699	69.0699 > > 71.0855	Δ^22(23)^, Δ^24(24′)^ and Δ^7^‐sterols
	71.0855	71.0855 > 69.0699	Δ^5^‐sterols with saturated SC, eventually alkylated at C24
C	81.0699	81.0699 > 83.0855	Δ^5^‐sterols with saturated SC, eventually alkylated at C24 and Δ^5,24(24′)^
	83.0855	> 80%	Δ^5,22(23)^ with ethyl group at C24 and Δ^7^‐sterols
	85.1012	~ 15%	Δ^5^‐sterols with saturated SC alkylated at C24
D	95.0855	> 80%	Δ^7^‐sterols
F	121.1012	121.1012 > 123.1168	Δ^22(23)^ or Δ^24(24′)^
	123.1168	123.1168 > 121.1012	Saturated SC, eventually alkylated at C24
H	147.1168	147.1168 > > 149.1325	Δ^5^‐sterols
	149.1325	149.1325 > 147.1168	Δ^7^‐sterols
I	161.1325	161.1325 > > 163.1481	Δ^5^‐sterols and Δ^7^‐sterols
	163.1481	~ 40%	Δ^7^‐sterols
J	173.1324	173.1324 > 175.1481	Δ^22(23)^
	175.1481	175.1481 ~ 173.1324	Δ^5,24(24′)^
		175.1481 > > 173.1324	Δ^5^‐sterols with saturated SC, eventually alkylated at C24 and Δ^7,24(24′)^
K	187.1481	187.1481 > 189.1638	Δ^22(23)^
	189.1638	189.1638 ≥ 187.1481	Δ^24(24′)^
		189.1638 > > 187.1481	Sterols with saturated SC, eventually alkylated at C24
L	201.1638	201.1638 ~ 203.1794	Δ^5,24(24′)^
		201.1638 > 203.1794	Δ^22(23)^ and Δ^7,24(24′)^
	203.1794	203.1794 > 201.1638	Sterols with saturated SC, eventually alkylated at C24
M	215.1794	> 60%	Δ^7^‐sterols
N	227.1794	227.1794 > 229.1946	Δ^22(23)^
	229.1946	229.1946 > 227.1794	Sterols with saturated SC, eventually alkylated at C24 and Δ^24(24′)^
O	241.1951	241.1951 > > 243.2107	Δ^22(23)^ and Δ^24(24′)^
	243.2107	243.2107 > > 241.1951	Sterols with saturated SC, eventually alkylated at C24
P	255.2103	255.2103 > 257.2269	Δ^22(23)^ or Δ^24(24′) 48^
	257.2269	~ 30%	Δ^7^‐sterols
		257.2269 > 259.2426	Δ^5^‐sterols with saturated SC and alkylated at C24 [48]
	259.2426	259.2426 > 257.2269	Δ^5^‐sterols with saturated and unbranched SC
R	283.2420	283.2420 > 287.2733	Δ^22(23)^ and Δ^24(24′)^
	287.2733	287.2733 > 283.2420	Sterols with saturated SC, eventually alkylated at C24
S	297.2577	297.2577 > > 299.2739	Δ^22(23)^ and Δ^24(24′) 48^
T	311.2733	311.2733 > 313.2890	Δ^5,22(23)^ and Δ^5,24(24′)^
	313.2890	313.2890 > 311.2733	Δ^5^‐sterols with saturated SC, unbranched or with methyl group at C24 and Δ^7^‐sterols
	315.3046	315.3046 > 313.2890	Δ^5^‐sterols with saturated SC and ethyl group at C24

*Note:* The correlations are based on HCD‐FTMS/MS and CID‐MS^n^ (*n* = 2–4) analyses of selected standards. Exact *m/z* values rounded to four decimal places are reported. Percentages correspond to relative intensities observed in the MS/MS spectrum.

Combining this information with the precursor ion *m/z* ratio and the differences in *m/z* ratios observed between its peak signal and nearby signals in the MS/MS spectrum, reflecting neutral losses occurring on the SC, might aid in identifying unknown plant sterols in vegetal samples.

## 4 Conclusions

The complex fragmentation patterns obtained for 4‐desmethyl‐sterols when subjected to positive ion MS/MS were thoroughly analysed using natural and isotopically labelled standard compounds. The study employed a complementary approach using accurate *m/z* values of MS/MS peak signals provided by a high‐resolution mass spectrometer, insights gained from multistage mass spectrometry (MS^3^–MS^4^) using a linear ion trap and comparisons of product ion *m/z* ratios between natural and isotopically labelled standards. The intricate nature of 4‐desmethyl‐sterols MS/MS spectra was found to arise from a combination of factors, the most relevant being the existence of multiple potential locations for the positive charge, which, to the best of our knowledge, was evidenced for the first time for sterols. This feature was likely caused by the occurrence of unique intramolecular hydride transfers that displaced the positive charge of the original carbocation formed during the APCI process upon protonation of the OH group and subsequent loss of a H_2_O molecule, thus located on the carbon atom conventionally numbered as C3. In particular, indirect evidence was obtained for an alternative location of positive charge on carbon atoms conventionally labelled as C17, on the steroidal backbone, and C24/C25, on the side chain, due to their stabilization as tertiary carbocations. As a result, multiple fragmentation pathways were proposed to account for all detected product ions, with some ions possibly having several structures consistent with their *m/z* ratios. The investigation highlighted the important role played by low (< 100) *m/z* product ions, mainly arising from the side chain, and by product ions generated from the sterol [M + H‐H_2_O]^+^ precursor ions through small neutral losses occurring from that chain, related to C‐C bond breakages. Despite they had not received a specific attention in previous studies, those ions proved very useful to infer the structural characteristics of the side chain (location of the C=C bond, nature of the substitution at C24). Indeed, significant correlations were found between the intensity ratios of selected product ions among them and the characteristics of the SC. Other diagnostic correlations between intensity ratios or percent intensities and structural features were found for product ions whose generation was influenced by the position of the C=C bond on Ring B.

These insights provide a foundation for identifying unknown sterols in complex samples, such as plant extracts, and might thus contribute to a deeper understanding of this important class of metabolites.

## Author Contributions

**V. Cinquepalmi:** investigation, data curation, methodology, visualization, writing – original draft, conceptualization. **I. Losito:** conceptualization, validation, supervision, resources, writing – review and editing, project administration. **A. Castellaneta:** investigation, methodology. **C.D. Calvano:** writing – review and editing. **T.R.I. Cataldi:** funding acquisition, resources, writing – review and editing.

## Supporting information


**Figure S1.** Mechanisms hypothesized to explain the generation of low *m/z* (< 100) product ions shown in Scheme 1 from the [M + H‐H_2_O]^+^ ions of cholesterol (CHO) and of cholesterol (CHO‐d_6_) completely deuterated on the methyl groups linked to C25. For the sake of clarity, the transfer of H, with concurrent breakage of a C‐C bond and the formation of a new C=C bond is depicted explicitly only in the first process drawn in the scheme. Nonetheless, the C‐C bond broken as a consequence of this process is highlighted in all fragmentation reactions using a transversal dashed line. Exact *m/z* ratios, rounded off to the fourth decimal place, are reported.
**Figure S2.** Genealogical trees constructed for sterol [M + H‐H_2_O]^+^ ions to describe the information inferred from HCD‐FTMS/MS and CID‐MS^3/^MS^4^ acquisitions performed, respectively, using a quadrupole‐Orbitrap and a linear ion trap mass spectrometer. The *m/z* ratio of the precursor ion involved in the HCD‐FTMS/MS acquisition is reported on top of each tree and is then connected to boxes representing the *m/z* ratios and percentual relative intensities observed in the HCD‐FTMS/MS spectrum, for the resulting product ions that were subsequently subjected to CID‐MS^3^ acquisitions (note that the same set of product ions was observed in HCD‐FTMS/MS and in CID‐MS/MS spectra of sterol [M + H‐H_2_O]^+^ ions). Each of those boxes is then connected to a series of boxes describing *m/z* ratios and relative intensities observed for ions detected in CID‐MS^3^ spectra. Among the latter, boxes with *m/z* ratios and relative intensities written in blue colour represent precursor ions selected for subsequent CID‐MS^4^ acquisitions. In this case, the set of *m/z* ratios of major peak signals detected in the MS^4^ spectrum was not explicitly indicated since it corresponded to the set inferred from the MS^3^ spectrum acquired on the same precursor ion and reported in one of the genealogical trees for the same sterol. Notes: (1) The *m/z* ratios of ions including D atoms in their structures are written in red character; (2) the letter adopted to mark each product ion cluster in FTMS/MS spectra has been reported also for all ions inside the trees; (3) for the sake of consistency, all *m/z* ratios have been rounded to the first decimal place, in accordance with the precision level available with the linear ion trap mass spectrometer. The figure follows in the next seven pages.
**Figure S3.** Mechanisms hypothesized to explain the generation of product ions with *m/z* > 100 shown in Scheme 1 from the [M + H‐H_2_O]^+^ precursors ions of cholesterol (CHO) and of cholesterol‐d_6_ (CHO‐d_6_). The C‐C bonds broken during fragmentation steps are highlighted using a transversal dashed line, eventually coloured to relate the bond breakage to a specific process. Exact *m/z* ratios, rounded off to the fourth decimal place, are reported.
**Figure S4.** Mechanism potentially responsible for the formation of the product ion detected at *m/z* 81.0705 directly from the [M + H‐H_2_O]^+^ ion of cholesterol and cholesterol‐d6. It involves a retrocycloaddition at the C‐ring and the removal of the side chain through a 1,3‐H transfer from C22 to C17, with breakage of the C17–C20 bond and formation of a C=C bond between C20 and C22. Exact *m/z* ratios, rounded off to the fourth decimal place, are reported.
**Figure S5.** Mechanisms hypothesized to explain the generation of product ions shown in the upper half of Scheme 2 from the [M + H‐H_2_O]^+^ precursors ions of cholesterol (CHO) and of cholesterol‐d_6_ (CHO‐d_6_). The C‐C bonds broken during fragmentation steps are highlighted using a transversal dashed line. Exact *m/z* ratios, rounded off to the fourth decimal place, are reported.
**Figure S6.** Mechanisms hypothesized to explain the generation of product ions related to ions with *m/z* 319.3266/313.2890 (upper part) and 305.3110/299.2739 (lower part) shown in Scheme 2, representing product ions of the [M + H‐H_2_O]^+^ ions of cholesterol (CHO) and of cholesterol‐d_6_ (CHO‐d_6_). The C‐C bonds broken during fragmentation steps are highlighted using a transversal dashed line. Exact *m/z* ratios, rounded off to the fourth decimal place, are reported.
**Figure S7.** Mechanisms hypothesized to explain the generation of product ions related to ions with *m/z* 293.3110/287.2739 (upper part) and 279.2953/273.2582 (lower part) shown in Scheme 2, representing product ions of the [M + H‐H_2_O]^+^ ions of cholesterol (CHO) and of cholesterol‐d_6_ (CHO‐d_6_). The C‐C bonds broken during fragmentation steps are highlighted using a transversal dashed line. Exact *m/z* ratios, rounded off to the fourth decimal place, are reported.
**Figure S8.** Mechanism hypothesized for the generation of the product ion at *m/z* 215.1790 directly from the [M + H‐H_2_O]^+^ ion of cholesterol or cholesterol‐d6. The displayed process is a cycloelimination on Ring D, leading also to the complete detachment of the side chain. Exact *m/z* ratios, rounded off to the fourth decimal place, are reported.
**Figure S9.** Mechanisms hypothesized to explain the generation of product ions with *m/z* < 100 from [M + H‐H_2_O]^+^ ions of β‐sitosterol and campesterol. The C‐C bonds broken during fragmentation steps are highlighted using a transversal dashed line. Exact *m/z* ratios, rounded off to the fourth decimal place, are reported.
**Figure S10.** Alternative mechanism proposed for the generation of the product ion detected at *m/z* 257.2257 in the APCI(+)‐HCD‐FTMS/MS spectra of the [M + H^+^‐H_2_O]^+^ ions of campesterol (R = CH_3_) and β‐sitosterol (R = CH_2_CH_3_). Exact *m/z* ratios, rounded off to the fourth decimal place, are reported.
**Figure S11.** Mechanisms hypothesized to explain the generation of low *m/z* (< 100) product ions shown in Scheme S1 from the [M + H‐H_2_O]^+^ precursor ions of stigmasterol and of stigmasterol completely deuterated on the ethyl group linked to C24. The C‐C bonds broken during fragmentation steps are highlighted using a transversal dashed line. Exact *m/z* ratios, rounded off to the fourth decimal place, are reported.
**Figure S12.** Mechanisms hypothesized to explain the generation from the [M + H‐H_2_O]^+^ precursor ions of stigmasterol and stigmasterol‐d_5_ of product ions with positive charge on C24 shown in Scheme S1 for Clusters Q to V. The C‐C bonds broken during fragmentation steps are highlighted using a transversal dashed line. Exact *m/z* ratios, rounded off to the fourth decimal place, are reported.
**Figure S13.** Mechanisms hypothesized to explain the generation from the [M + H‐H_2_O]^+^ precursor ions of stigmasterol and stigmasterol‐d_5_ of product ions with positive charge on C17 shown in Scheme S1. The C‐C bonds broken during fragmentation steps are highlighted using a transversal dashed line. Exact *m/z* ratios, rounded off to the fourth decimal place, are reported.
**Figure S14.** Fragmentation pathway proposed to explain the generation of a product ion with a *m/z* ratio consistent with the exact value 297.2577 upon fragmentation of the [M + H^+^‐H_2_O]^+^ ions of stigmasterol/stigmasterol‐d_5_. Note that the same pathway might be considered if the positive charge was located on C3 (see Figure S15). Exact *m/z* ratios, rounded off to the fourth decimal place, are reported.
**Figure S15.** Mechanisms hypothesized to explain the generation from the [M + H‐H_2_O]^+^ precursor ions of stigmasterol and stigmasterol‐d_5_ of product ions with positive charge on C3 shown on the right side of Scheme S1. The C‐C bonds broken during fragmentation steps are highlighted using a transversal dashed line. Exact *m/z* ratios, rounded off to the fourth decimal place, are reported.
**Figure S16.** Mechanisms hypothesized to explain the generation of specific product ions arising from the [M + H‐H_2_O]^+^ precursor ions of isofucosterol and reported in Scheme 3. The C‐C bonds broken during fragmentation steps are highlighted using a transversal dashed line. Exact *m/z* ratios, rounded off to the fourth decimal place, are reported.
**Figure S17.** Mechanisms hypothesized to explain the generation of specific product ions arising from the [M + H‐H_2_O]^+^ precursor ions of Δ^7^‐avenasterol and reported in Scheme 3. The C‐C bonds broken during fragmentation steps are highlighted using a transversal dashed line. Exact *m/z* ratios, rounded off to the fourth decimal place, are reported.
**Figure S18.** Alterative fragmentation pathway proposed to explain the enhanced generation of a product ion with a *m/z* ratio consistent with the exact value 83.0855 upon fragmentation of the [M + H^+^‐H_2_O]^+^ ion of Δ^7^‐avenasterol. Exact *m/z* ratios, rounded off to the fourth decimal place, are reported.
**Scheme S1.** Fragmentation pathways hypothesized for stigmasterol and stigmasterol penta‐deuterated on the ethyl group linked to C24 considering their [M + H‐H_2_O]^+^ ions generated in the APCI source as precursor ions, with the positive charge located on different possible sites. Plain arrows indicate fragmentations observed in MS/MS spectra (under both HCD and CID conditions); the dashed arrow indicate fragmentations observed in the 395 > 297 > CID‐MS^3^ spectrum. Boxes include multiple product ions observed in a single MS^n^ (*n* = 2–3) spectrum. Exact *m/z* ratios (rounded off to the fourth decimal place) are reported for all product ions. Values between parentheses are referred to deuterated stigmasterol. Note that structures hypothesized for product ions belonging to clusters from D to I were not reported since they are identical to those proposed in Schemes 1 and 2 for cholesterol. See text for details.
**Table S1.** Experimental *m/z* values and relative intensities referred to peak signals detected in the HCD‐FTMS/MS spectra of cholesterol and its analogue deuterated on methyl groups linked to C25 (cholesterol‐d_6_). Molecular formulas consistent with *m/z* values (accuracy < 5 ppm) are also reported. Product ions sharing the same number of C atoms are grouped within a single cluster identified by a capital letter, as in Figure 3. The *m/z* values of cholesterol product ions for which deuterated counterpart(s) were not detected at all in the cholesterol‐d_6_ spectrum are underlined; those referred to product ions for which only hexa‐deuterated analogues were detected in the cholesterol‐d_6_ spectrum are reported with bold character.
**Table S2.** Experimental m/z values and relative intensities referred to peak signals detected in the HCD‐FTMS/MS spectra of stigmasterol and its analogue penta‐deuterated on the ethyl group linked to C24 (stigmasterol‐d_5_). Molecular formulas consistent with *m/z* values (accuracy < 5 ppm) are also reported. Product ions sharing the same number of C atoms are grouped within a single cluster, identified by a capital letter, as in Figure 5. The *m/z* values of stigmasterol product ions for which deuterated counterpart(s) were not detected at all in the stigmasterol‐d_5_ spectrum are underlined; those referred to product ions for which only penta‐deuterated analogues were detected in the cholesterol‐d6 spectrum are reported with bold character.
**Table S3.** Summary of typical relative intensities obtained for specific ions detected in APCI(+)‐HCD‐FTMS/MS spectra acquired for the [M + H‐H_2_O]^+^ ions of sterols investigated in the present study. Experimental *m/z* values and molecular formulas inferred from them considering an accuracy not higher than 5 ppm are also reported. Ions are grouped according to the number of C atoms in their structures, in clusters labelled as in Figures 3, 4 and 5, according to the case. The prevailing relative intensity in each cluster is reported in bold character.
