# Supplementary material for: APCI‐Multistage Mass Spectrometry Following Liquid Chromatography for Selected 4‐Desmethyl‐Sterols and Their Deuterium‐Labelled Analogues Unveils Characteristic Fragmentation Routes for Cholesterol and Phytosterols Identification
Source: Rapid Commun Mass Spectrom. 2025 Apr 5;39(13):e10039. doi: 10.1002/rcm.10039 (PMC11972017; doi:10.1002/rcm.10039)
Supplement: Supplementary file 1 — Figure S1. Mechanisms hypothesized to explain the generation of low m/z (< 100) product ions shown in Scheme 1 from the [M + H‐H2O]+ ions of cholesterol (CHO) and of cholesterol (CHO‐d6) completely deuterated on the methyl groups linked to C25. For the sake of clarity, the transfer of H, with concurrent breakage of a C‐C bond and the formation of a new C=C bond is depicted explicitly only in the first process drawn in the scheme. Nonetheless, the C‐C bond broken as a consequence of this process is highlighted in all fragmentation reactions using a transversal dashed line. Exact m/z ratios, rounded off to the fourth decimal place, are reported. Figure S2. Genealogical trees constructed for sterol [M + H‐H2O]+ ions to describe the information inferred from HCD‐FTMS/MS and CID‐MS3/MS4 acquisitions performed, respectively, using a quadrupole‐Orbitrap and a linear ion trap mass spectrometer. The m/z ratio of the precursor ion involved in the HCD‐FTMS/MS acquisition is reported on top of each tree and is then connected to boxes representing the m/z ratios and percentual relative intensities observed in the HCD‐FTMS/MS spectrum, for the resulting product ions that were subsequently subjected to CID‐MS3 acquisitions (note that the same set of product ions was observed in HCD‐FTMS/MS and in CID‐MS/MS spectra of sterol [M + H‐H2O]+ ions). Each of those boxes is then connected to a series of boxes describing m/z ratios and relative intensities observed for ions detected in CID‐MS3 spectra. Among the latter, boxes with m/z ratios and relative intensities written in blue colour represent precursor ions selected for subsequent CID‐MS4 acquisitions. In this case, the set of m/z ratios of major peak signals detected in the MS4 spectrum was not explicitly indicated since it corresponded to the set inferred from the MS3 spectrum acquired on the same precursor ion and reported in one of the genealogical trees for the same sterol. Notes: (1) The m/z ratios of ions including D atoms in [file RCM-39-e10039-s001.docx]

**Supporting Information**

**APCI-multistage mass spectrometry following liquid chromatography for selected 4-desmethyl-sterols and their deuterium-labelled analogues unveils characteristic fragmentation routes for cholesterol and phytosterols identification**

V. Cinquepalmi^a^, I. Losito^a,b,*^, A. Castellaneta^a^, C.D. Calvano^a,b^, T.R.I. Cataldi^a,b^

^a^Dipartimento di Chimica and ^b^Centro Interdipartimentale SMART – Università degli Studi di Bari, via E. Orabona 4, Bari;

Number of Figures:18

Number of Tables: 3

Number of Schemes: 1


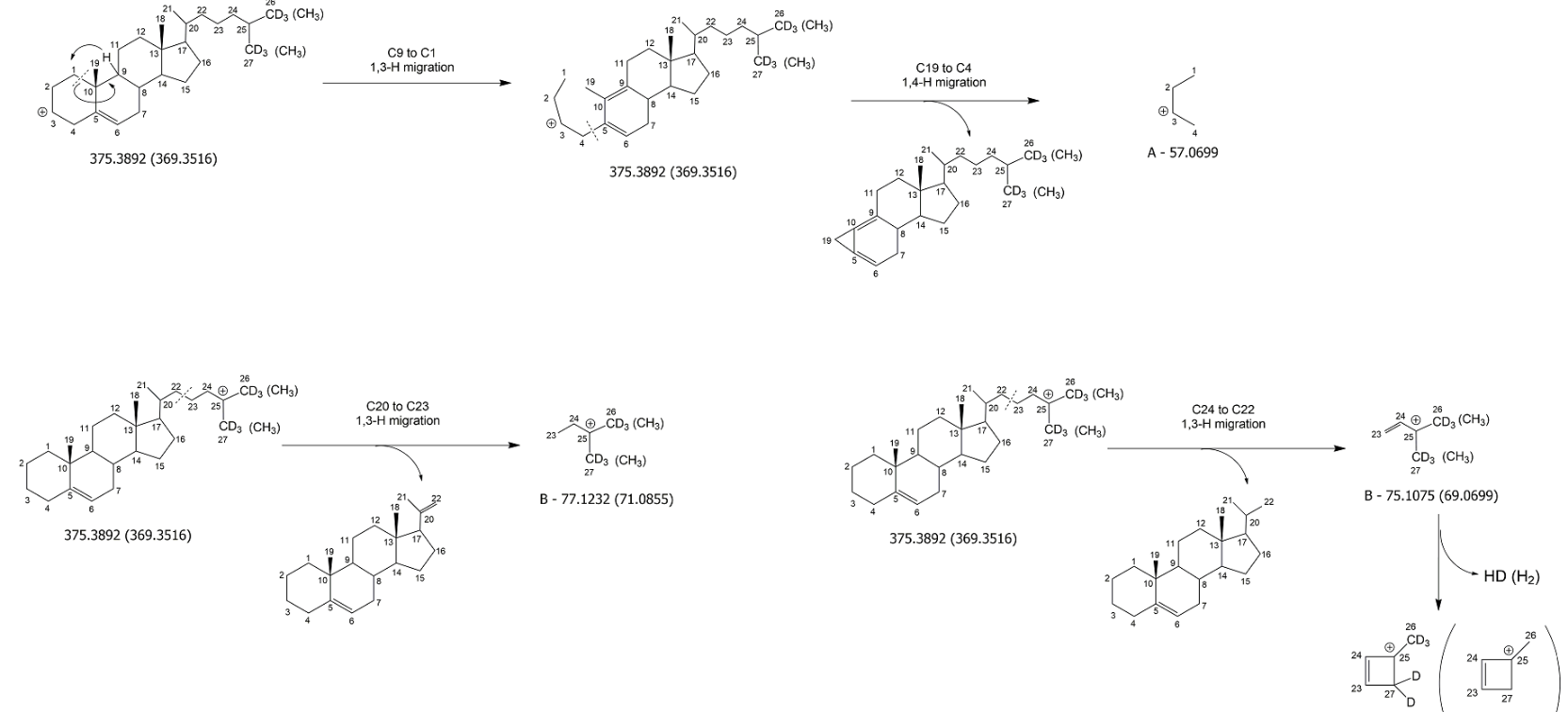

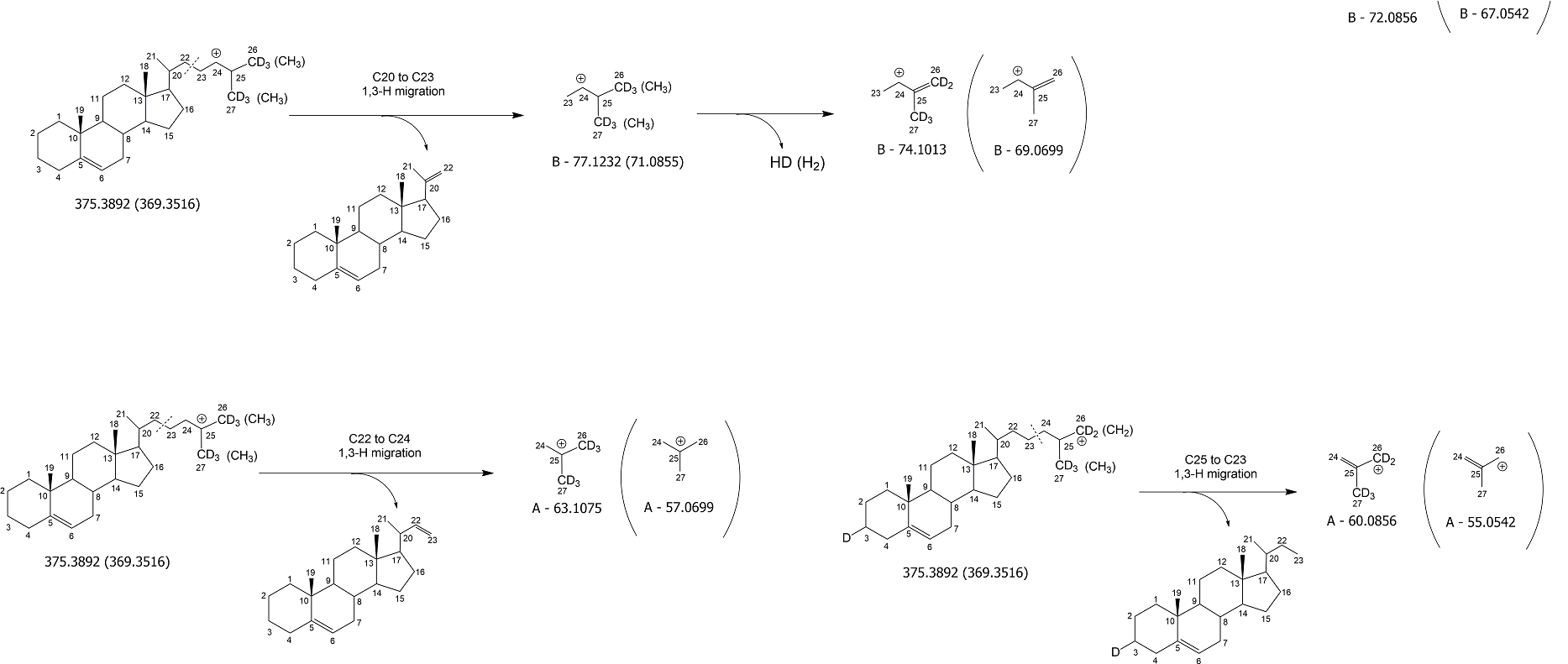


**Figure S1**. Mechanisms hypothesised to explain the generation of low *m/z* (< 100) product ions shown in Scheme 1 from the [M+H-H_2_O]^+^ ions of cholesterol (CHO) and of cholesterol (CHO-d_6_) completely deuterated on the methyl groups linked to C25. For the sake of clarity, the transfer of H, with concurrent breakage of a C-C bond and the formation of a new C=C bond is depicted explicitly only in the first process drawn in the scheme. Nonetheless, the C-C bond broken as a consequence of this process is highlighted in all fragmentation reactions using a transversal dashed line. Exact *m/z* ratios, rounded off to the fourth decimal place, are reported.

**Figure S2**. Genealogical trees constructed for sterol [M+H-H_2_O]^+^ ions to describe the information inferred from HCD-FTMS/MS and CID-MS^3/^MS^4^ acquisitions performed, respectively, using a quadrupole-Orbitrap and a linear ion trap mass spectrometer. The *m/z* ratio of the precursor ion involved in the HCD-FTMS/MS acquisition is reported on top of each tree and is then connected to boxes representing the *m/z* ratios and percentual relative intensities observed, in the HCD-FTMS/MS spectrum, for the resulting product ions that were subsequently subjected to CID-MS^3^ acquisitions (note that the same set of product ions was observed in HCD-FTMS/MS and in CID-MS/MS spectra of sterol [M+H-H_2_O]^+^ ions). Each of those boxes is then connected to a series of boxes describing *m/z* ratios and relative intensities observed for ions detected in CID-MS^3^ spectra. Among the latter, boxes with *m/z* ratios and relative intensities written in blue colour represent precursor ions selected for subsequent CID-MS^4^ acquisitions. In this case, the set of *m/z* ratios of major peak signals detected in the MS^4^ spectrum was not explicitly indicated since it corresponded to the set inferred from the MS^3^ spectrum acquired on the same precursor ion and reported in one of the genealogical trees for the same sterol. Notes: 1) the *m/z* ratios of ions including D atoms in their structures are written in red character; 2) the letter adopted to mark each product ion cluster in FTMS/MS spectra has been reported also for all ions inside the trees; 3) for the sake of consistency, all *m/z* ratios have been rounded to the first decimal place, in accordance with the precision level available with the linear ion trap mass spectrometer. The figure follows in the next seven pages.


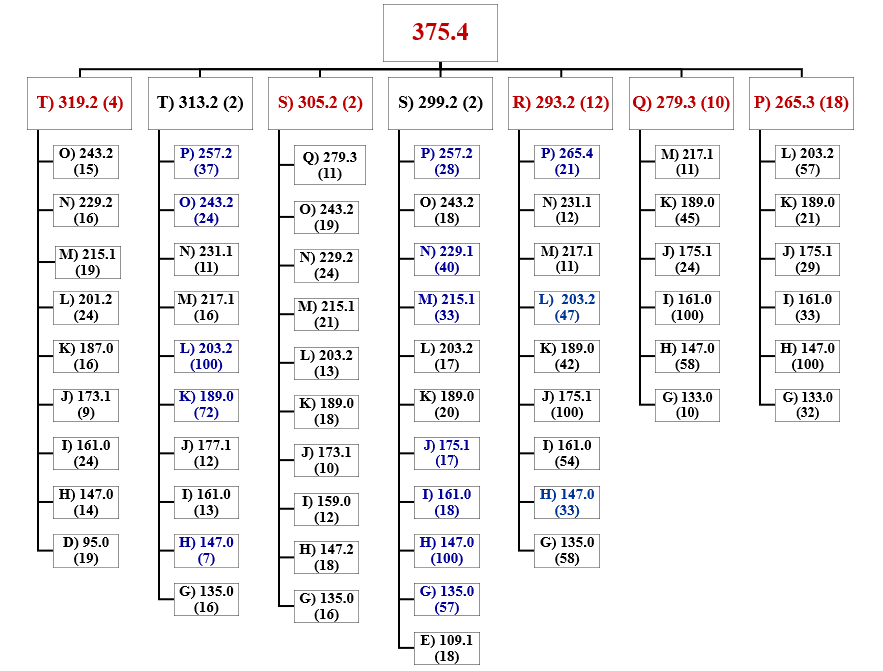
Cholesterol-d_6_ (first part)

Cholesterol-d_6_ (second part)

_
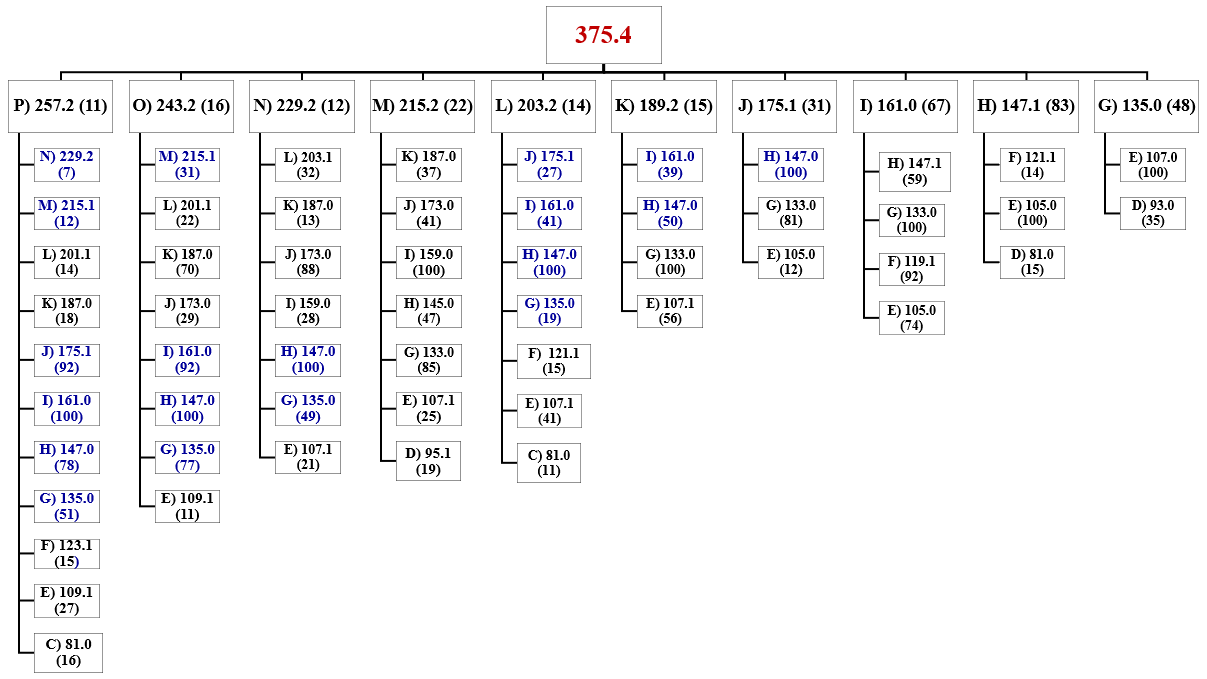
_

Cholesterol

*
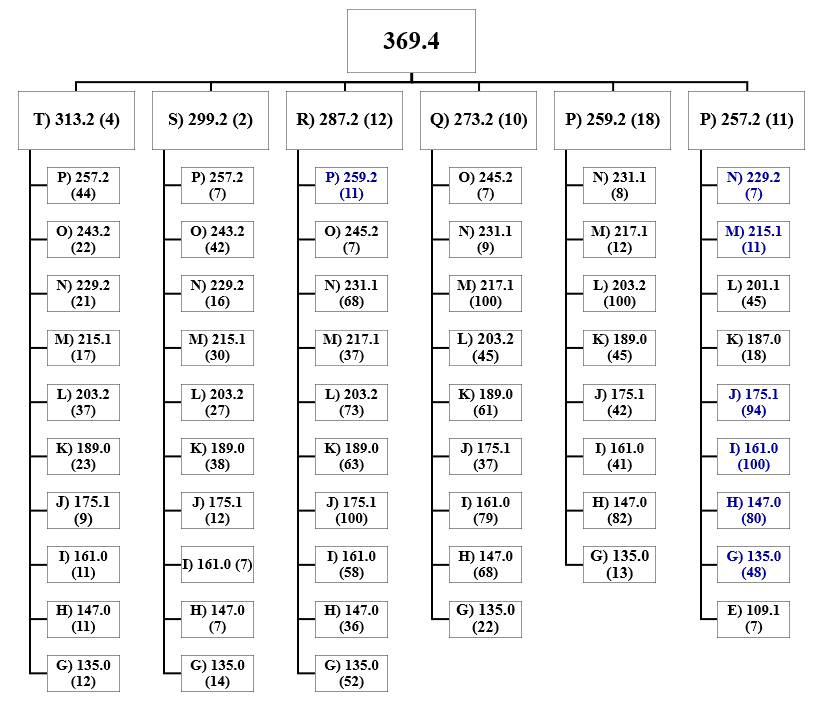
*

**Note**: in order to avoid redundancy, only MS^3^ and MS^4^ transitions characteristic of cholesterol [M+H-H_2_O]^+^ ions are represented in this scheme. Indeed, MS^3^ spectra obtained for ions of clusters O to G shown in the second genealogical tree of cholesterol-d_6_ [M+H-H_2_O]^+^ ions (see page 4) were practically identical to those obtained for the corresponding precursor ions generated from the cholesterol [M+H-H_2_O]^+^ ions, since the precursor ions had the same structure in both cases.


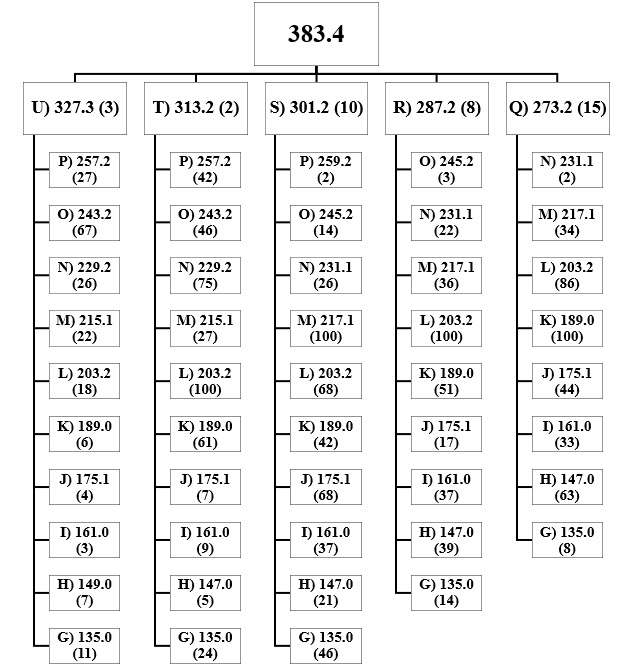
Campesterol
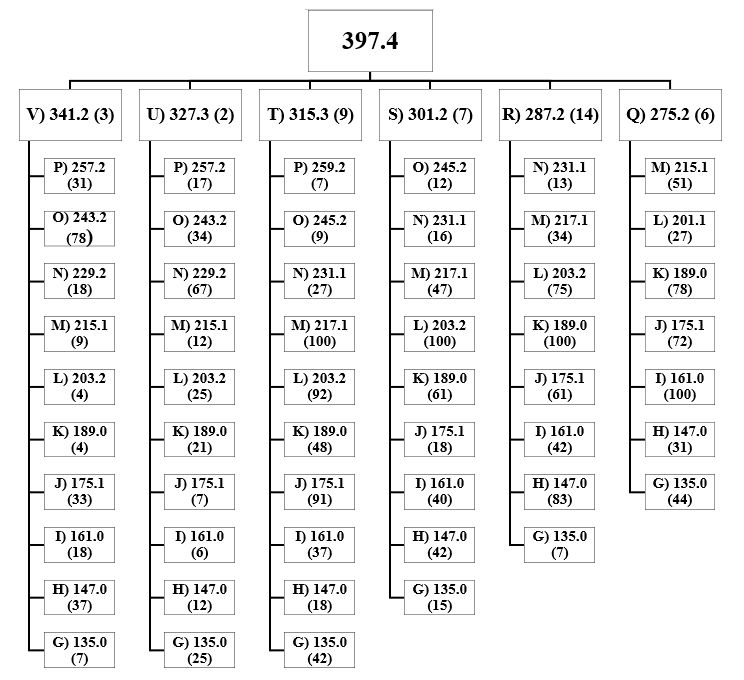
β-sitosterol

Stigmasterol-d_5_ (first part)


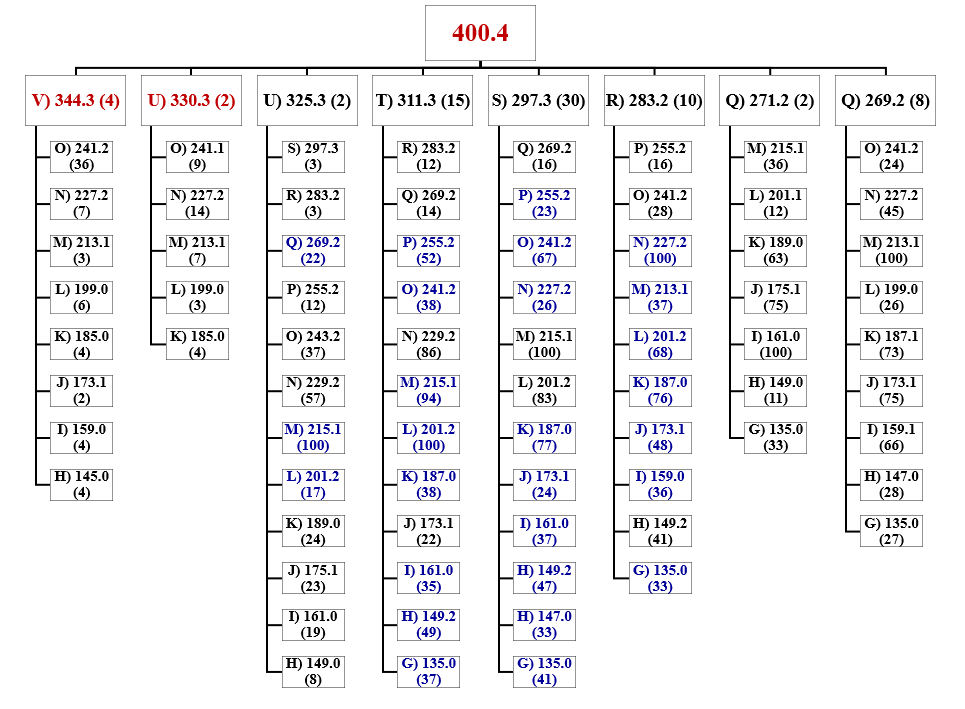


Stigmasterol-d_5_ (second and third part)


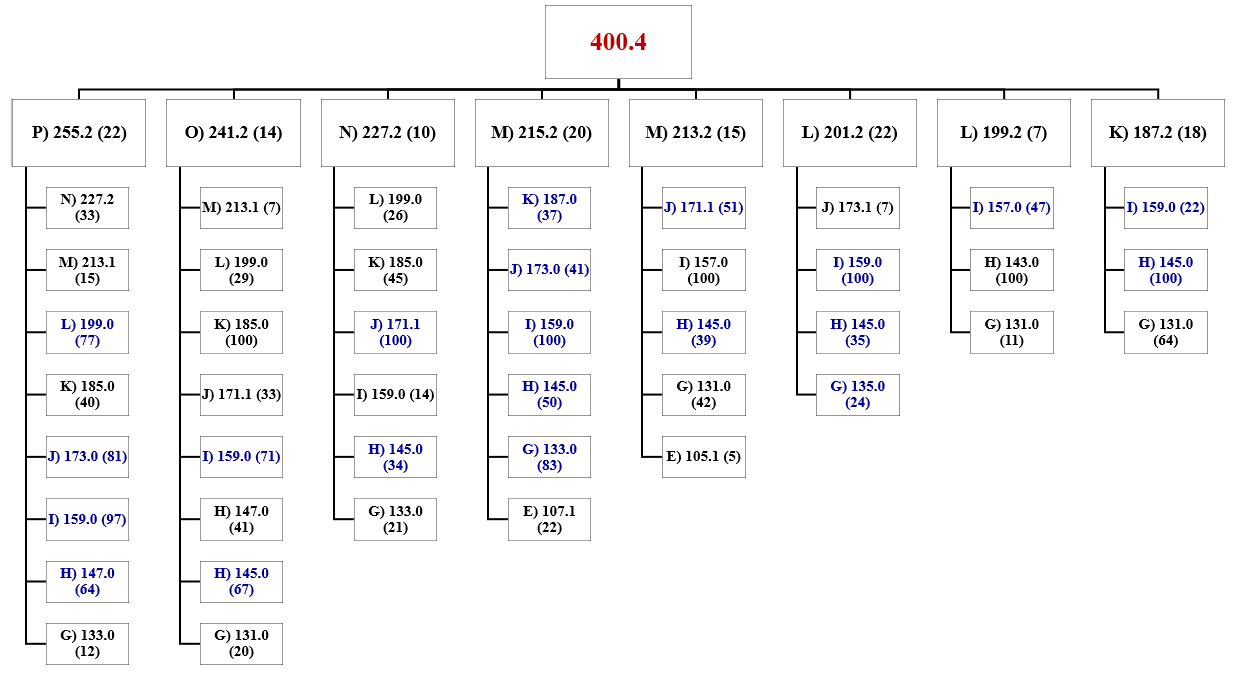


*
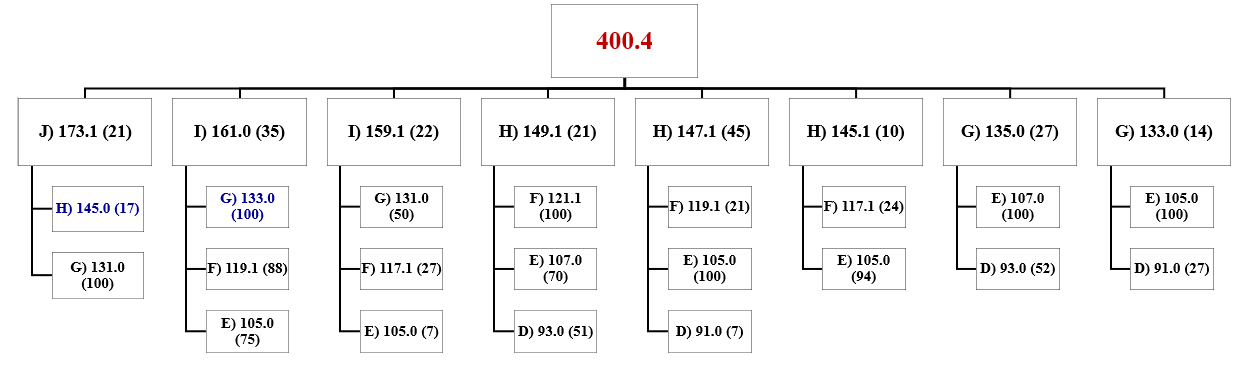
*

Stigmasterol

*
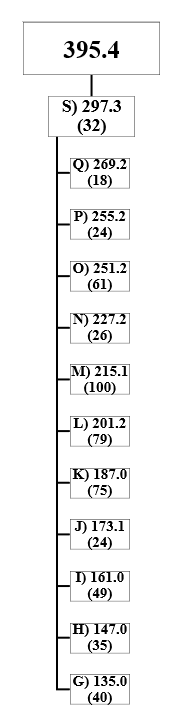
*


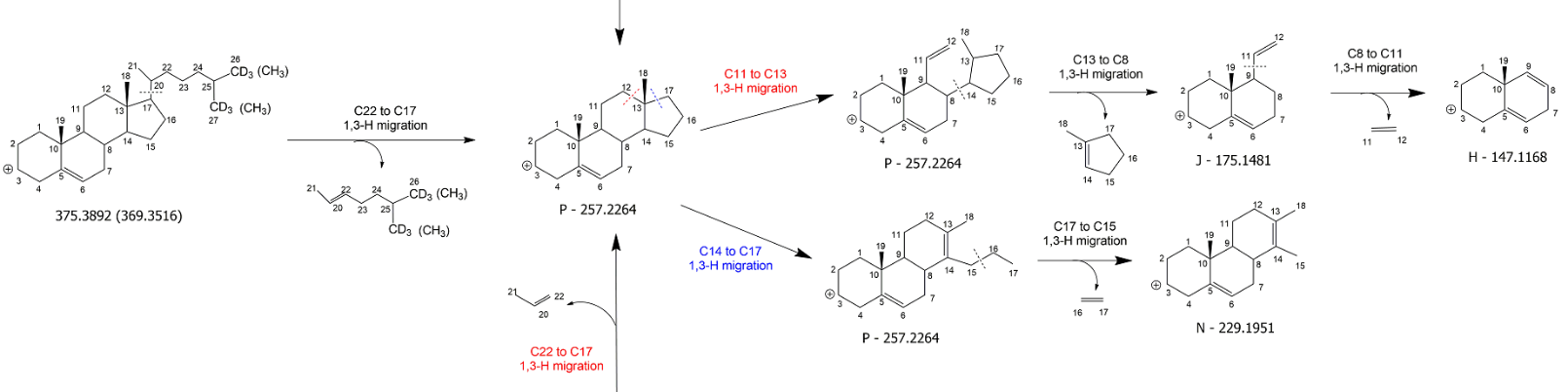

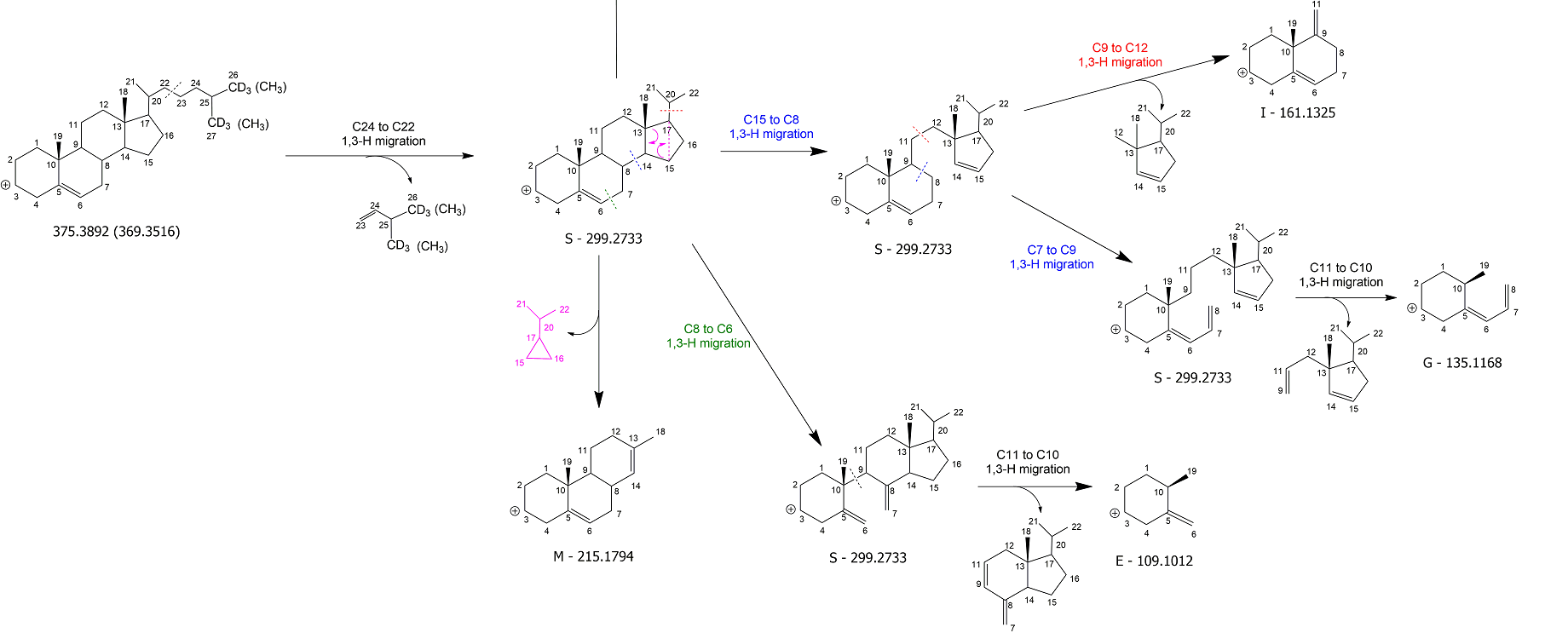

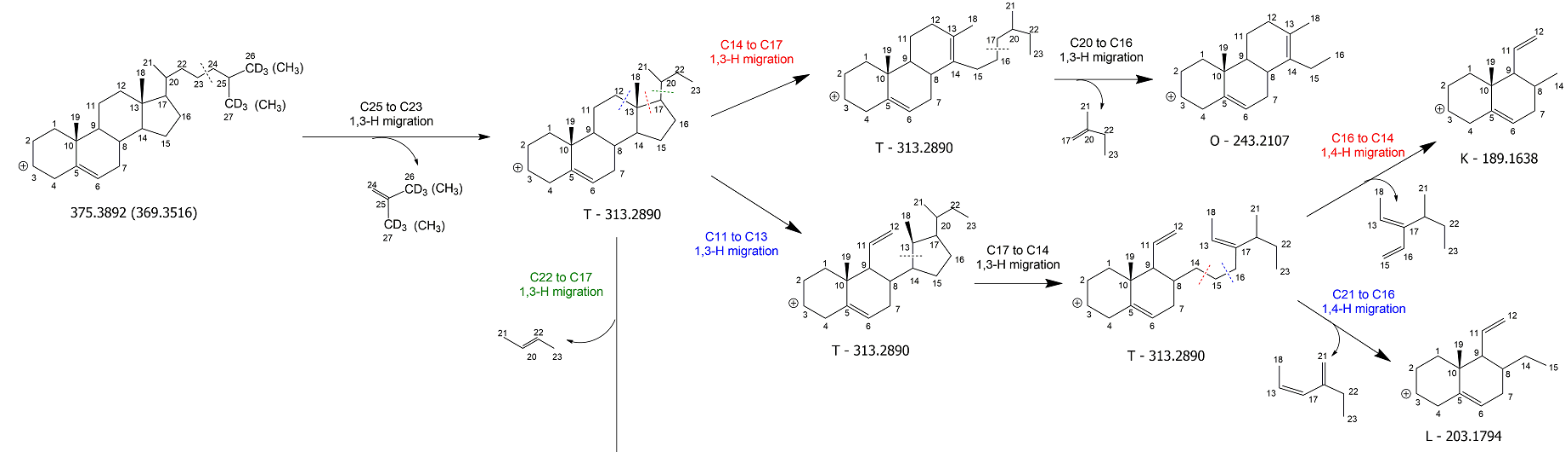


**Figure S3**. Mechanisms hypothesised to explain the generation of product ions with *m/z* > 100 shown in Scheme 1 from the [M+H-H_2_O]^+^ precursors ions of cholesterol (CHO) and of cholesterol-d_6_ (CHO-d_6_). The C-C bonds broken during fragmentation steps are highlighted using a transversal dashed line, eventually coloured to relate the bond breakage to a specific process. Exact *m/z* ratios, rounded off to the fourth decimal place, are reported.





**Figure S4**. Mechanism potentially responsible for the formation of the product ion detected at *m/z* 81.0705 directly from the [M+H-H_2_O]^+^ ion of cholesterol and cholesterol-d6. It involves a retro-cycloaddition at the C-ring and the removal of the side chain through a 1,3-H transfer from C22 to C17, with breakage of the C17-C20 bond and formation of a C=C bond between C20 and C22. Exact *m/z* ratios, rounded off to the fourth decimal place, are reported.


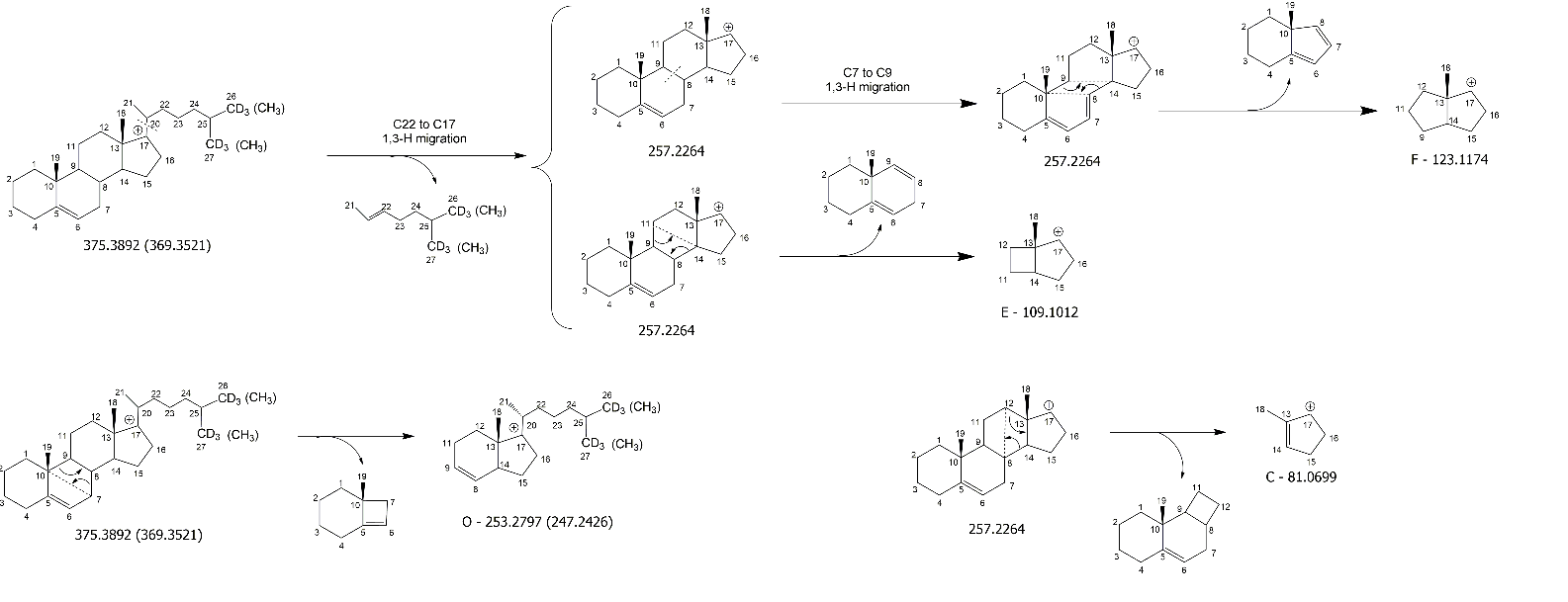

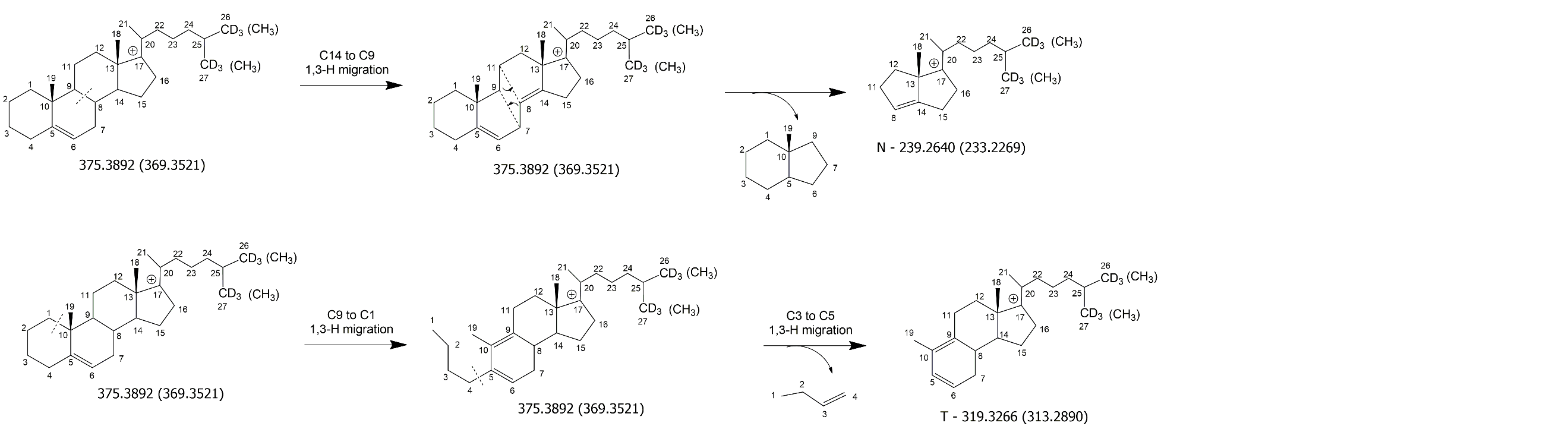

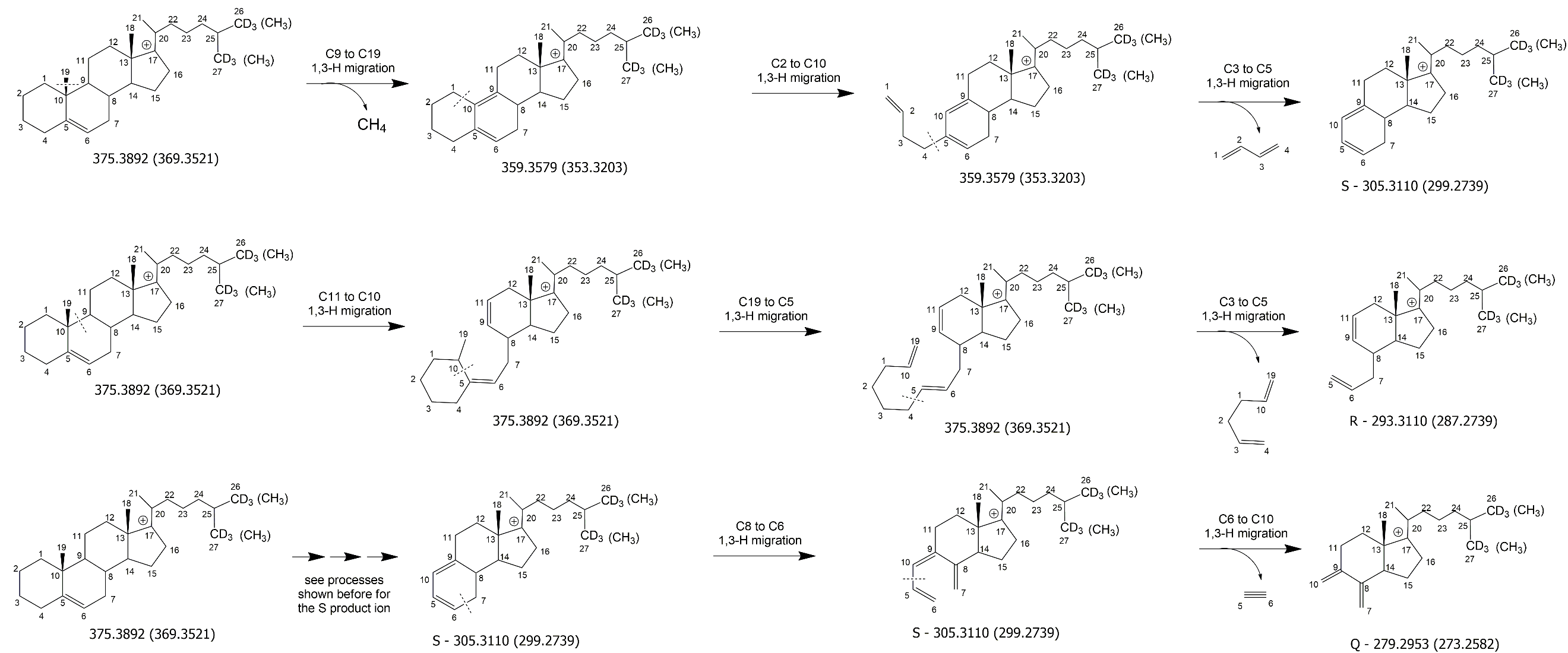


**Figure S5**. Mechanisms hypothesised to explain the generation of product ions shown in the upper half of Scheme 2 from the [M+H-H_2_O]^+^ precursors ions of cholesterol (CHO) and of cholesterol-d_6_ (CHO-d_6_). The C-C bonds broken during fragmentation steps are highlighted using a transversal dashed line. Exact *m/z* ratios, rounded off to the fourth decimal place, are reported.


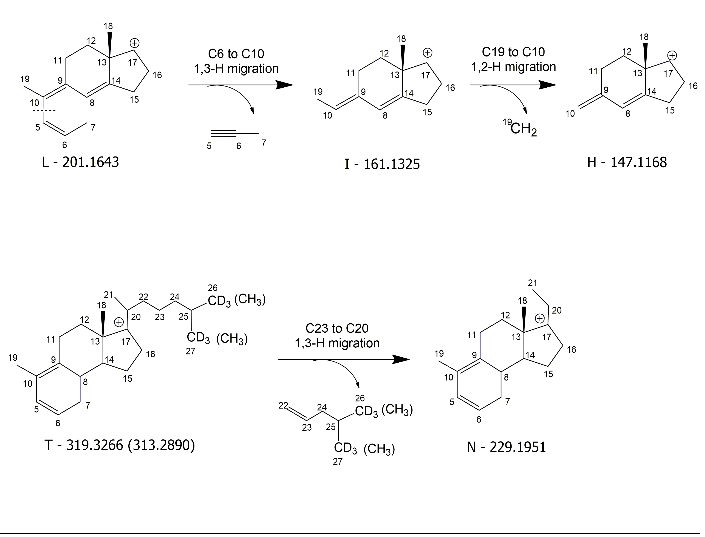

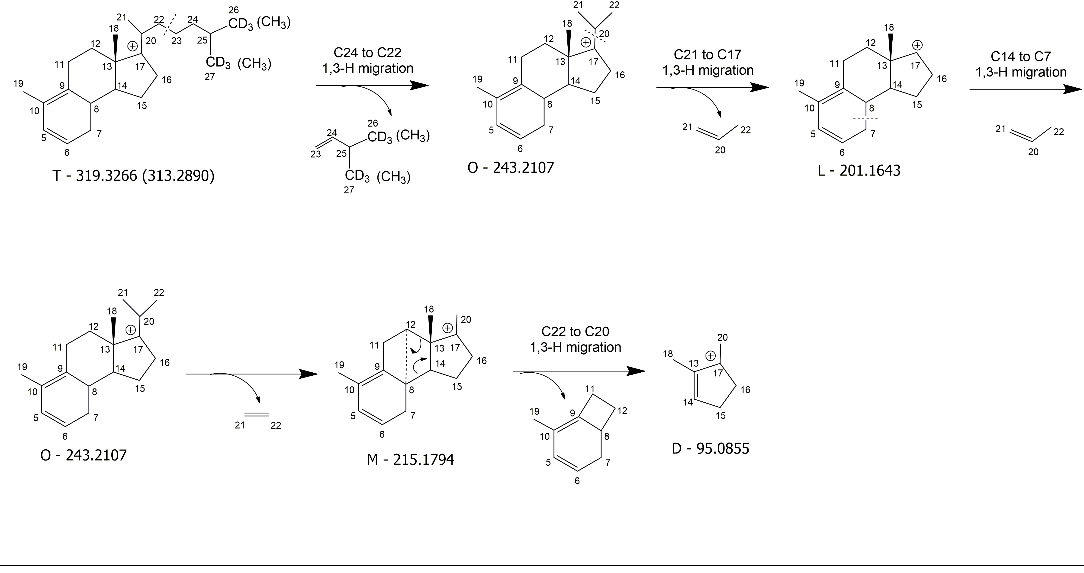

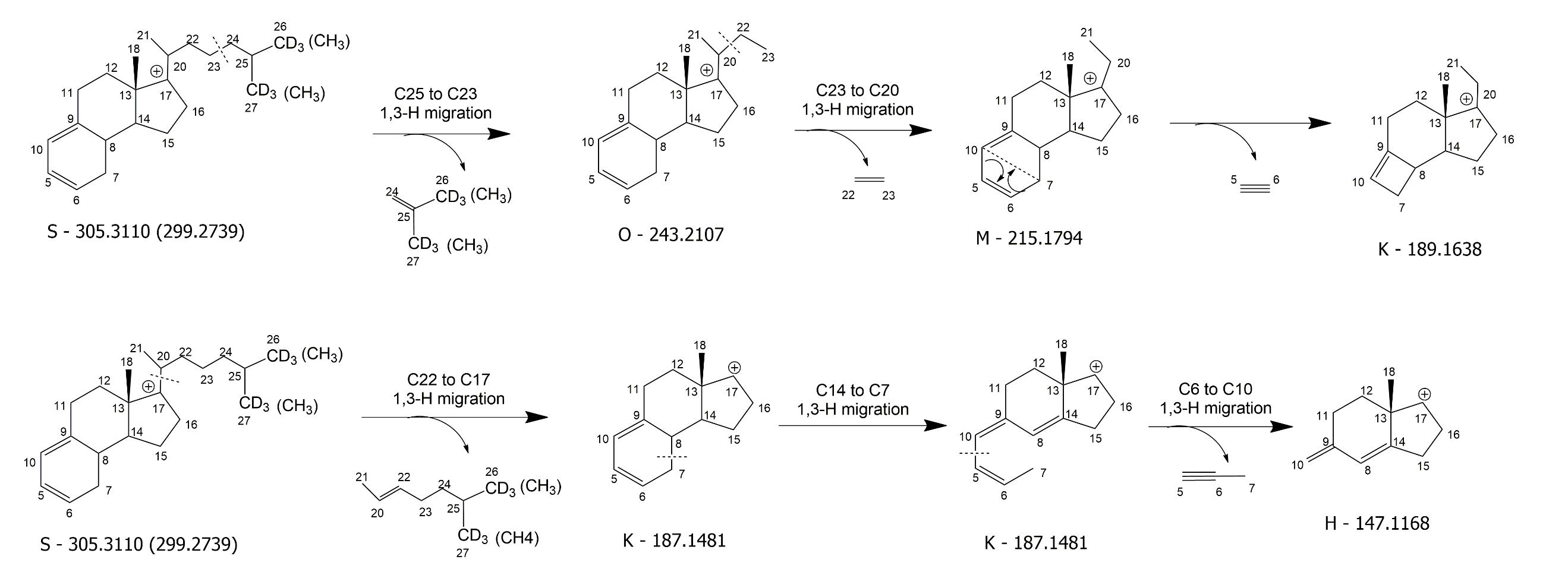

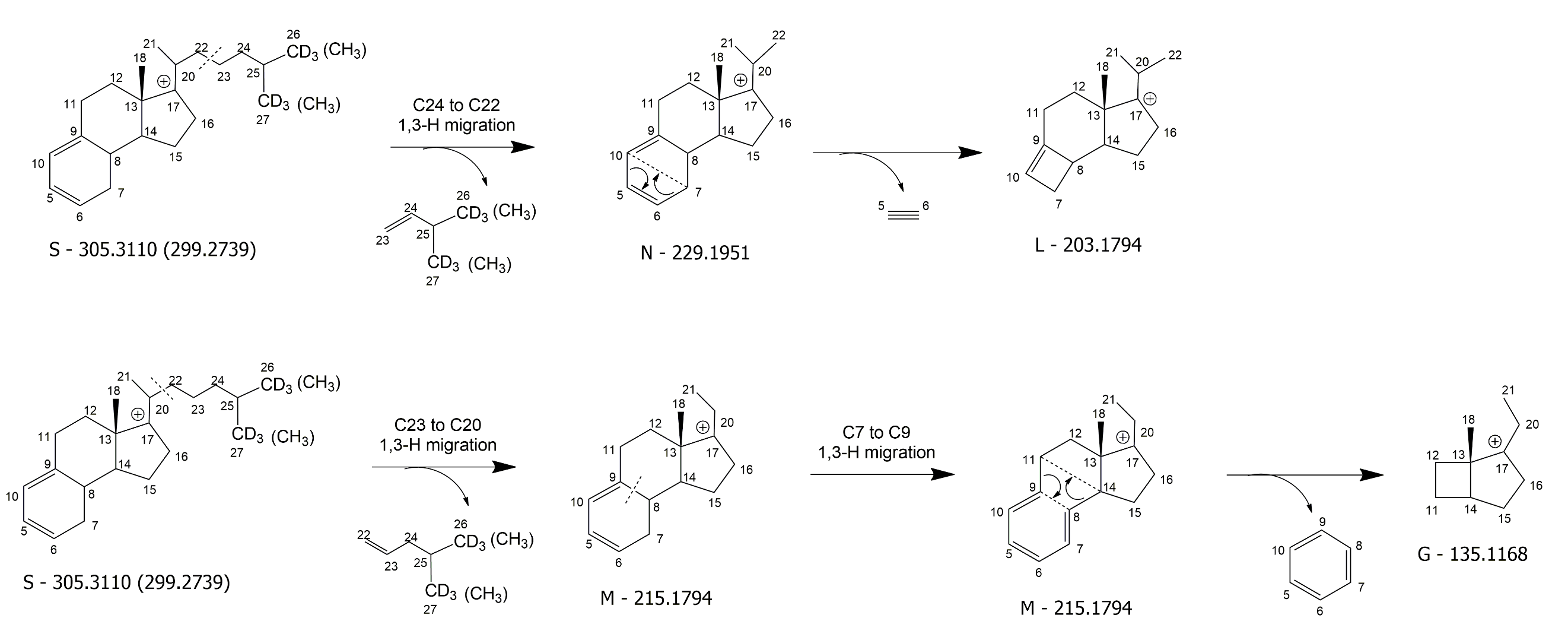


**Figure S6**. Mechanisms hypothesised to explain the generation of product ions related to ions with *m/z* 319.3266/313.2890 (upper part) and 305.3110/299.2739 (lower part) shown in Scheme 2, representing product ions of the [M+H-H_2_O]^+^ ions of cholesterol (CHO) and of cholesterol-d_6_ (CHO-d_6_). The C-C bonds broken during fragmentation steps are highlighted using a transversal dashed line. Exact *m/z* ratios, rounded off to the fourth decimal place, are reported.


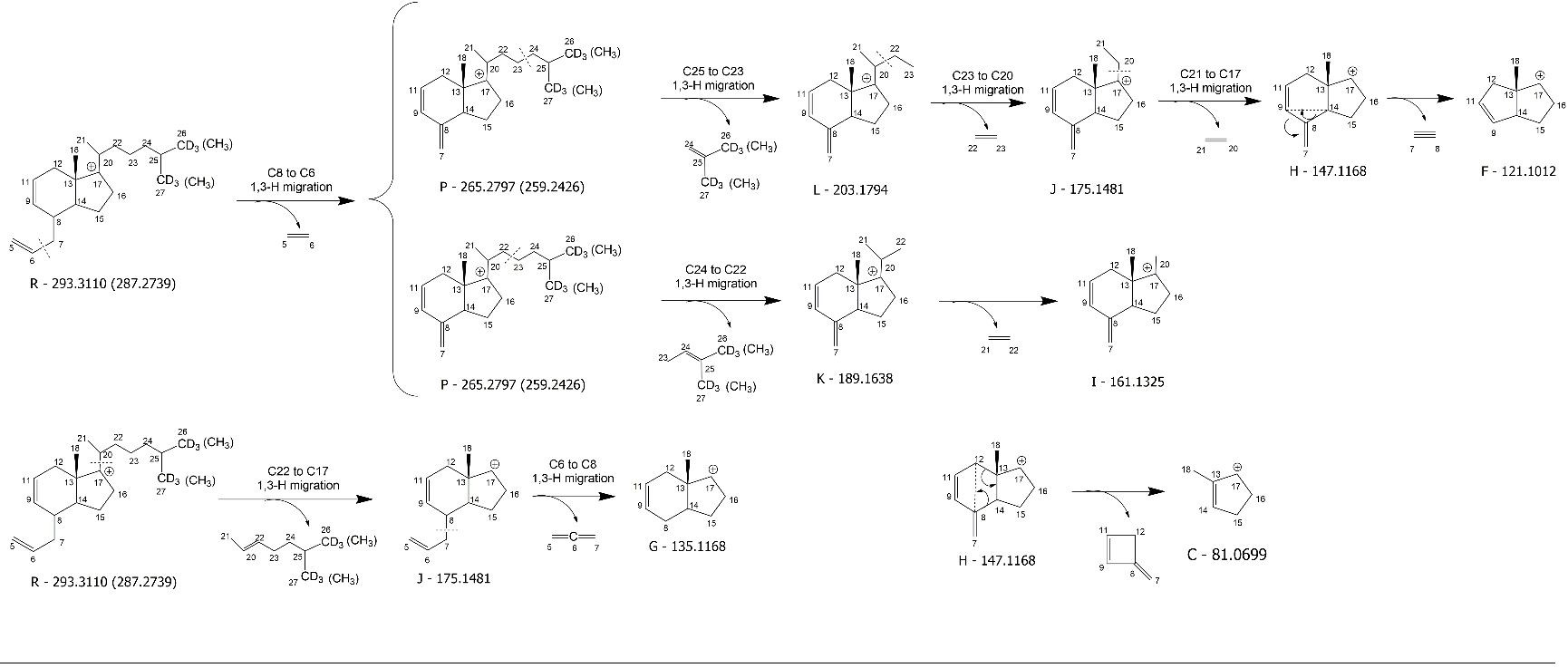

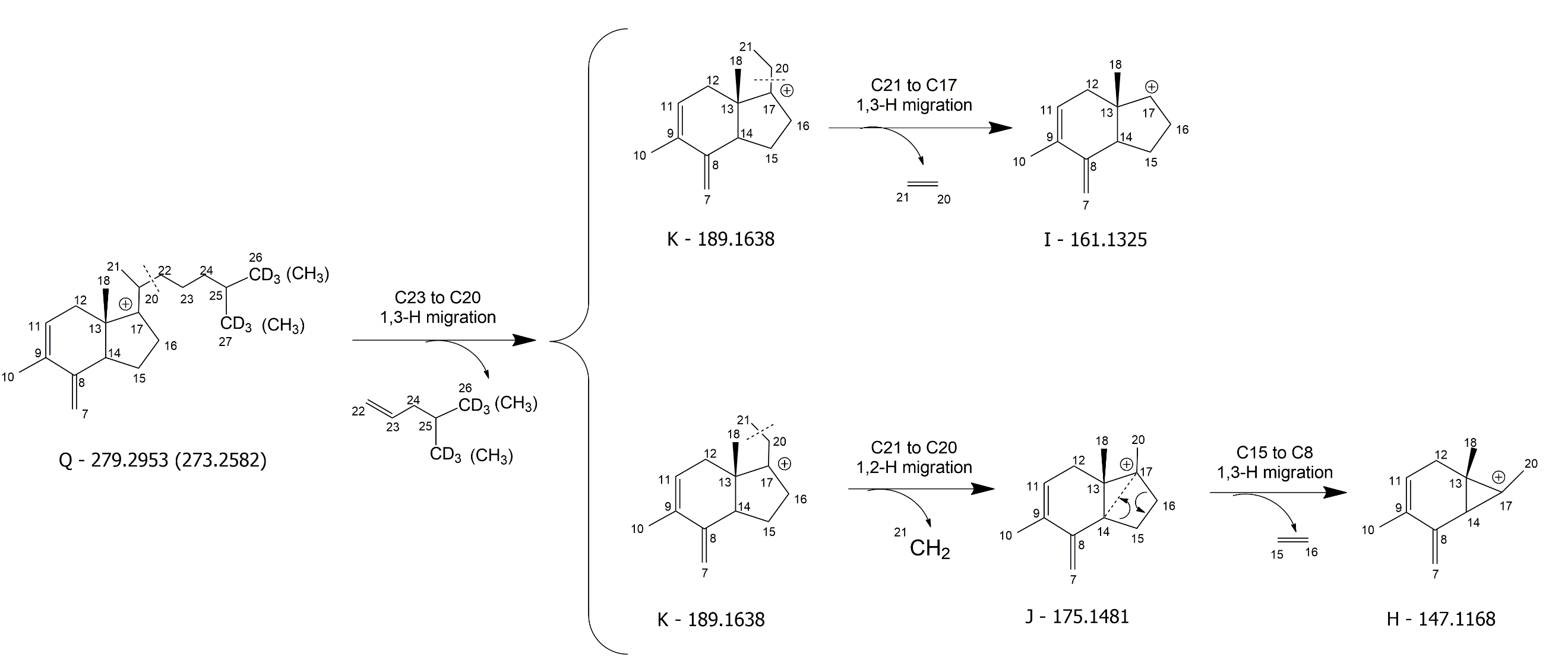


**Figure S7**. Mechanisms hypothesised to explain the generation of product ions related to ions with *m/z* 293.3110/287.2739 (upper part) and 279.2953/273.2582 (lower part) shown in Scheme 2, representing product ions of the [M+H-H_2_O]^+^ ions of cholesterol (CHO) and of cholesterol-d_6_ (CHO-d_6_). The C-C bonds broken during fragmentation steps are highlighted using a transversal dashed line. Exact *m/z* ratios, rounded off to the fourth decimal place, are reported.





**Figure S8**. Mechanism hypothesised for the generation of the product ion at *m/z* 215.1790 directly from the [M+H-H_2_O]^+^ ion of cholesterol or cholesterol-d6. The displayed process is a cycloelimination on ring D, leading also to the complete detachment of the side chain. Exact *m/z* ratios, rounded off to the fourth decimal place, are reported.


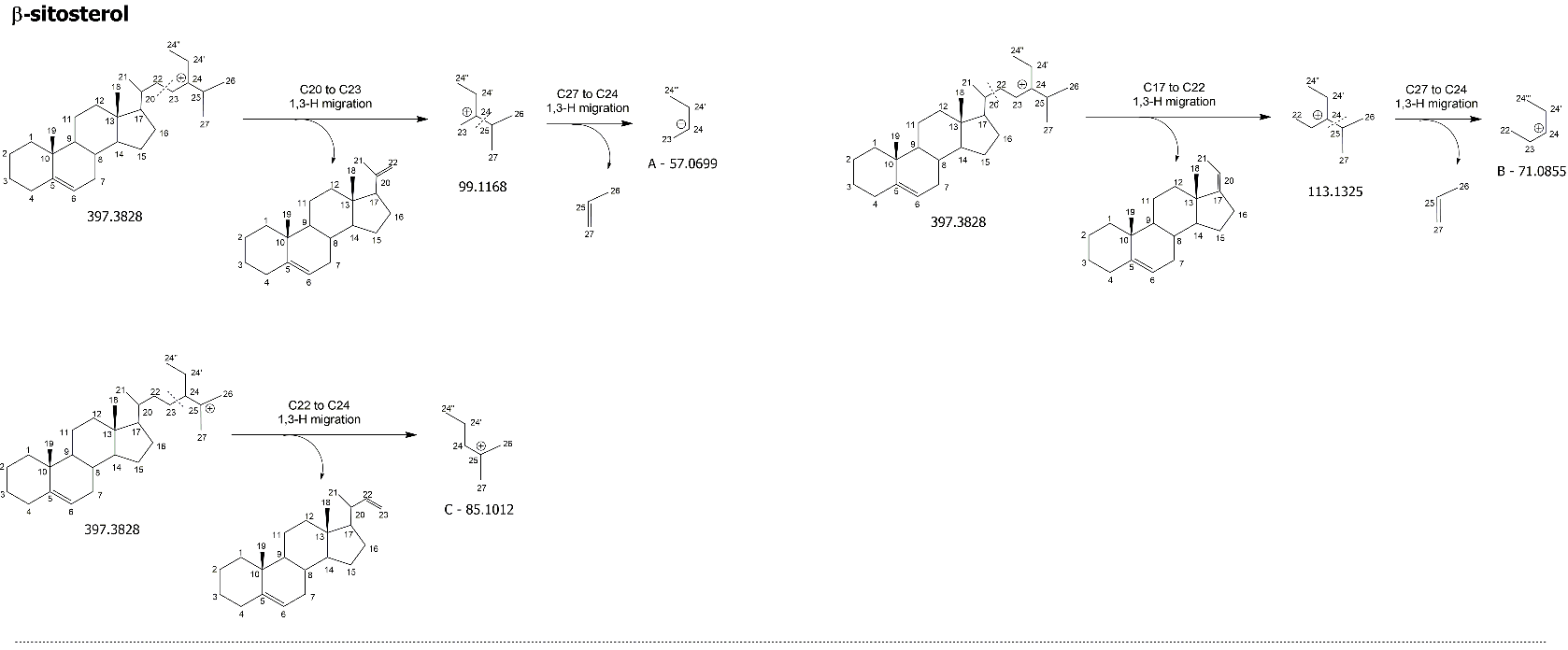

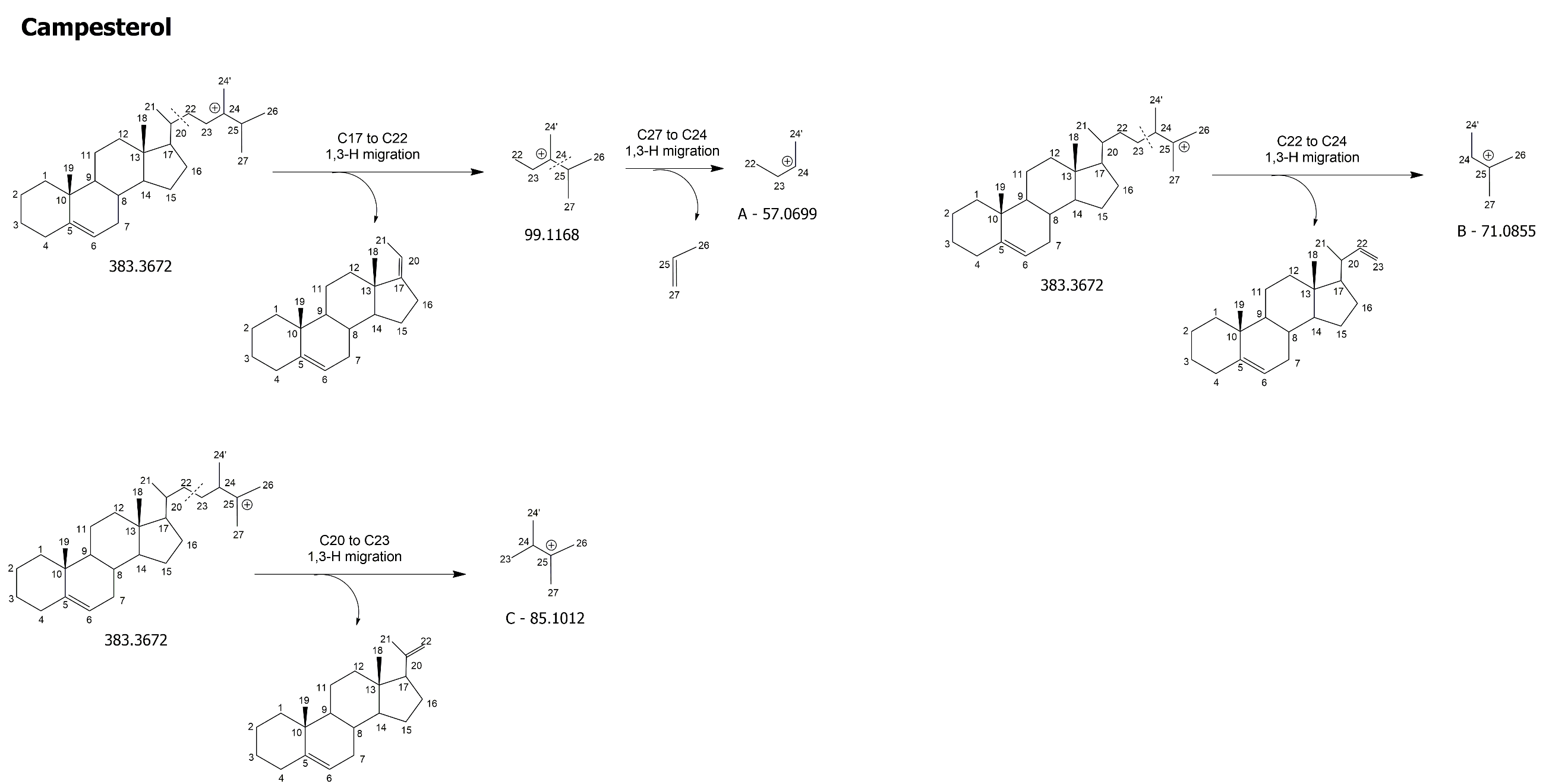


**Figure S9**. Mechanisms hypothesised to explain the generation of product ions with *m/z* < 100 from [M+H-H_2_O]^+^ ions of β-sitosterol and campesterol. The C-C bonds broken during fragmentation steps are highlighted using a transversal dashed line. Exact *m/z* ratios, rounded off to the fourth decimal place, are reported.





**Figure S10**. Alternative mechanism proposed for the generation of the product ion detected at *m/z* 257.2257 in the APCI(+)-HCD-FTMS/MS spectra of the [M+H^+^-H_2_O]^+^ ions of campesterol (R = CH_3_) and β-sitosterol (R = CH_2_CH_3_). Exact *m/z* ratios, rounded off to the fourth decimal place, are reported.


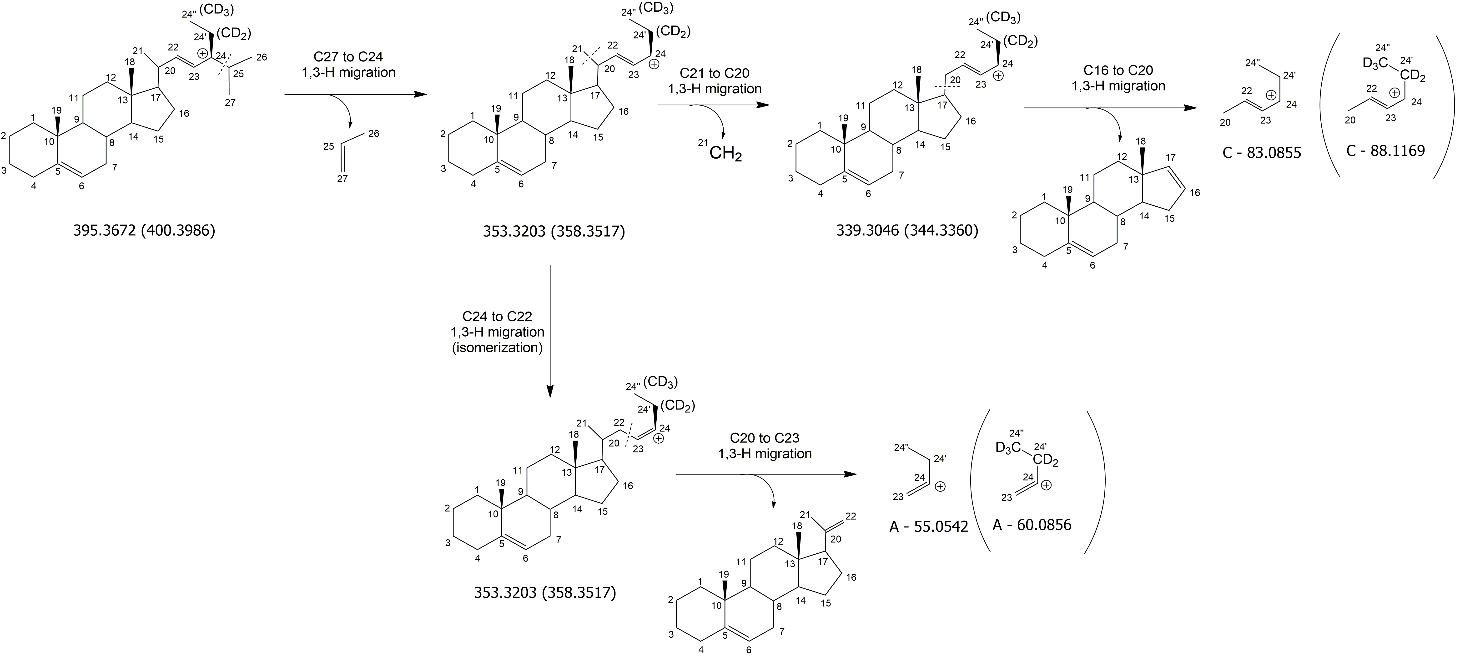

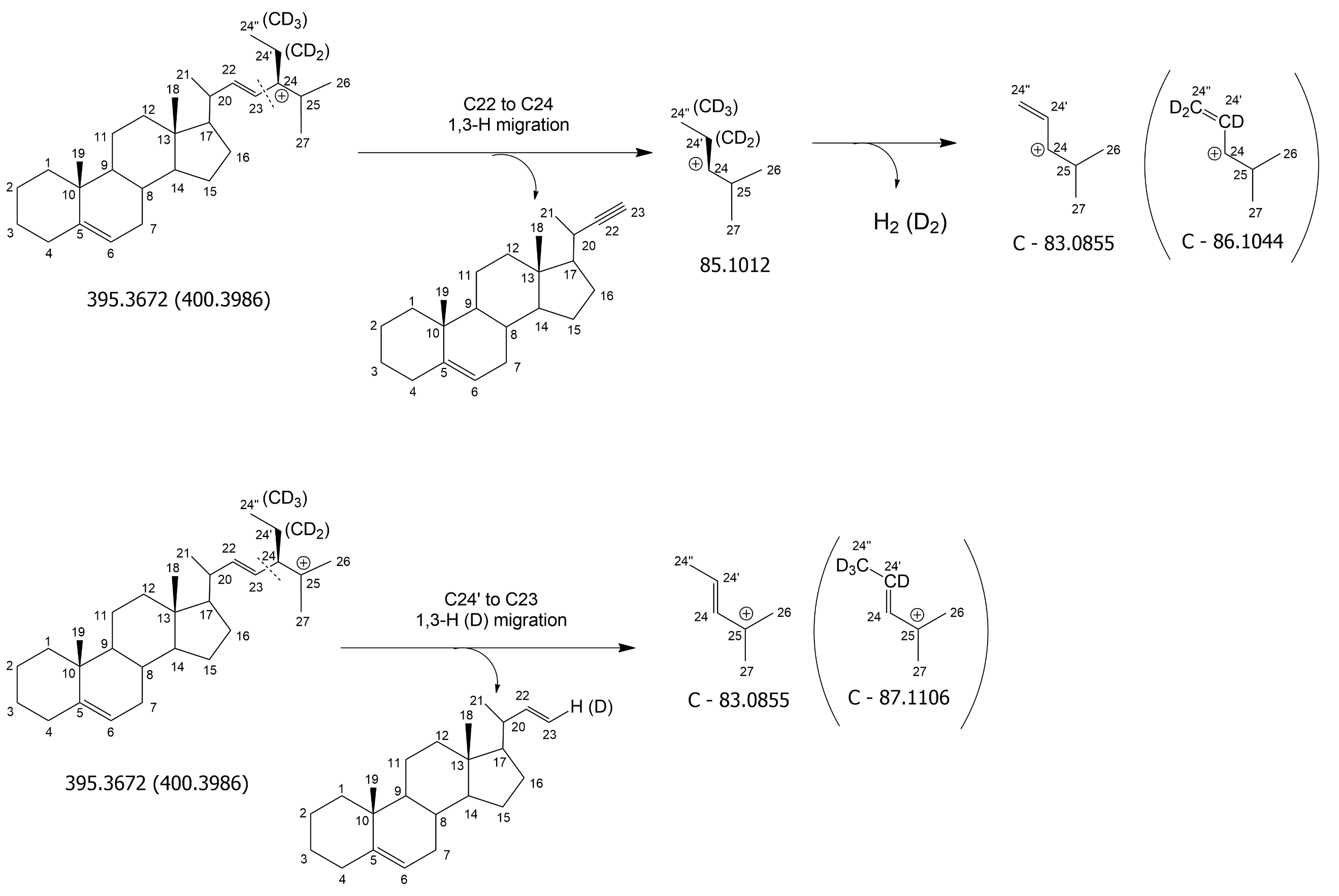

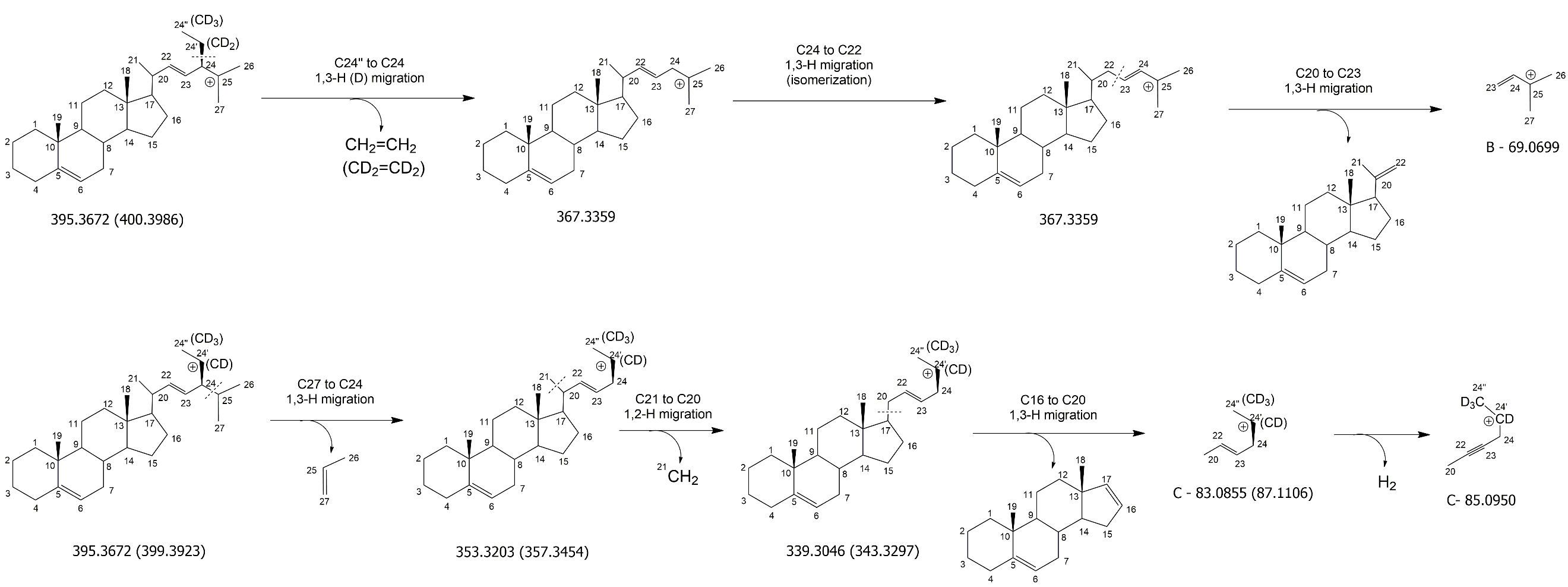


**Figure S11**. Mechanisms hypothesised to explain the generation of low *m/z* (< 100) product ions shown in Scheme S1 from the [M+H-H_2_O]^+^ precursor ions of stigmasterol and of stigmasterol completely deuterated on the ethyl group linked to C24. The C-C bonds broken during fragmentation steps are highlighted using a transversal dashed line. Exact *m/z* ratios, rounded off to the fourth decimal place, are reported.


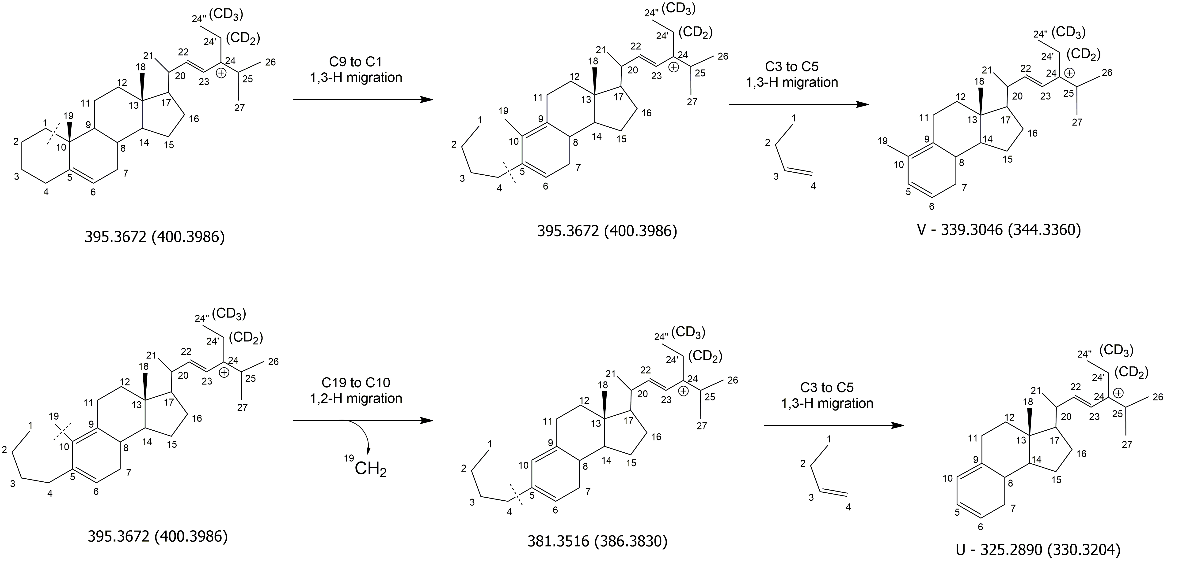

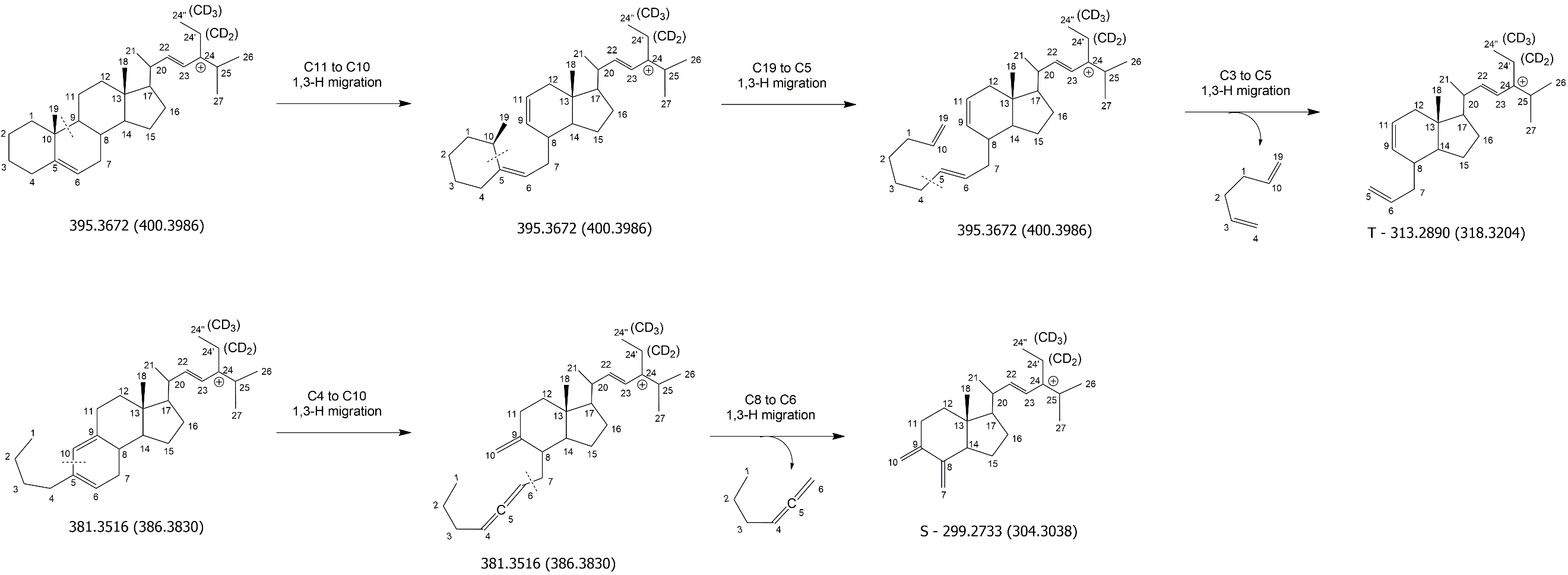

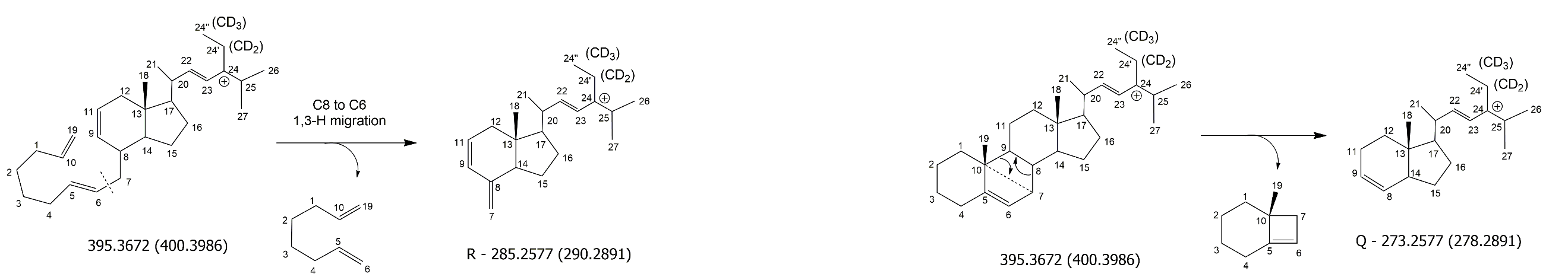


**Figure S12**. Mechanisms hypothesised to explain the generation from the [M+H-H_2_O]^+^ precursor ions of stigmasterol and stigmasterol-d_5_ of product ions with positive charge on C24 shown in Scheme S1 for clusters Q to V. The C-C bonds broken during fragmentation steps are highlighted using a transversal dashed line. Exact *m/z* ratios, rounded off to the fourth decimal place, are reported.

*
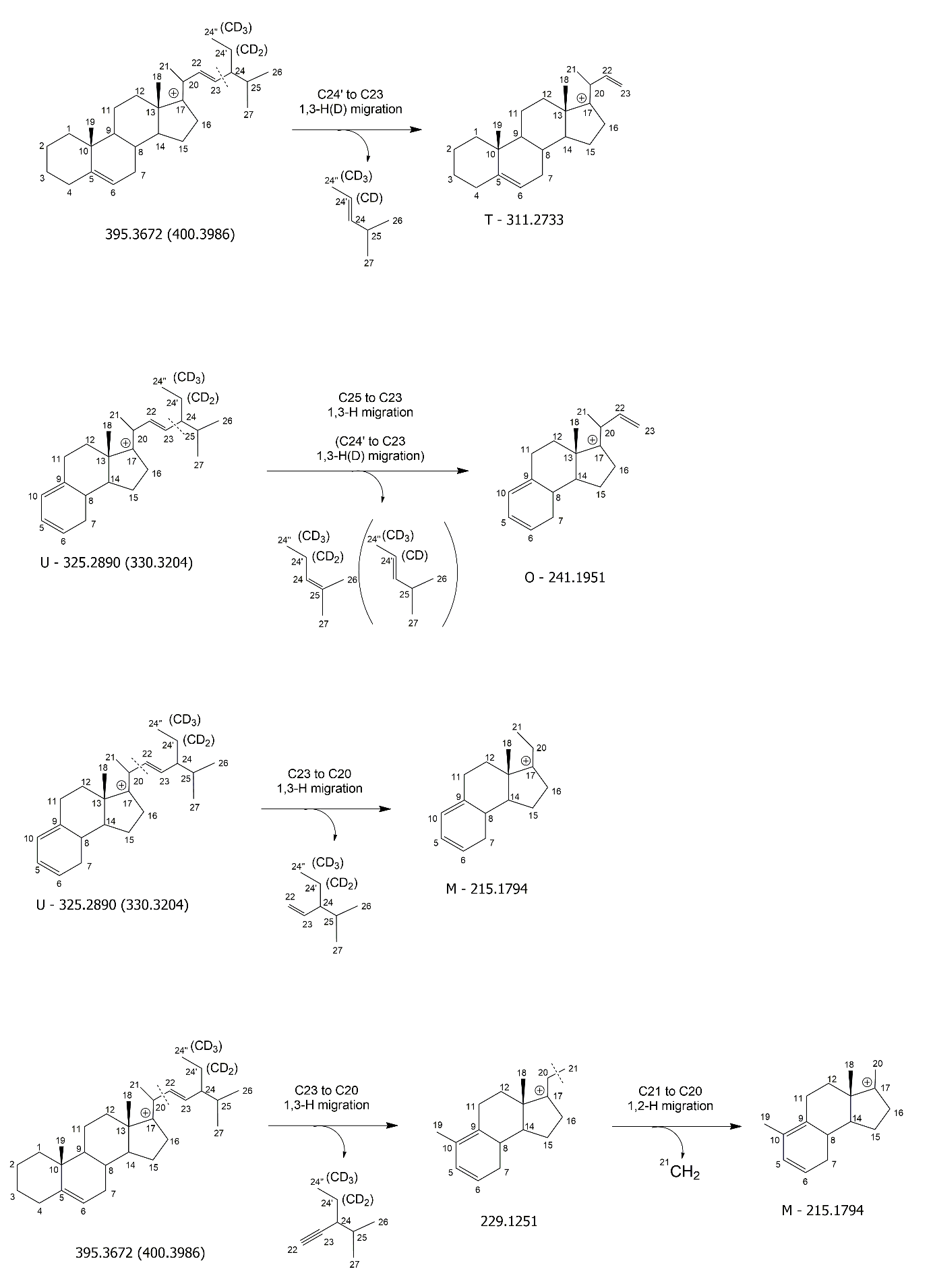
*

**Figure S13**. Mechanisms hypothesised to explain the generation from the [M+H-H_2_O]^+^ precursor ions of stigmasterol and stigmasterol-d_5_ of product ions with positive charge on C17 shown in Scheme S1. The C-C bonds broken during fragmentation steps are highlighted using a transversal dashed line. Exact *m/z* ratios, rounded off to the fourth decimal place, are reported.

*

*

**Figure S14**. Fragmentation pathway proposed to explain the generation of a product ion with a *m/z* ratio consistent with the exact value 297.2577 upon fragmentation of the [M+H^+^-H_2_O]^+^ ions of stigmasterol/stigmasterol-d_5_. Note that the same pathway might be considered if the positive charge was located on C3 (see Figure S15). Exact *m/z* ratios, rounded off to the fourth decimal place, are reported.


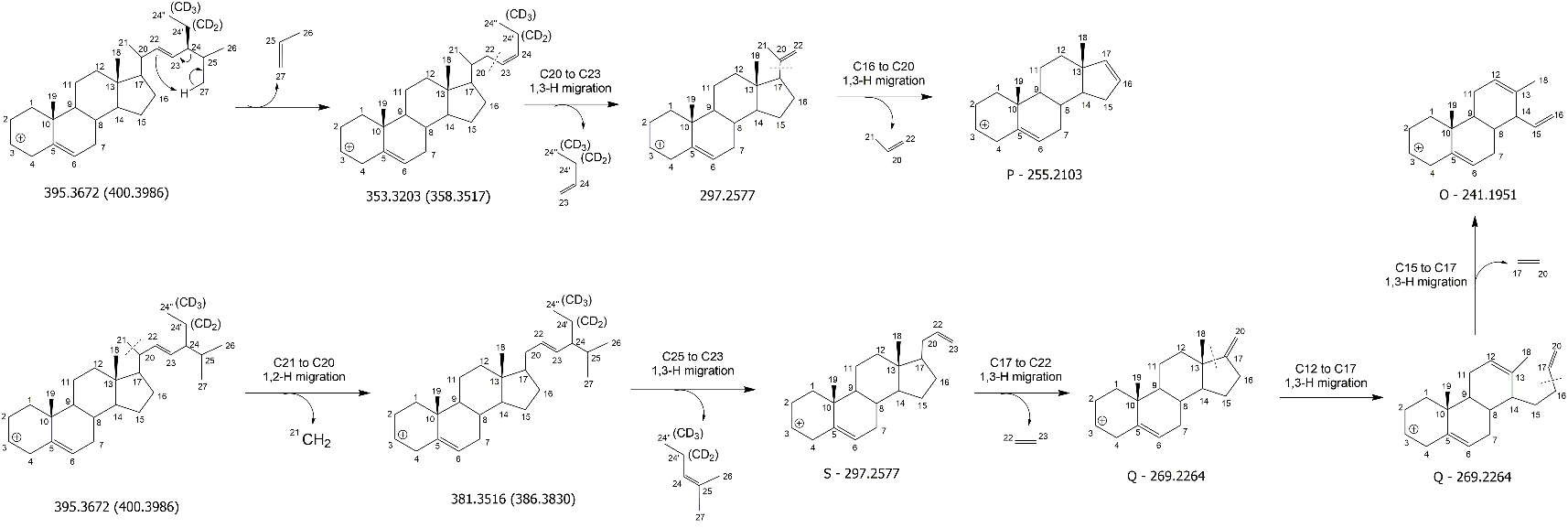

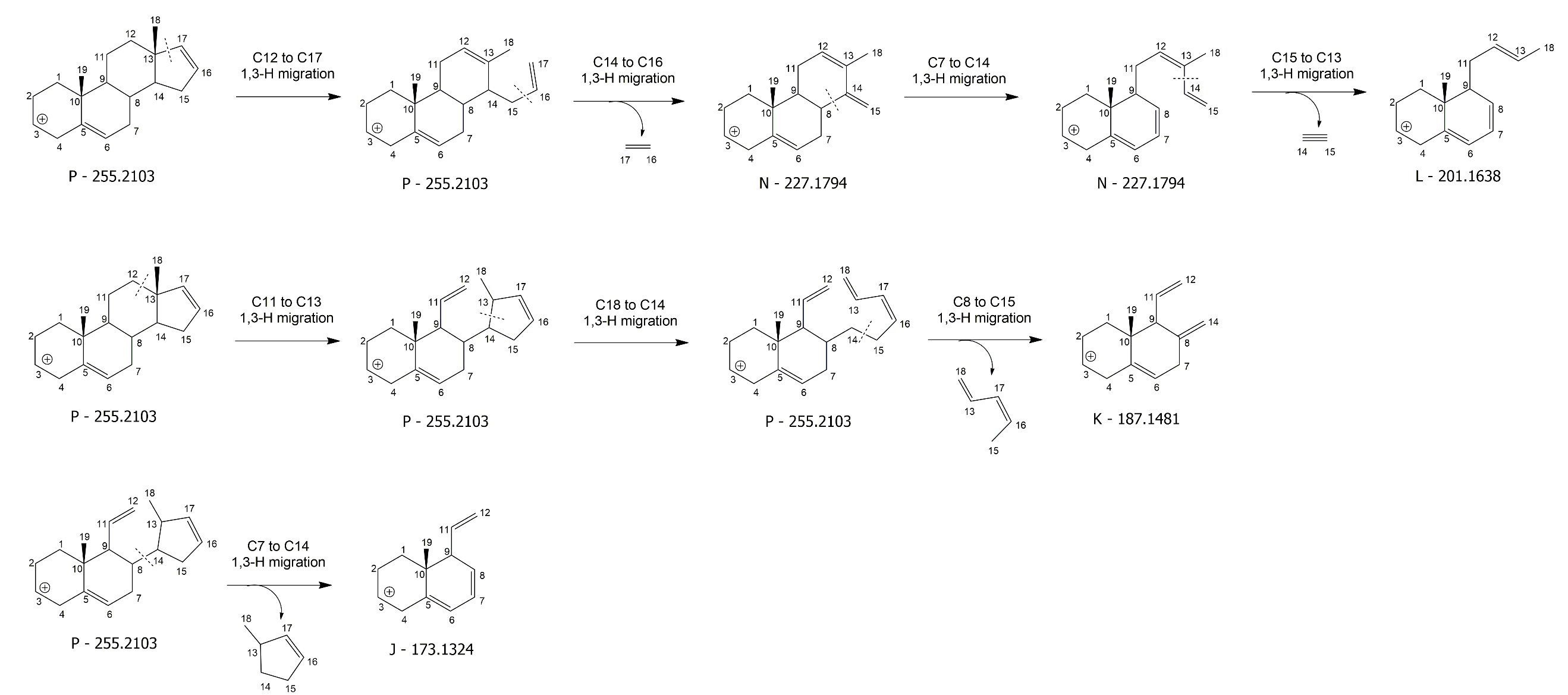


**Figure S15**. Mechanisms hypothesised to explain the generation from the [M+H-H_2_O]^+^ precursor ions of stigmasterol and stigmasterol-d_5_ of product ions with positive charge on C3 shown on the right side of Scheme S1. The C-C bonds broken during fragmentation steps are highlighted using a transversal dashed line. Exact *m/z* ratios, rounded off to the fourth decimal place, are reported.


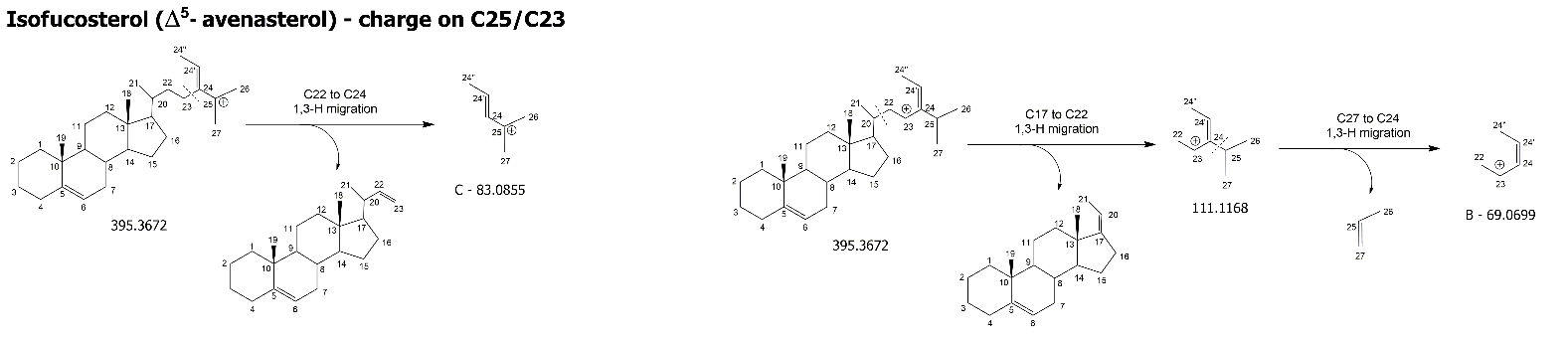

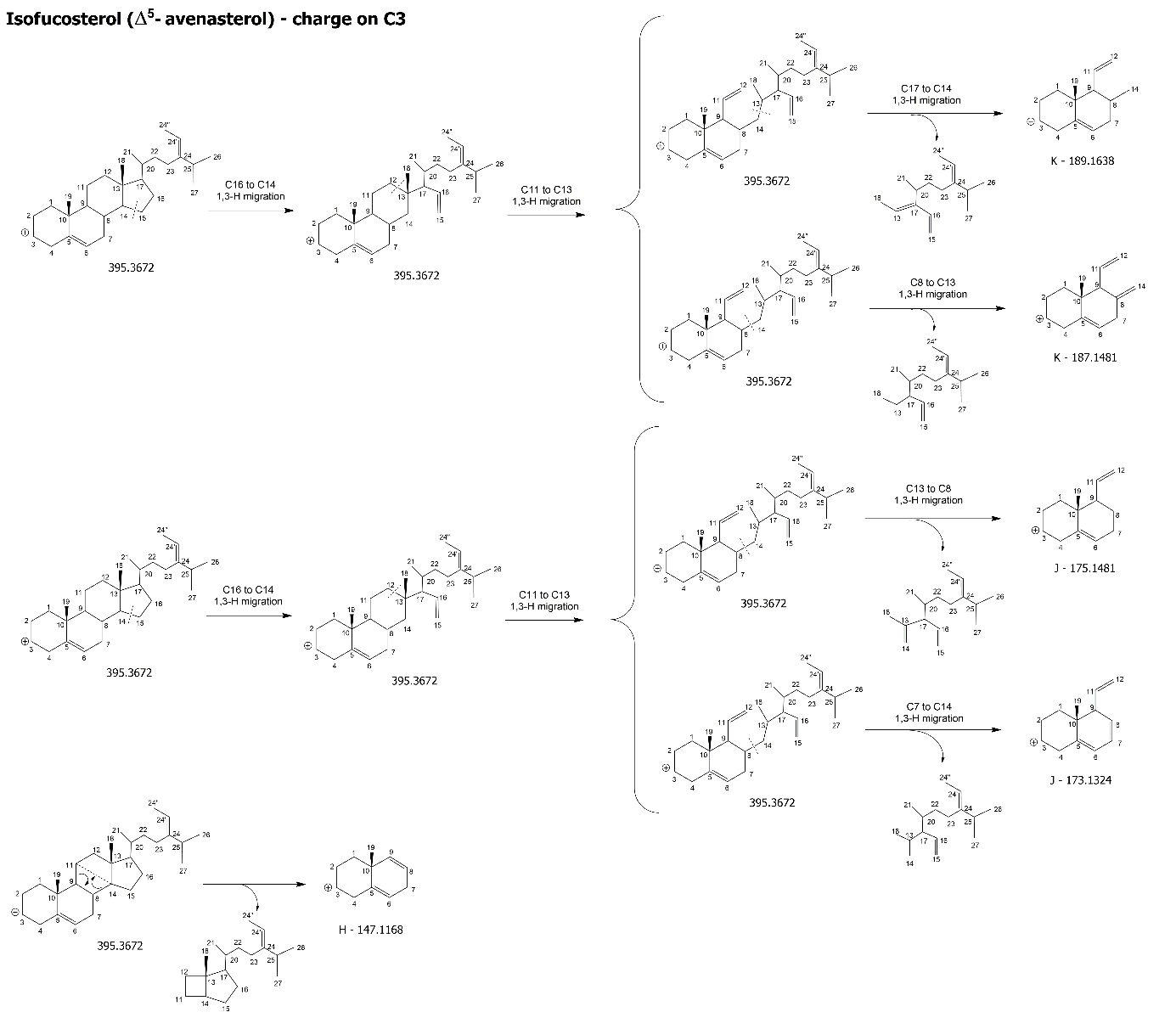

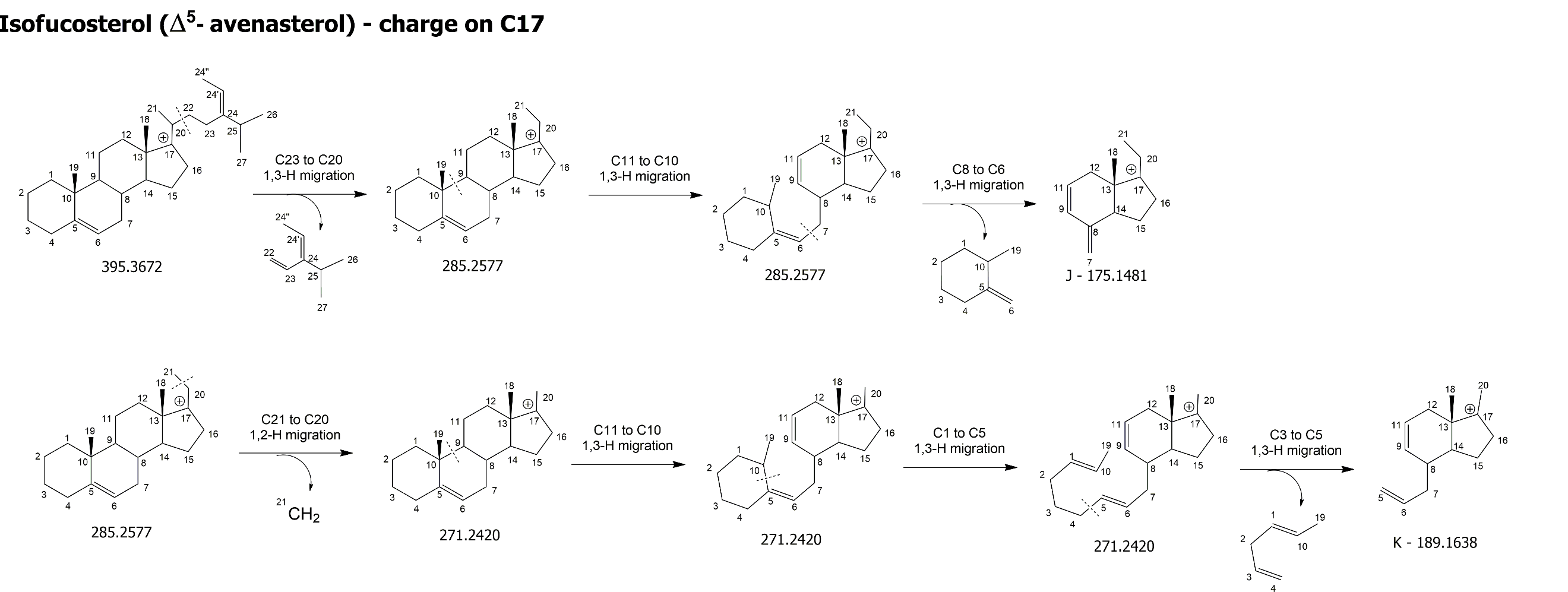


**Figure S16**. Mechanisms hypothesised to explain the generation of specific product ions arising from the [M+H-H_2_O]^+^ precursor ions of isofucosterol and reported in Scheme 3. The C-C bonds broken during fragmentation steps are highlighted using a transversal dashed line. Exact *m/z* ratios, rounded off to the fourth decimal place, are reported.


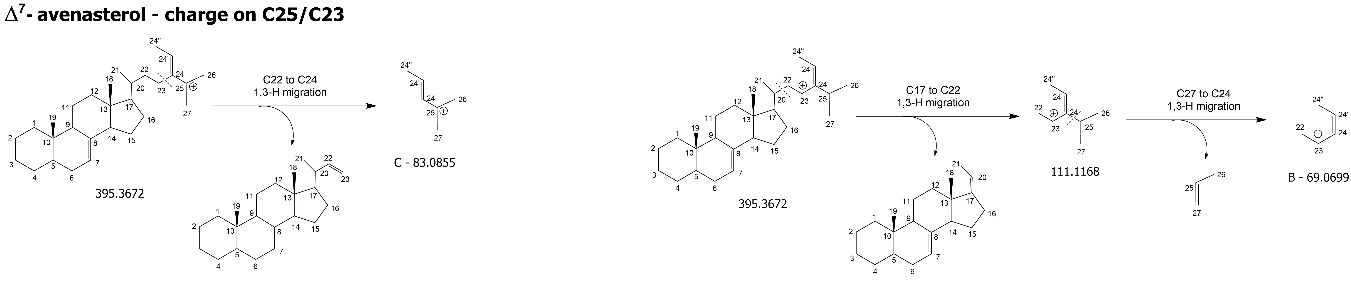

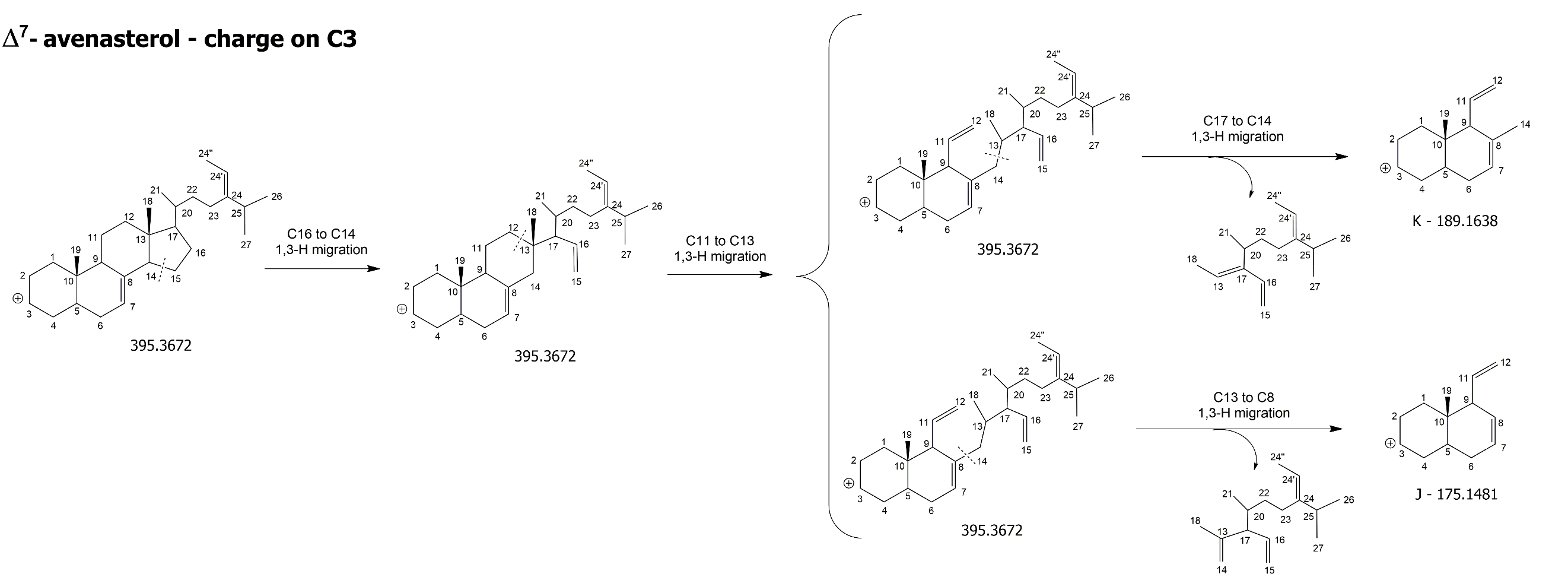

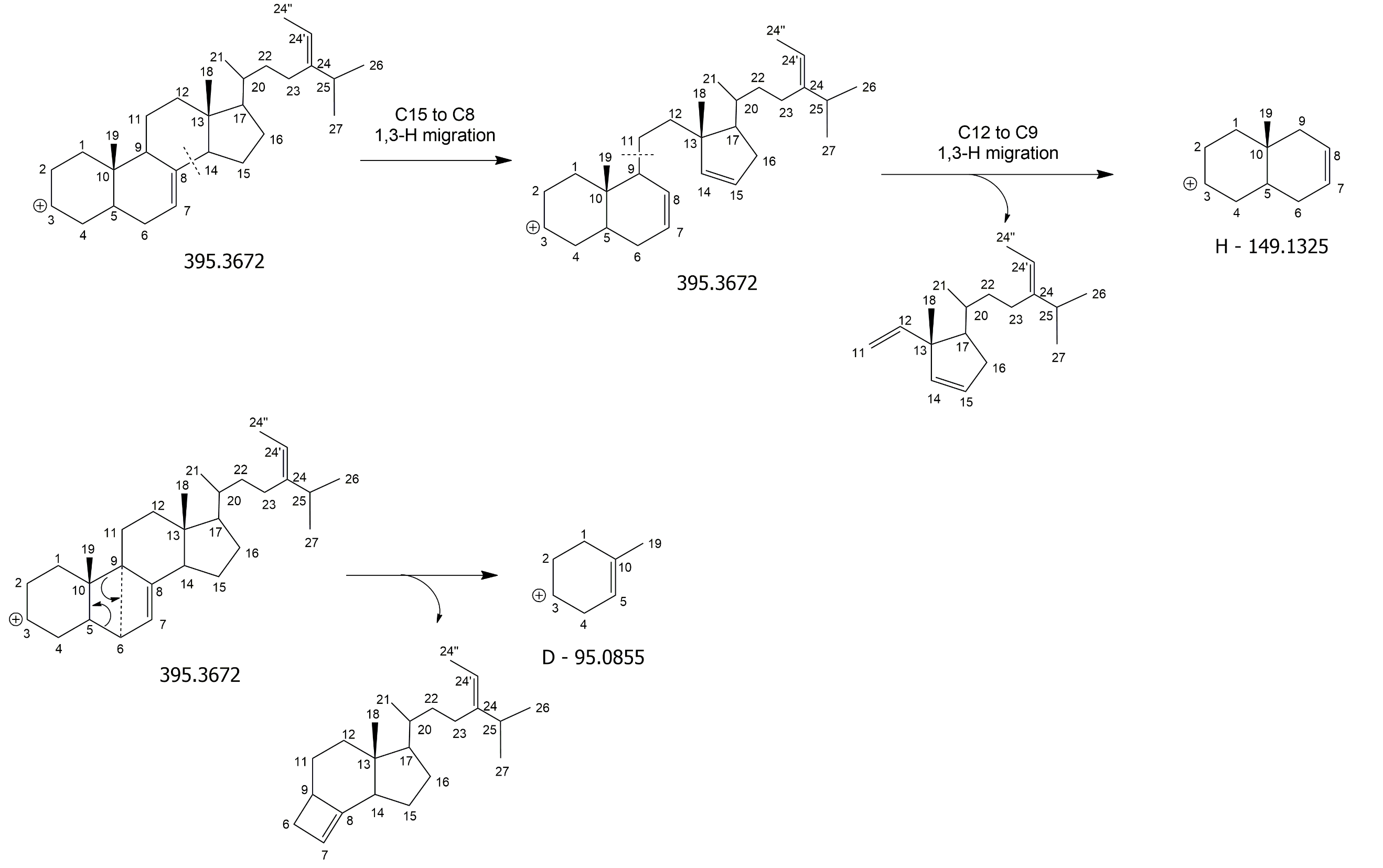

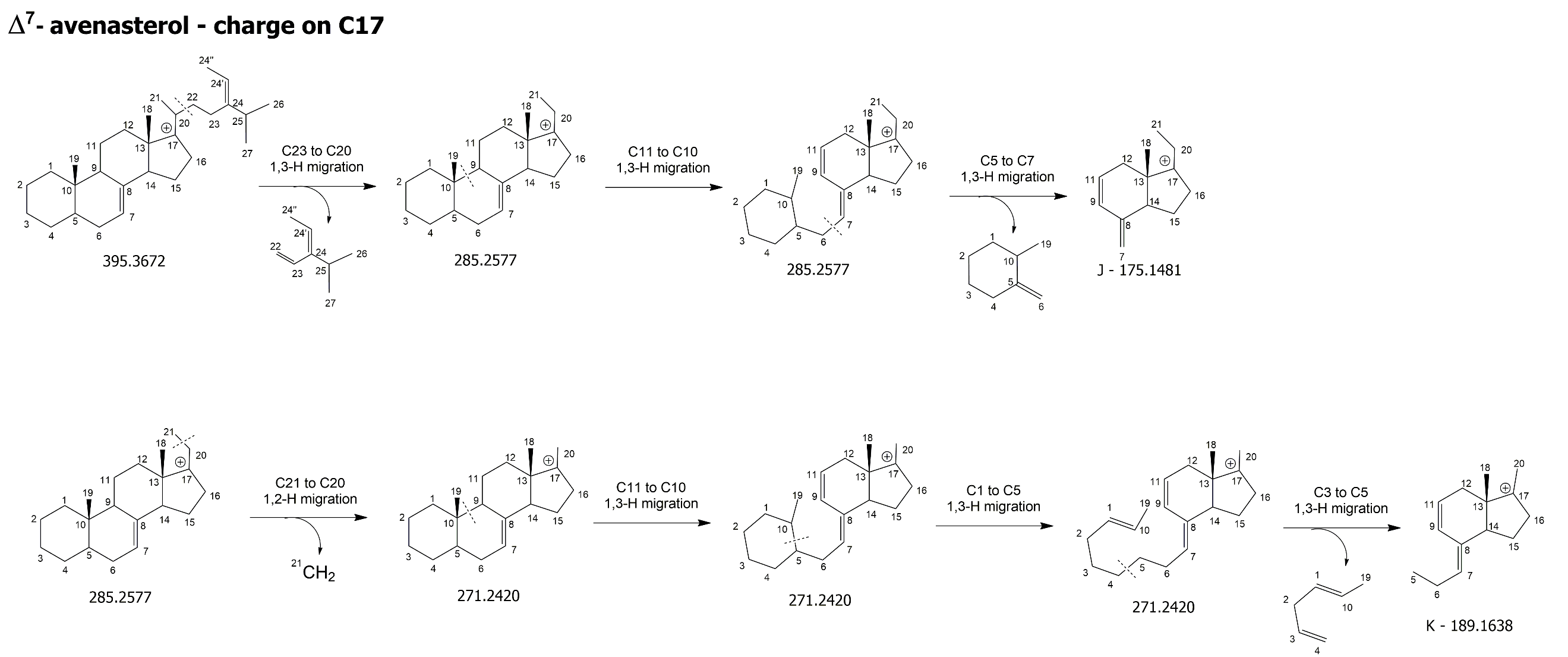


**Figure S17**. Mechanisms hypothesised to explain the generation of specific product ions arising from the [M+H-H_2_O]^+^ precursor ions of Δ^7^-avenasterol and reported in Scheme 3. The C-C bonds broken during fragmentation steps are highlighted using a transversal dashed line. Exact *m/z* ratios, rounded off to the fourth decimal place, are reported.

*

*

**Figure S18.** Alterative fragmentation pathway proposed to explain the enhanced generation of a product ion with a *m/z* ratio consistent with the exact value 83.0855 upon fragmentation of the [M+H^+^-H_2_O]^+^ ion of Δ^7^-avenasterol. Exact *m/z* ratios, rounded off to the fourth decimal place, are reported.

**

**

**Scheme S1** Fragmentation pathways hypothesised for stigmasterol and stigmasterol penta-deuterated on the ethyl group linked to C24 considering their [M+H-H_2_O]^+^ ions generated in the APCI source as precursor ions, with the positive charge located on different possible sites. Plain arrows indicate fragmentations observed in MS/MS spectra (under both HCD and CID conditions); the dashed arrow indicate fragmentations observed in the 395 > 297 > CID-MS^3^ spectrum. Boxes include multiple product ions observed in a single MS^n^ (n = 2-3) spectrum. Exact *m/z* ratios (rounded off to the fourth decimal place) are reported for all product ions. Values between parentheses are referred to deuterated stigmasterol. Note that structures hypothesized for product ions belonging to clusters from D to I were not reported since they are identical to those proposed in **Schemes 1** and **2** for cholesterol. See text for details.

**Table S1**. Experimental m/z values and relative intensities referred to peak signals detected in the HCD-FTMS/MS spectra of cholesterol and its analogue deuterated on methyl groups linked to C25 (cholesterol-d_6_). Molecular formulas consistent with m/z values (accuracy < 5 ppm) are also reported. Product ions sharing the same number of C atoms are grouped within a single cluster identified by a capital letter, as in **Figure 3**. m/z values of cholesterol product ions for which deuterated counterpart(s) were not detected at all in the cholesterol-d_6_ spectrum are underlined; those referred to product ions for which only hexa-deuterated analogues were detected in the cholesterol-d_6_ spectrum are reported with bold character.

|  | Cholesterol | | | Cholesterol-d6 | | | | |
| --- | --- | --- | --- | --- | --- | --- | --- | --- |
| Ion cluster label | **Molecular Formula** | **Experim. *m/z*** | **Rel.Int. (%)** | **Experim. *m/z*** | **Rel.Int. (%)** | **Molecular Formula** | **Experim. *m/z*** | **Rel.Int. (%)** |
| A | C_4_H_7_ | 55.0551 | <2 | 55.0550 | <2 | C_4_H_2_D_5_ | 60.0865 | 6 |
|  | C_4_H_9_ | 57.0708 | 11 | 57.0708 | <2 | C_4_H_3_D_6_ | 63.1083 | 6 |
| B | C_5_H_7_ | 67.0549 | 9 | 67.0550 | 8 | C_5_H_2_D_5_ | 72.0856 | <2 |
|  | C_5_H_9_ | 69.0706 | 15 | 69.0706 | 6 | C_5_H_4_D_5_  C_5_H_3_D_6_ | 74.1013  75.1082 | 7  3 |
|  | C_5_H_11_ | 71.0862 | 15 | 71.0862 | <2 | C_5_H_5_D_6_ | 77.1238 | 10 |
| C | C_6_H_7_ | 79.0548 | 4 | 79.0548 | 4 |  |  |  |
|  | C_6_H_9_ | 81.0705 | 39 | 81.0705 | 32 |  |  |  |
|  | C_6_H_11_ | 83.0861 | 26 | 83.0861 | 10 | C_6_H_8_D_3_  C_6_H_5_D_6_ | 86.1014  89.1237 | 6  4 |
|  | C_6_H_13_ | 85.1017 | 8 | 85.1017 | <2 | C_6_H_7_D_6_ | 91.1388 | 5 |
| D | C_7_H_7_ | 91.0546 | 4 | 91.0546 | 4 |  |  |  |
|  | C_7_H_9_ | 93.0703 | 11 | 93.0703 | 10 |  |  |  |
|  | C_7_H_11_ | 95.0859 | 49 | 95.0859 | 38 | C_7_H_5_D_6_ | 101.1235 | <2 |
|  | C_7_H_13_ | 97.1015 | 19 | 97.1015 | 7 | C_7_H_7_D_6_ | 103.1392 | 4 |
| E | C_8_H_9_ | 105.0701 | 11 | 105.0701 | 10 |  |  |  |
|  | C_8_H_11_ | 107.0858 | 16 | 107.0857 | 14 |  |  |  |
|  | C_8_H_13_ | 109.1014 | 53 | 109.1014 | 42 | C_8_H_7_D_6_ | 115.1388 | <2 |
|  | C_8_H_15_ | 111.1170 | 14 | 111.1170 | 5 | C_8_H_9_D_6_ | 117.1546 | 4 |
| F | C_9_H_11_ | 119.0856 | 13 | 119.0856 | 12 |  |  |  |
|  | C_9_H_13_ | 121.1012 | 21 | 121.1012 | 19 |  |  |  |
|  | C_9_H_15_ | 123.1168 | 26 | 123.1168 | 18 | C_9_H_9_D_6_ | 129.1545 | 2 |
|  | C_9_H_17_ | 125.1324 | 5 | 125.1324 | <2 | C_9_H_11_D_6_ | 131.1701 | 3 |
| G | C_10_H_11_ | 131.0854 | 2 | 131.0854 | 2 |  |  |  |
|  | C_10_H_13_ | 133.1010 | 19 | 133.1010 | 18 |  |  |  |
|  | C_10_H_15_ | 135.1167 | 53 | 135.1167 | 48 |  |  |  |
|  | C_10_H_17_ | 137.1323 | 10 | 137.1323 | 6 | C_10_H_11_D_6_ | 143.1699 | 3 |
| H | C_11_H_13_ | 145.1009 | 4 | 145.1010 | 4 |  |  |  |
|  | C_11_H_15_ | 147.1166 | 91 | 147.1166 | 83 |  |  |  |
|  | C_11_H_17_ | 149.1323 | 28 | 149.1323 | 24 |  |  |  |
|  | C_11_H_19_ | 151.1479 | 7 | 151.1479 | 3 | C_11_H_13_D_6_ | 157.1856 | 2 |
| I | C_12_H_15_ | 159.1166 | 6 | 159.1166 | 5 |  |  |  |
|  | C_12_H_17_ | 161.1321 | 76 | 161.1322 | 67 |  |  |  |
|  | C_12_H_19_ | 163.1475 | 14 | 163.1475 | 11 | C_12_H_13_D_6_ | 169.1856 | <2 |
|  | C_12_H_21_ | 165.1636 | 7 | 165.1636 | <2 | C_12_H_15_D_6_ | 171.2014 | 4 |
| J | C_13_H_17_ | 173.1323 | 4 | 173.1323 | 4 |  |  |  |
|  | C_13_H_19_ | 175.1478 | 33 | 175.1478 | 31 |  |  |  |
|  | C_13_H_21_ | 177.1635 | 6 | 177.1635 | 4 | C_13_H_15_D_6_ | 183.2014 | <2 |
|  | C_13_H_23_ | 179.1790 | 7 | 179.1790 | <2 | C_13_H_17_D_6_ | 185.2169 | 5 |
| K | C_14_H_19_ | 187.1477 | 6 | 187.1478 | 6 |  |  |  |
|  | C_14_H_21_ | 189.1634 | 17 | 189.1634 | 15 |  |  |  |
|  | C_14_H_23_ | 191.1790 | 4 | 191.1790 | <2 | C_14_H_17_D_6_ | 197.2168 | 3 |
|  | C_14_H_25_ | **193.1947** | 7 |  |  | C_14_H_19_D_6_ | 199.2324 | 8 |
| L | C_15_H_21_ | 201.1633 | 12 | 201.1635 | 12 |  |  |  |
|  | C_15_H_23_ | 203.1790 | 16 | 203.1790 | 14 |  |  |  |
|  | C_15_H_25_ | 205.1947 | 7 | 205.1947 | <2 | C_15_H_19_D_6_ | 211.2324 | 6 |
|  | C_15_H_27_ | **207.2103** | 4 |  |  | C_15_H_21_D_6_ | 213.2480 | 4 |
| M | C_16_H_23_ | 215.1790 | 22 | 215.1790 | 22 |  |  |  |
|  | C_16_H_25_ | 217.1946 | 10 | 217.1946 | 8 |  |  |  |
|  | C_16_H_27_ | **219.2103** | 7 |  |  | C_16_H_21_D_6_ | 225.2478 | 7 |
|  | C_16_H_29_ | **221.2257** | 7 |  |  | C_16_H_23_D_6_ | 227.2634 | <2 |
| N | C_17_H_25_ | 229.1946 | 12 | 229.1945 | 12 |  |  |  |
|  | C_17_H_27_ | 231.2102 | 2 | 231.2103 | <2 | C_17_H_21_D_6_ | 237.2478 | 2 |
|  | C_17_H_29_ | **233.2258** | 11 |  |  | C_17_H_23_D_6_ | 239.2634 | 11 |
|  | C_17_H_31_ | **235.2418** | 2 |  |  | C_17_H_25_D_6_ | 241.2791 | 2 |
| O | C_18_H_27_ | 243.2102 | 16 | 243.2102 | 16 |  |  |  |
|  | C_18_H_29_ | **245.2258** | 5 |  |  | C_18_H_23_D_6_ | 251.2634 | 5 |
|  | C_18_H_31_ | **247.2414** | 9 |  |  | C_18_H_25_D_6_ | 253.2790 | 8 |
| P | C_19_H_29_ | 257.2257 | 11 | 257.2257 | 11 |  |  |  |
|  | C_19_H_31_ | **259.2414** | 18 |  |  | C_19_H_25_D_6_ | 265.2790 | 18 |
|  | C_19_H_33_ | **261.2577** | 4 |  |  | C_19_H_27_D_6_ | 267.2946 | 4 |
| Q | C_20_H_33_ | **273.2570** | 10 |  |  | C_20_H_27_D_6_ | 279.2946 | 10 |
| R | C_21_H_33_ | **285.2564** | 2 |  |  | C_21_H_27_D_6_ | 291.2946 | 2 |
|  | C_21_H_35_ | **287.2726** | 12 |  |  | C_21_H_29_D_6_ | 293.3101 | 12 |
| S | C_22_H_35_ | 299.2725 | 2 | 299.2731 | <2 | C_22_H_29_D_6_ | 305.3101 | 2 |
| T | C_23_H_37_ | 313.2884 | 4 | 313.2884 | <2 | C_23_H_31_D_6_ | 319.3275 | 4 |
| Precursor ion | C_27_H_45_ | 369.3516 | 100 |  |  | C_27_H_39_D_6_ | 375.3892 | 100 |

**Table S2**. Experimental m/z values and relative intensities referred to peak signals detected in the HCD-FTMS/MS spectra of stigmasterol and its analogue penta-deuterated on the ethyl group linked to C24 (stigmasterol-d_5_). Molecular formulas consistent with m/z values (accuracy < 5 ppm) are also reported. Product ions sharing the same number of C atoms are grouped within a single cluster, identified by a capital letter, as in **Figure 5**. m/z values of stigmasterol product ions for which deuterated counterpart(s) were not detected at all in the stigmasterol-d_5_ spectrum are underlined; those referred to product ions for which only penta-deuterated analogues were detected in the cholesterol-d6 spectrum are reported with bold character.

|  |  | Stigmasterol | | Stigmasterol-d_5_ | | | | |
| --- | --- | --- | --- | --- | --- | --- | --- | --- |
| Ion cluster label | **Molecular Formula** | **Experim. *m/z*** | **Rel.Int. (%)** | **Experim*.m/z*** | **Rel.Int. (%)** | **Molecular Formula** | **Experim. *m/z*** | **Rel.Int. (%)** |
| A | C_4_H_7_ | 55.0551 | 4 | 55.0551 | <2 | C_4_H_4_D_3_  C_4_H_2_D_5_ | 58.0739  60.0895 | 3  4 |
|  | C_4_H_9_ | 57.0708 | 16 | 57.0707 | 3 | C_4_H_4_D_5_ | 62.1013 | <2 |
| B | C_5_H_7_ | 67.0549 | 5 | 67.0549 | 3 | C_5_H_4_D_3_ | 70.0731 | 4 |
|  | C_5_H_9_ | 69.0706 | 29 | 69.0706 | 14 | C_5_H_6_D_3_  C_5_H_4_D_5_ | 72.0877  74.1013 | 4  <2 |
|  | C_5_H_11_ | 71.0862 | 12 | 71.0862 | <2 | C_5_H_6_D_5_ | 76.1175 | 8 |
| C | C_6_H_7_ | 79.0548 | 3 | 79.0548 | 3 |  |  |  |
|  | C_6_H_9_ | 81.0705 | 32 | 81.0704 | 25 | C_6_H_5_D_4_ | 85.0950 | 12 |
|  | C_6_H_11_ | 83.0861 | 94 | 83.0861 | 9 | C_6_H_8_D_3_  C_6_H_7_D_4_  C_6_H_6_D_5_ | 86.1044  87.1111  88.1174 | 14  21  22 |
|  | C_6_H_13_ | 85.1017 | 7 |  |  | C_6_H_8_D_5_ | 90.1313 | 4 |
| D | C_7_H_7_ | 91.0546 | 2 | 91.0546 | <2 |  |  |  |
|  | C_7_H_9_ | 93.0703 | 9 | 93.0703 | 7 |  |  |  |
|  | C_7_H_11_ | 95.0859 | 33 | 95.0859 | 28 |  |  |  |
|  | C_7_H_13_ | 97.1015 | 18 | 97.1014 | <2 | C_7_H_10_D_3_  C_7_H_9_D_4_  C_7_H_8_D_5_ | 100.1200  101.1263  102.1326 | <2  <2  4 |
| E | C_8_H_9_ | 105.0701 | 8 | 105.0701 | 6 |  |  |  |
|  | C_8_H_11_ | 107.0857 | 15 | 107.0857 | 16 |  |  |  |
|  | C_8_H_13_ | 109.1014 | 29 | 109.1014 | 22 | C_8_H_8_D_5_ | 114.1326 | <2 |
|  | C_8_H_15_ | 111.1170 | 6 | 111.1170 | <2 | C_8_H_10_D_5_ | 116.1482 | 3 |
| F | C_9_H_11_ | 119.0856 | 8 | 119.0855 | 7 |  |  |  |
|  | C_9_H_13_ | 121.1012 | 17 | 121.1012 | 16 |  |  |  |
|  | C_9_H_15_ | 123.1168 | 16 | 123.1168 | 12 | C_9_H_10_D_5_ | 128.1482 | 4 |
|  | C_9_H_17_ | 125.1324 | 2 | 125.1324 | <2 | C_9_H_12_D_5_ | 130.1639 | 2 |
| G | C_10_H_11_ | 131.0854 | 5 | 131.0855 | 4 |  |  |  |
|  | C_10_H_13_ | 133.1010 | 14 | 133.1010 | 14 |  |  |  |
|  | C_10_H_15_ | 135.1167 | 27 | 135.1166 | 27 | C_10_H_12_D_3_ | 140.1478 | <2 |
|  | C_10_H_17_ | 137.1323 | 7 | 137.1322 | <2 | C_10_H_12_D_5_ | 142.1639 | 3 |
| H | C_11_H_13_ | 145.1010 | 11 | 145.1009 | 10 |  |  |  |
|  | C_11_H_15_ | 147.1166 | 48 | 147.1165 | 45 |  |  |  |
|  | C_11_H_17_ | 149.1323 | 22 | 149.1322 | 21 | C_11_H_14_D_3_ | 154.1639 | <2 |
|  | C_11_H_19_ | 151.1479 | 3 | 151.148 | <2 | C_11_H_14_D_5_ | 156.1795 | 2 |
| I | C_12_H_15_ | 159.1166 | 23 | 159.1165 | 22 |  |  |  |
|  | C_12_H_17_ | 161.1321 | 37 | 161.1321 | 35 |  |  |  |
|  | C_12_H_19_ | 163.1475 | 14 | 163.1475 | 12 | C_12_H_14_D_5_ | 168.1790 | <2 |
|  | C_12_H_21_ | 165.1636 | 2 | 165.1636 | <2 | C_12_H_16_D_5_ | 170.1952 | 2 |
| J | C_13_H_17_ | 173.1323 | 21 | 173.1322 | 21 |  |  |  |
|  | C_13_H_19_ | 175.1478 | 16 | 175.1478 | 16 |  |  |  |
|  | C_13_H_21_ | 177.1635 | 8 | 177.1634 | 5 | C_13_H_16_D_5_ | 182.1952 | <2 |
|  | C_13_H_23_ | **179.1790** | 2 |  |  | C_13_H_18_D_5_ | 184.2105 | 2 |
| K | C_14_H_17_ | 185.1321 | 4 | 185.1321 | 4 |  |  |  |
|  | C_14_H_19_ | 187.1478 | 19 | 187.1477 | 18 |  |  |  |
|  | C_14_H_21_ | 189.1634 | 13 | 189.1633 | 11 | C_14_H_18_D_3_ | 194.1946 | <2 |
|  | C_14_H_23_ | 191.1790 | 3 | 191.1790 | <2 | C_14_H_18_D_5_ | 196.2104 | 2 |
| L | C_15_H_19_ | 199.1478 | 7 | 199.1478 | 7 |  |  |  |
|  | C_15_H_21_ | 201.1633 | 23 | 201.1633 | 22 |  |  |  |
|  | C_15_H_23_ | 203.1790 | 13 | 203.1790 | 12 | C_15_H_20_D_3_ | 208.2103 | <2 |
|  | C_15_H_25_ | **205.1947** | 2 |  |  | C_15_H_20_D_5_ | 210.2259 | <2 |
| M | C_16_H_21_ | 213.1632 | 15 | 213.1632 | 15 |  |  |  |
|  | C_16_H_23_ | 215.1790 | 21 | 215.1789 | 20 |  |  |  |
|  | C_16_H_25_ | 217.1946 | 4 | 217.1945 | 3 | C_16_H_20_D_5_ | 222.2260 | <2 |
| N | C_17_H_23_ | 227.1788 | 10 | 227.1788 | 10 |  |  |  |
|  | C_17_H_25_ | 229.1946 | 9 | 229.1945 | 8 |  |  |  |
|  | C_17_H_27_ | 231.2102 | 2 | 231.2102 | <2 | C_17_H_22_D_5_ | 236.2414 | 2 |
|  | C_17_H_29_ | **233.2258** | 2 |  |  | C_17_H_24_D_5_ | 238.2570 | <2 |
| O | C_18_H_25_ | 241.1944 | 14 | 241.1944 | 14 |  |  |  |
|  | C_18_H_27_ | 243.2102 | 4 | 243.2101 | 2 |  |  |  |
|  | C_18_H_29_ | **245.2258** | 2 |  |  | C_18_H_24_D_5_ | 250.2570 | 2 |
|  | C_18_H_31_ | **247.2414** | 2 |  |  | C_18_H_26_D_5_ | 252.2728 | 2 |
| P | C_19_H_27_ | 255.2107 | 23 | 255.2107 | 22 |  |  |  |
|  | C_19_H_29_ | 257.2257 | 4 | 257.2257 | 2 |  |  |  |
|  | C_19_H_31_ | **259.2414** | 3 |  |  | C_19_H_26_D_5_ | 264.2726 | <2 |
|  | C_19_H_33_ | **261.2570** | 2 |  |  | C_19_H_28_D_5_ | 266.2883 | <2 |
| Q | C_20_H_29_ | 269.2257 | 8 | 269.2257 | 8 |  |  |  |
|  | C_20_H_31_ | 271.2413 | 3 | 271.2413 | <2 | C_20_H_28_D_3_ | 276.2725 | 2 |
|  | C_20_H_33_ | **273.2570** | 3 |  |  | C_20_H_28_D_5_ | 278.2883 | 2 |
| R | C_21_H_31_ | 283.2412 | 10 | 283.2412 | 10 |  |  |  |
|  | C_21_H_33_ | **285.2564** | 8 |  |  | C_21_H_28_D_5_ | 290.2882 | 7 |
|  | C_21_H_35_ | **287.2726** | 3 |  |  | C_21_H_30_D_5_ | 292.3039 | 3 |
| S | C_22_H_33_ | 297.2568 | 32 | 297.2568 | 30 |  |  |  |
|  | C_22_H_35_ | **299.2725** | 5 |  |  | C_22_H_30_D_5_ | 304.3038 | 5 |
| T | C_23_H_35_ | 311.2575 | 15 | 311.2725 | 15 |  |  |  |
|  | C_23_H_37_ | **313.2884** | 6 |  |  | C_23_H_32_D_5_ | 318.3195 | 6 |
| U | C_24_H_37_ | 325.2883 | 2 | 325.2883 | <2 | C_24_H_32_D_5_ | 330.3198 | 2 |
| V | C_25_H_39_ | **339.3046** | 4 |  |  | C_23_H_34_D_5_ | 344.3352 | 4 |
| Precursor ion | C_29_H_47_ | 395.3672 | 100 |  |  | C_27_H_40_D_5_ | 400.3987 | 100 |

**Table S3.** Summary of typical relative intensities obtained for specific ions detected in APCI(+)-HCD-FTMS/MS spectra acquired for the [M+H-H_2_O]^+^ ions of sterols investigated in the present study. Experimental m/z values and molecular formulas inferred from them considering an accuracy not higher than 5 ppm are also reported. Ions are grouped according to the number of C atoms in their structures, in clusters labelled as in **Figures 3**, **4** and **5**, according to the case. The prevailing relative intensity in each cluster is reported in bold character.

|  |  |  | **Cholesterol** | **Campesterol** | **β-sitosterol** | **Stigmasterol** | **Brassicasterol** | **Isofucosterol** | **Δ^7^-avenasterol** |
| --- | --- | --- | --- | --- | --- | --- | --- | --- | --- |
| **Ion cluster** | **Molecular formula** | **Experim. *m/z*** | **Relative Intensity [%]** | | | | | | |
| **A** | C_4_H_7_ | 55.0551 | <2 | 2 | <2 | 4 | <2 | 2 | 6 |
|  | C_4_H_9_ | 57.0708 | **11** | **16** | **18** | **16** | **11** | **10** | **15** |
| **B** | C_5_H_7_ | 67.0549 | 9 | 9 | 9 | 5 | 4 | 7 | 14 |
|  | C_5_H_9_ | 69.0708 | 15 | 13 | 21 | **29** | **51** | **39** | **66** |
|  | C_5_H_11_ | 71.0862 | **15** | **21** | **27** | 12 | 5 | 13 | 15 |
| **C** | C_6_H_7_ | 79.0548 | 4 | 3 | 4 | 3 | 2 | 3 | 8 |
|  | C_6_H_9_ | 81.0705 | **39** | **39** | **40** | 32 | 20 | **32** | 64 |
|  | C_6_H_11_ | 83.0861 | 26 | 23 | 22 | **94** | **22** | 25 | **89** |
|  | C_6_H_13_ | 85.1017 | 8 | 15 | 22 | 7 | 3 | 9 | 11 |
| **D** | C_7_H_7_ | 91.0546 | 4 | 3 | 3 | 2 | / | 3 | 7 |
|  | C_7_H_9_ | 93.0703 | 11 | 12 | 13 | 9 | 6 | 11 | 28 |
|  | C_7_H_11_ | 95.0859 | **49** | **49** | **50** | **33** | **26** | **39** | **80** |
|  | C_7_H_13_ | 97.1015 | 19 | 24 | 21 | 18 | 9 | 20 | 35 |
| **E** | C_8_H_9_ | 105.0701 | 11 | 11 | 11 | 8 | / | 10 | 25 |
|  | C_8_H_11_ | 107.0858 | 16 | 15 | 17 | 15 | 11 | 17 | 45 |
|  | C_8_H_13_ | 109.1014 | **53** | **52** | **52** | **29** | **24** | **40** | **70** |
|  | C_8_H_15_ | 111.1170 | 14 | 14 | 19 | 6 | 5 | 11 | 18 |
| **F** | C_9_H_11_ | 119.0856 | 13 | 12 | 13 | 8 | 7 | 10 | 23 |
|  | C_9_H_13_ | 121.1012 | 21 | 21 | 24 | **17** | **13** | **24** | **59** |
|  | C_9_H_15_ | 123.1168 | **26** | **26** | **27** | 16 | 12 | 22 | 41 |
|  | C_9_H_17_ | 125.1324 | 5 | 5 | 6 | 2 | 5 | 5 | 4 |
| **G** | C_10_H_11_ | 131.0854 | 2 | / | / | 5 | 4 | 6 | 10 |
|  | C_10_H_13_ | 133.1010 | 19 | 19 | 20 | 14 | 11 | 18 | 35 |
|  | C_10_H_15_ | 135.1167 | **53** | **54** | **56** | **27** | **20** | **35** | **73** |
|  | C_10_H_17_ | 137.1323 | 10 | 13 | 13 | 7 | 5 | 13 | 31 |
| **H** | C_11_H_13_ | 145.1009 | 4 | 4 | 4 | 11 | 9 | 13 | 21 |
|  | C_11_H_15_ | 147.1166 | **91** | **91** | **95** | **48** | **34** | **66** | 62 |
|  | C_11_H_17_ | 149.1323 | 28 | 30 | 31 | 22 | 17 | 28 | **93** |
|  | C_11_H_19_ | 151.1479 | 7 | 8 | 9 | 3 | 2 | 6 | 8 |
| **I** | C_12_H_13_ | 157.1012 | / | / | / | / | / | 2 | 2 |
|  | C_12_H_15_ | 159.1166 | 6 | 6 | 6 | 23 | 20 | 41 | 49 |
|  | C_12_H_17_ | 161.1321 | **76** | **77** | **80** | **37** | **29** | **65** | **92** |
|  | C_12_H_19_ | 163.1475 | 14 | 16 | 17 | 14 | 12 | 18 | 43 |
|  | C_12_H_21_ | 165.1636 | 7 | 7 | 6 | 2 | 2 | 4 | 4 |
| **J** | C_13_H_15_ | 171.1168 | / | / | / | / | / | 2 | 2 |
|  | C_13_H_17_ | 173.1323 | 4 | 4 | 4 | **21** | **17** | 26 | 27 |
|  | C_13_H_19_ | 175.1478 | **33** | **36** | **38** | 16 | 13 | **28** | **53** |
|  | C_13_H_21_ | 177.1635 | 6 | 7 | 7 | 8 | 6 | 8 | 19 |
|  | C_13_H_23_ | 179.1790 | 7 | 4 | 3 | 2 | / | / | / |
| **K** | C_14_H_17_ | 185.1321 | / | / | / | 4 | 3 | 4 | 5 |
|  | C_14_H_19_ | 187.1477 | 6 | 7 | 7 | **19** | **14** | **14** | 31 |
|  | C_14_H_21_ | 189.1633 | **17** | **20** | **21** | 13 | 10 | 13 | **36** |
|  | C_14_H_23_ | 191.179 | 4 | 3 | 3 | 3 | 3 | 5 | 6 |
|  | C_14_H_25_ | 193.1947 | 7 | 3 | 2 | / | / | / | / |
| **L** | C_15_H_19_ | 199.1477 | / | / | / | 7 | 7 | 8 | 8 |
|  | C_15_H_21_ | 201.1633 | 12 | 12 | 12 | **23** | **17** | **15** | **37** |
|  | C_15_H_23_ | 203.1790 | **16** | **19** | **19** | 13 | 10 | **15** | 32 |
|  | C_15_H_25_ | 205.1947 | 7 | / | / | 2 | 2 | 3 | 4 |
|  | C_15_H_27_ | 207.2103 | 4 | 5 | 2 | / | / | / | / |
| **M** | C_16_H_21_ | 213.1633 | / | / | / | 15 | 13 | 12 | 14 |
|  | C_16_H_23_ | 215.1790 | **22** | **23** | **23** | **21** | **16** | **18** | **58** |
|  | C_16_H_25_ | 217.1946 | 10 | 11 | 11 | 4 | 4 | 5 | 15 |
|  | C_16_H_27_ | 219.2103 | 7 | 4 | / | / | 3 | 2 | 4 |
|  | C_16_H_29_ | 221.2257 | 7 | 2 | 4 | / | / | / | / |
| **N** | C_17_H_23_ | 227.1789 | / | / | / | **10** | **8** | 7 | 13 |
|  | C_17_H_25_ | 229.1946 | **12** | **13** | **13** | 9 | 7 | **10** | **29** |
|  | C_17_H_27_ | 231.2102 | 2 | / | / | 2 | 3 | 4 | 7 |
|  | C_17_H_29_ | 233.2258 | 11 | 5 | 4 | 2 | 2 | 2 | 5 |
|  | C_17_H_31_ | 235.2418 | 2 | / | / | / | / | / | / |
| **O** | C_18_H_25_ | 241.1944 | / | / | / | **14** | **12** | **10** | **22** |
|  | C_18_H_27_ | 243.2102 | **16** | **19** | **19** | 4 | 3 | 5 | 12 |
|  | C_18_H_29_ | 245.2258 | 5 | / | / | 2 | 3 | 2 | 5 |
|  | C_18_H_31_ | 247.2414 | 9 | 9 | 5 | 2 | 2 | 3 | / |
| **P** | C_19_H_27_ | 255.2107 | / | / | / | **23** | **17** | **20** | **31** |
|  | C_19_H_29_ | 257.2257 | 11 | **14** | **16** | 4 | 4 | 8 | 28 |
|  | C_19_H_31_ | 259.2414 | **18** | 10 | / | 3 | 3 | 5 | 3 |
|  | C_19_H_33_ | 261.2577 | 4 | 7 | 8 | 2 | / | / | / |
| **Q** | C_20_H_29_ | 269.2257 | / | / | / | **8** | 6 | 4 | 7 |
|  | C_20_H_31_ | 271.2413 | **/** | 2 | 3 | 3 | **8** | **6** | **13** |
|  | C_20_H_33_ | 273.2570 | **10** | **15** | 4 | 3 | 4 | 5 | / |
|  | C_20_H_35_ | 275.2726 | **/** | 4 | **6** | / | / | / | / |
| **R** | C_21_H_31_ | 283.2412 | **/** | **/** | **/** | **10** | **6** | **10** | **22** |
|  | C_21_H_33_ | 285.2564 | 2 | 3 | 3 | 8 | 5 | 8 | 9 |
|  | C_21_H_35_ | 287.2726 | **12** | **8** | **14** | 3 | **/** | **/** | **/** |
|  | C_21_H_37_ | 289.2890 | **/** | **/** | 3 | **/** | **/** | **/** | **/** |
| **S** | C_22_H_33_ | 297.2568 | / | / | / | **32** | **25** | **13** | **55** |
|  | C_22_H_35_ | 299.2725 | **2** | 3 | 3 | 5 | 6 | 4 | 5 |
|  | C_22_H_37_ | 301.2880 | / | **10** | **7** | / | / | / | / |
| **T** | C_23_H_35_ | 311.2725 | / | / | / | **15** | **10** | **10** | 9 |
|  | C_23_H_37_ | 313.2884 | **4** | **2** | 2 | 6 | / | 5 | **18** |
|  | C_23_H_39_ | 315.3041 | / | / | **9** | / | / | / | / |
| **U** | C_24_H_37_ | 325.2881 | / | / | / | **2** | **3** | **3** | 4 |
|  | C_24_H_39_ | 327.3039 | / | **3** | **2** | / | / | / | **7** |
| **V** | C_25_H_39_ | 339.3046 | / | / | / | **4** | / | **3** | **4** |
|  | C_25_H_41_ | 341.3203 | / | / | **3** | / | / | / | / |
| **Precursor ion** | C_27_H_45_ | 369.3516 | **100** | / | / | / | / | / | / |
|  | C_28_H_45_ | 381.3516 | / | / | / | / | **100** | / | / |
|  | C_28_H_47_ | 383.3672 | / | **100** | / | / | / | / | / |
|  | C_29_H_47_ | 395.3672 | / | / | / | **100** | / | **100** | **100** |
|  | C_29_H_49_ | 397.3829 | / | / | **100** | / | / | / | / |
